# Usefulness of Midregional Proadrenomedullin to Predict Poor Outcome in Patients with Community Acquired Pneumonia

**DOI:** 10.1371/journal.pone.0125212

**Published:** 2015-06-01

**Authors:** Susana Gordo-Remartínez, María Calderón-Moreno, Juan Fernández-Herranz, Ana Castuera-Gil, Mar Gallego-Alonso-Colmenares, Carolina Puertas-López, José A. Nuevo-González, Domingo Sánchez-Sendín, Mercedes García-Gámiz, José A. Sevillano-Fernández, Luis A. Álvarez-Sala, Juan A. Andueza-Lillo, José M. de Miguel-Yanes

**Affiliations:** 1 Emergency Department, Hospital General Universitario “Gregorio Marañón”, Madrid, Spain; 2 Biochemical Department, Hospital General Universitario “Gregorio Marañón”, Madrid, Spain; 3 Internal Medicine Department, Hospital General Universitario “Gregorio Marañón”, Madrid, Spain; 4 Department of Medicine. Facultad de Medicina. Universidad Complutense de Madrid, Spain; University of Dundee, UNITED KINGDOM

## Abstract

**Background:**

midregional proadrenomedullin (MR-proADM) is a prognostic biomarker in patients with community-acquired pneumonia (CAP). We sought to confirm whether MR-proADM added to Pneumonia Severity Index (PSI) improves the potential prognostic value of PSI alone, and tested to what extent this combination could be useful in predicting poor outcome of patients with CAP in an Emergency Department (ED).

**Methods:**

Consecutive patients diagnosed with CAP were enrolled in this prospective, single-centre, observational study. We analyzed the ability of MR-proADM added to PSI to predict poor outcome using receiver operating characteristic (ROC) curves, logistic regression and risk reclassification and comparing it with the ability of PSI alone. The primary outcome was “poor outcome”, defined as the incidence of an adverse event (ICU admission, hospital readmission, or mortality at 30 days after CAP diagnosis).

**Results:**

226 patients were included; 33 patients (14.6%) reached primary outcome. To predict primary outcome the highest area under curve (AUC) was found for PSI (0.74 [0.64-0.85]), which was not significantly higher than for MR-proADM (AUC 0.72 [0.63-0.81, p > 0.05]). The combination of PSI and MR-proADM failed to improve the predictive potential of PSI alone (AUC 0.75 [0.65-0.85, p=0.56]). Ten patients were appropriately reclassified when the combined PSI and MR-proADM model was used as compared with the model of PSI alone. Net reclassification improvement (NRI) index was statistically significant (7.69%, p = 0.03) with an improvement percentage of 3.03% (p = 0.32) for adverse event, and 4.66% (P = 0.02) for no adverse event.

**Conclusion:**

MR-proADM in combination with PSI may be helpful in individual risk stratification for short-term poor outcome of CAP patients, allowing a better reclassification of patients compared with PSI alone.

## Introduction

Community-acquired pneumonia (CAP) is the third most frequently diagnosed infection in the emergency departments (ED) [1–3]. CAP ranges from a mild affection that can be treated at home to a severe disease. In fact, CAP is one of the leading causes of death in developed countries [4–10]. An early identification of patients with a higher risk for complications could lead to both a reduction of medical costs and deaths. Clinical practice guidelines recommend the use of severity prediction rules to choose among the adequate empirical antibiotic regimen, the intensity of the requested complementary studies, and need of intensive care to finally decide whether treatment can be followed on an outpatient basis. All these recommendations facilitate decision making in the ED [11–13]. In this way, the Pneumonia Severity Index (PSI) [14] is the most widely validated score [15] despite having some limitations [16].

The potential prognostic usefulness of several biomarkers for patients with CAP has been explored in recent years. Proadrenomedullin (ProADM) is a peptide with vasodilatory, antimicrobial and anti-inflammatory properties. Specifically, its midregional fragment (MR-proADM) has been associated with mortality in patients with CAP [17]. Predicting the risk of death is crucial in the ED, but at the same time, the level of care required by patients cannot be assessed only in terms of mortality. A better knowledge of those patients with a higher risk of needing intensive care or those with a higher risk of being admitted after discharge could also help in the decision making. Concerning this, six studies have analysed the role of MR-proADM to predict complications in patients with CAP, but only three of them have tested its usefulness as compared with that of PSI, and none of them studied the risk of readmission of those patients [18–23].

The main objective of this study is to confirm whether MR-proADM added to PSI improves the potential prognostic value of PSI alone, and to what extent this combination could be useful in predicting poor outcome of patients with CAP.

## Materials and Methods

### Setting and study population

The NACURG (**N**eumonía **A**dquirida en la **C**omunidad en **URG**encias; in English, Community-Acquired Pneumonia at the ED) is an observational, prospective, single-centre cohort study in patients diagnosed with CAP at the ED of the Gregorio Marañón University Hospital, between November 2012 and March 2013. This institution is a university tertiary-care public hospital of the Community of Madrid (Spain) with a reference population of 318,000 people that attended 148,000 emergencies last year (pediatric and gynecological emergencies are not included).

**Fig 1:**
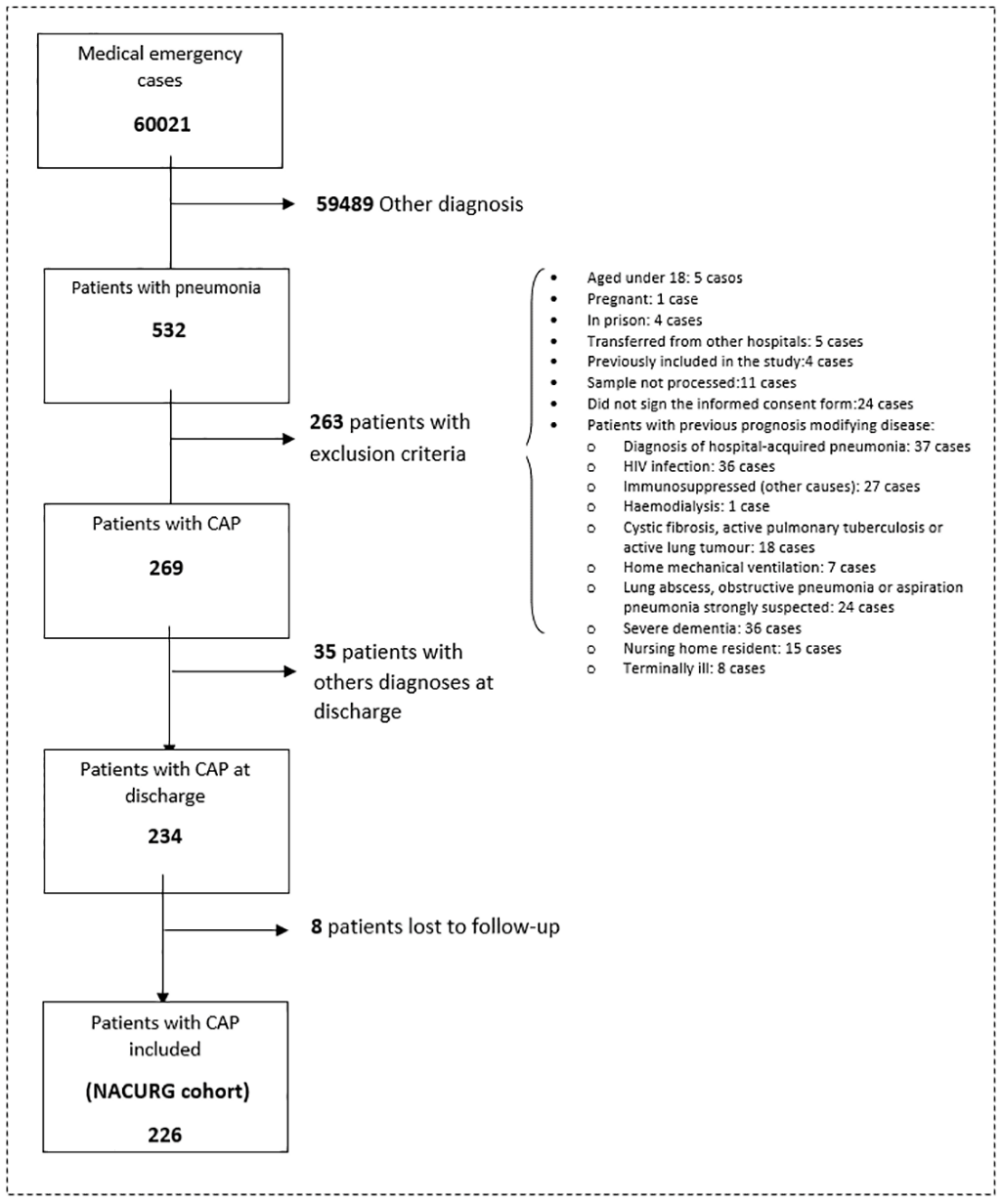
Patients included in the study.

All consecutive patients diagnosed with CAP in the ED were offered to participate in the study. Pneumonia was defined as the presence of at least one of the following symptoms: cough, expectoration, dyspnea, chest pain, fever, abnormal lung auscultation or leukocytes > 10,000 or < 4,000 cells/μL in combination with a new infiltrate on a chest X-ray [9, 23]. The excluded cases were: pneumonia due to nosocomial origin (more than 48 hours of hospitalization in the last 30 days), patients with health-care associated pneumonia or patients whose baseline medical conditions could have conditioned the prognosis of pneumonia. The process of patients’ selection is summarised in Fig 1.

Before the beginning of the study, several informative meetings were conducted among medical assistants and residents of the ED. When a diagnosis of pneumonia was made at the ED, a researcher was contacted 24 hours a day to assess whether the patients met the inclusion criteria. Where it was possible for a conscious and comprehending patient to give informed consent to take part in this study, the study was explained verbally to that patient by the investigator or research coordinator and the patient was given the opportunity to read the participant information sheet. After they had had their questions answered and if they were willing to take part in the study, they were asked to sign the consent form. Clinical assessment of the competence of a potential participant to consent for research was undertaken by a treating Emergency clinician. Emergency physicians are experienced at evaluating the competence of their patients to understand their illness and consent for therapeutic interventions. Specific situations that precluded potential participants to sign informed consents by themselves were: previous cognitive impairment, a decreased consciousness or medical instability judged to limit their ability to understand the purpose of the intervention or to give an unbiased consent to be part of the study. Where it was not practicable to approach the participant, consent was obtained from the “next of kin”, term that describes the person who is legally allowed to give consent for the patient. When the next of kin could not attend the hospital to receive information and sign the consent form in person, the participant was finally not included in the study. After signing the informed consent, the patient was enrolled in the study. In all cases, patients were treated by the Emergency clinician without knowing the MR-proADM results. Patients’ follow-up was done through their computerised medical records and phone calls after 30 and 90 days of consultation to the ED. No therapeutic interventions were considered in the study. It was approved by the Hospital Ethic Committee (CEIC Hospital General Universitario Gregorio Marañón 278/12).

### Outcomes and measurements

The main outcome, namely poor outcome of CAP, was measured through a composite variable defined as the existence of an adverse event: ICU admission, hospital readmission, or mortality at 30 days after CAP diagnosis in the ED (short-term mortality). Other secondary outcomes were: mortality due to any cause at 90 days after CAP diagnosis in the ED (mid-term mortality).

The independent variables were:

- Epidemiological data, Charlson index of comorbidity [24], symptoms of the current disease and previous antibiotic treatment. Exploratory findings and routine analytical and radiological data were prospectively recorded by the attending physicians of the ED.
- Microbiological tests: according to the SEPAR (Spanish Society of Respiratory Diseases) guidelines [11], we requested 3 blood culture samples using bottles with liquid media for each hospitalised patient (with or without measurable fever). The following tests were done: fluorometric measurements using Bactec system for pneumococcus and *Legionella pneumophila* through the BinaxNOW immunochromatographic membrane test; in epidemical situation or in cases of a high clinical suspicion, influenza antigens were detected by means of immunochromatography for influenza A and B antigens in nasopharyngeal aspirates; finally, GRAM and sputum cultures with direct seeding were also processed.
- MR-proADM was determined in plasma through a sandwich immunoassay using time-resolved amplified cryptate emission technology (TRACE) in a Kryptor, BRAHMS AG. Reference values were P_95_: 0.52 nmol/L, median: 0.39 nmol/L. Detection limit: 0.05 nmol/L. Reagents were supplied by ThermoScientific (BRAHMS Iberia S.L.).
- Procalcitonin (PCT) and N-terminal pro b-type natriuretic peptide (NT-proBNP) were measured in plasma by electrochemiluminescence immunoassays on a COBAS 8000 modular analyzer (Roche Diagnostics, Mannheim, Germany). PCT reference values were < 0.05 μg/L with a detection limit < 0.02 ng/mL. NT-proBNP reference values were < 300 ng/L with a detection limit < 5 pg/mL.
- C-reactive protein (CRP) was measured in serum or plasma by immunoturbidimetry with latex particles on a COBAS 8000 modular analyzer (Roche Diagnostics, Mannheim, Germany). Reference values were < 0.5 mg/dL with a detection limit < 0.03 mg/dL.
- Other variables related to CAP worsening such as PSI, calculated according to Fine et al. [14], presence of bacteremia (significant isolation of a non-contaminant microorganism in hemocultures) or hospital admission on a common ground were also recorded.

### Statistical analysis

Categorical variables were expressed as percentages and continuous variables as medians with interquartile ranges. The relationship between MR-proADM and risk categories of PSI was assessed through the Jonckheere-Terpstra trend test. The correlation coefficient was calculated with the Kendall’s tau-b.

The association between continuous independent variables (analytical markers) and the main dependent variable was tested by the Student *t* test or the non-parametric Mann-Whitney *U* test when appropriate. For dichotomous categorical variables, the association was assessed using the [χ^2^] test or the Fisher exact test when categories had a size < 5. The association between independent variables and mortality at 90 days was analysed with a Cox regression survival analysis for independent continuous variables, and a Kaplan-Meyer survival curve with log-rank tests for independent categorical variables.

The predictive ability of continuous independent variables (those not included in the PSI that showed statistically significant probabilities in the univariate analysis) for either an adverse event or mortality at 90 days was evaluated through ROC (receiver operating characteristic) curves. The area under the curve (AUC) and its confidence interval was compared with that obtained with the PSI. Multivariate analyses to check for the best predictive model were done with binary logistic regression (with all possible equations) for adverse event, and with Cox regression for mortality at 90 days. The analyses included the PSI and those statistically significant independent variables not included in the PSI with an AUC > 0.70. Indices of goodness of fit were calculated choosing the best models according to the lowest AIC (Akaike Information Criterion). Odds Ratios (ORs) for binary logistic regression, hazard ratios (HRs) for Cox regression and 95% confidence intervals (CI95%) were also calculated. Models were calibrated with the Hosmer-Lemeshow test.

The predictive ability of MR-proADM combined with PSI was studied by means of a logistic regression adjust model. The obtained probabilities were analysed with ROC curves and compared with the predictive ability of MR-proADM and PSI models alone. A net reclassification index (NRI) was calculated to check whether the combined model of MR-proADM and PSI improved patients’ risk classification as compared with the model with PSI alone [25]. The risk limits for reclassification were assessed using the ones proposed by Schuetz et al.[19] that consider as low risk the probability of an advent < 5%, high risk > 20%, and two more categories for intermediate risk. For those cases with an adverse event, it was considered an improvement of risk category when individuals moved to a higher category when MR-proADM was added, and a worsening of risk category when individuals moved to a lower category. The inverse was considered for cases without adverse event. For mortality at 90 days, the risk limits for reclassification proposed by Courtais et al. [26] were used.

A p value of 0.05 was considered statistically significant. According to previous studies, it was estimated that, to predict mortality, a specificity of 80% could be achieved for an optimal cut-off of MR-proADM. With a confidence level of 95%, 246 patients would be needed to estimate this specificity with a precision of 5%. Data were introduced in Microsoft Office Access 2007 using a form sheet with restriction for anomalous data. Data were analysed using STATA, version 12 for Windows (StataCorp).

## Results

During the study period, a total of 60,021 medical emergencies (excluding pediatric and gynecological emergencies) were registered at the ED of the Gregorio Marañón University Hospital. This means an average of 398 emergencies per day. Five hundred and thirty-two patients were diagnosed with pneumonia (symptoms or signs associated with a new radiological infiltrate). Of these, 269 patients were diagnosed with CAP in the ED and did not meet any exclusion criteria, in 35 patients the diagnosis was not confirmed at discharge, and 8 patients were lost during the 3 months of follow-up. Thus, a total of 226 patients were included in the NACURG cohort analysed in this study (Fig 1).

Median age (IQR) was 75.6 (27.8) years. Of all patients, 55.3% were male, 39.4% had PSI IV or V, 1.8% were admitted to the ICU, and 11.8% were readmitted during the first month after discharge. Mortality was 1.8% and 4.4% at 30 and 90 days, respectively. Microbiological isolation was achieved in 32 (17.5%) hospitalised patients. *Streptococcus pneumoniae* was the most frequently isolated microorganism (68.8%). Main baseline characteristics of patients are recorded in Table 1.

**Table 1:** Baseline characteristics of the NACURG cohort. Relationship between different independent variables and adverse event and 90-day mortality after consulting the Emergency Department.

CHARACTERISTICS	TOTALS	ADVERSE EVENT		p*	90-DAY MORTALITY		p*
		WITH	WITHOUT		WITH	WITHOUT	
Total cohort, count (%)	226 (100%)	33(14.6)	193(85.4)		10(4.4)	216(95.6)	
Age (years), median (IQR)	75.6(27.8)	80.9(13.9)	74.6(29.1)	0.03	87.1(5.5)	74.2(28.0)	0.00
Male, n (%)	125(55.3)	21(63.6)	104(53.9)	0.3	9(90)	116(53.7)	0.02
Charlson Index ≥3, n (%)	49(21.7)	14(42.4)	35(18.1)	0.00	6(60)	43(19.9)	0.00
Prior antibiotic treatment, n (%)	60(26.7)	8(24.2)	52(26.9)	0.73	1(10)	59(27.4)	0.23
**Clinical sings**							
Confusion, n° (%)	17(7.5)	8(24.2)	9(4.7)	0.00	3(30)	14(6.5)	0.00
Respiratory rate>30 bpm, n (%)	10(4.4)	4(12.1)	6(3.1)	0.02	3(30)	7(3.2)	0.00
**Radiological findings**							
Extension, n° (%)				0.16			0.20
Unilobar	173(76.6)	22(66.7)	151(78.2)		6(60)	167(77.3)	
Multilobar	53(23.4)	11(33.3)	42(21.8)		4(40)	49(22.7)	
Effusion on chest X-ray, n (%)	20(8.9)	6(18.2)	14(7.3)	0.04	2(20)	18(8.3)	0.21
**Analytical variables**							
Urea(mg/dl), median (IQR)	38(28)	47(43)	36(26)	0.01	69(94)	36(27)	0.00
Sodium (mmol/L), median (IQR)	137(5)	136.5(9.5)	137(5)	0.40	137(7)	137(5)	0.11
Arterial O2 pressure (mmHg), median (IQR)	62(14)	61(16)	62(13)	0.83	58(21)	62(14)	0.85
pH, median (IQR)	7.44(0.07)	7.40(0.15)	7.44(0.07)	0.03	7.34(0.15)	7.44(0.07)	0.01
Lactate** (mmol/L), median (IQR)	1.5(1)*	2(1.6)	1.4(0.9)	0.01	1.7(3)	1.5(1)	0.00
Leucocytes (cells/microL), median (IQR)	12200(8000)	14400(8000)	11900(7700)	0.08	15050(6800)	12100(7800)	0.29
CRP(mg/dl), median (P_25_-P_75_)	9.15(15.7)	9.1(14)	9.4(16.1)	0.77	6.75(18.9)	9.25(15.2)	0.66
MR-proADM (nmol/L), median (IQR)	1.08(0.8)	1.56(1.37)	1.05(0.77)	0.00	3.15(2.47)	1.06(0.79)	0.00
PCT (μg/L), median (IQR)	0.13(0.47)	0.22(1.43)	0.12(0.37)	0.02	0.37(2.36)	0.13(0.43)	0.54
NT-proBNP(ng/L), median (IQR)	510(1518)	1621(3231)	384(1236)	0.00	2572(6130)	460(1427)	0.03
**CAP severity**							
PSI, median (IQR)	83.5(49)	81(54–100)	122(89–148)	0.00	145(45)	82(47.5)	0.00
PSI, n (%)				0.00			0.00
I,II,III(Mild)	137(60.6)	10(30.3)	127(65.8)		0(0)	137(63.4)	
VI(Moderate)	58(25.7)	9(27.3)	49(25.4)		3(30)	55(25.5)	
V(Severe)	31(13.7)	14(42.4)	17(8.8)		7(70)	24(11.1)	
Bacteremia, n (%)	6(2.7)	2(6.1)	4(2.1)	0.21	0(0)	6(2.8)	0.61
Hospital admission, n (%)	187(81)	28(84.4)	155(80.3)	0.53	10 (100)	0(0)	0.12
**Outcome**							
ICU admission, n (%)	4(1,8)						
30-day readmission, n (%)	26(11,8)***						
30-day mortality, n (%)	4(1,8)						

Differences between patients who died and those who survived were assessed by Cox regression survival analysis for independent continuous variables, and a Kaplan-Meyer survival curve with log-rank tests for independent categorical variables. Differences between patients with or without adverse event were assessed by the Student t test or the non-parametric Mann-Whitney U test for continuous variables and the [χ^2^] test or the Fisher exact test for dichotomous categorical variables.

*p: degree of statistical significance.

**Lactate levels only available for 122 patients (54%) and not therefore included in the multivariate analysis.

***The percentage of readmissions out of the total number of patients discharged (221; 4 patients died while in hospital and 1 was still inpatient at 30 days).

**Fig 2:**
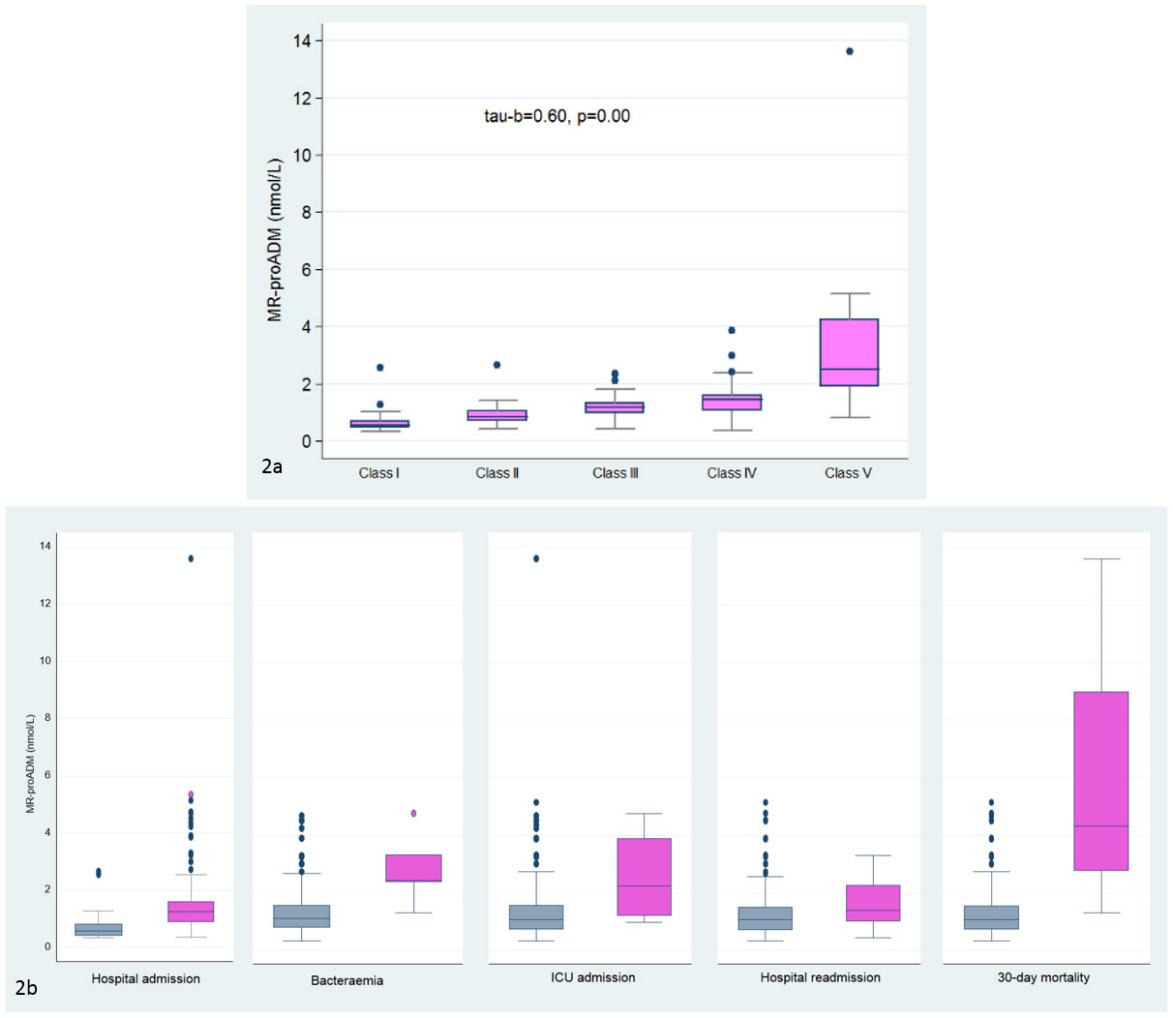
MR-proADM and CAP severity. Fig 2a. Relationship between MR-proADM and severity as established by the PSI. Analysis performed with the Jonckheere-Terpstra trend test. Tau b: Kendall’s rank correlation. p: level of statistical significance. Fig 2b. MR-proADM levels according to hospital admission, bacteremia, ICU admission, hospital readmission and 30-day mortality.

We found a significant increasing trend between MR-proADM levels and severity of CAP as assessed by PSI (Fig 2a). Likewise, MR-proADM levels were significantly higher in hospitalised patients (median [IQR]: 1.21[0.71] vs 0.58[0.38], p = 0.00), patients with bacteremia (2.42[0.93] vs 1.11[0.75], p = 0.00), patients with early readmission after discharge (1.39[1.23] vs 1.05[0.77], p = 0.00) and patients with early mortality (4.3[6.21] vs 1.07[0.8], p = 0.01). MR-proADM was predictor of short-term mortality with values similar to PSI (AUC 0.90[0.72–1.00] vs AUC 0.90[0.80–1.00], p = 0.91), respectively. A cut-off point for ProADM of 1.5 nmol/L had a sensitivity of 75%, specificity of 73%, likelihood ratio for positive test (LR+) of 2.73 and likelihood ratio for negative test (LR-) of 0.34 for predicting 30-day mortality. In patients admitted to the ICU, MR-proADM levels were higher (2.21 [2.67] vs 1.07[0.81]) but failed to reach statistical significance (Fig 2b).

### Main outcome: MR-proADM levels and adverse event

Thirty-three patients, 14.6% of the cohort, showed at least one adverse event (ICU admission, readmission after discharge, or death at 30 days of diagnosis). Independent variables significantly related to the combined variable adverse event and their statistical significances are recorded in Table 1.

**Fig 3:**
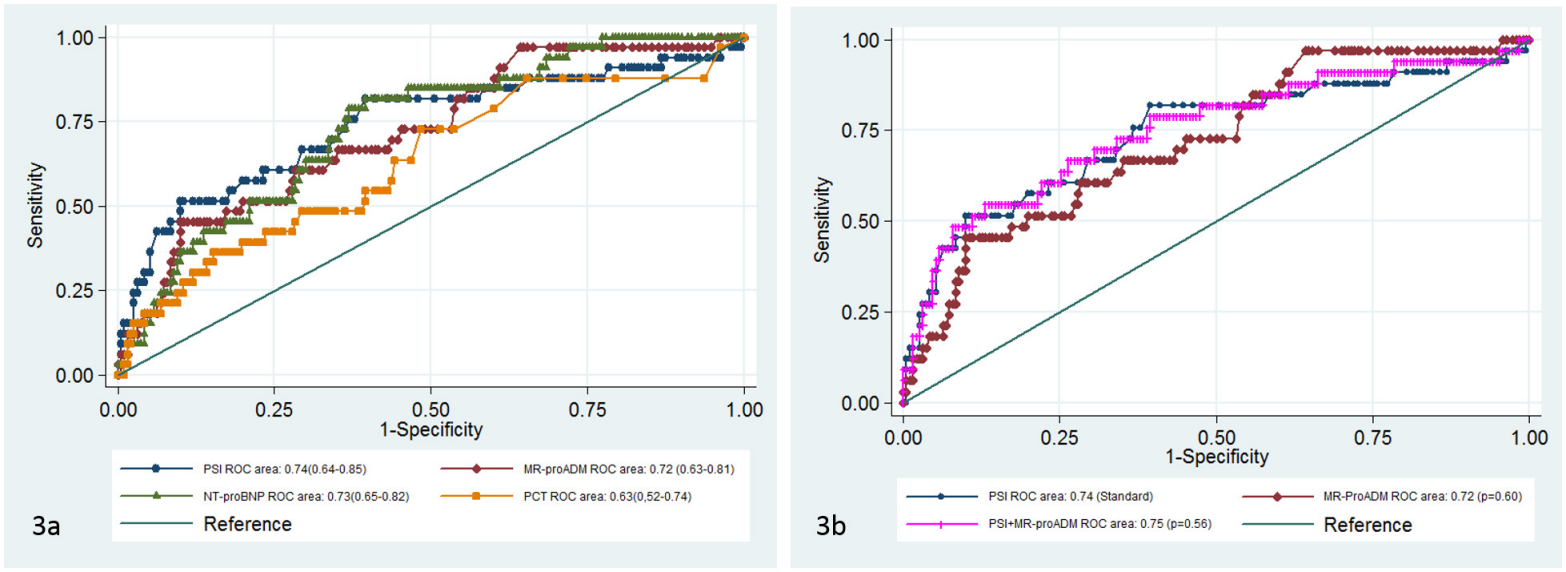
ROC curves for predicting adverse event. Fig 3a. ROC curves for different biomarkers and PSI. Fig 3b. ROC curves for the PSI & MR-proADM prediction model compared to PSI

When a ROC analysis to predict the probability of an adverse event was conducted, sensitivity was assessed by means of those patients that showed any adverse event during the follow-up (n = 33), and specificity by those patients that failed to show any adverse event at the 30 days after follow-up. All the statistically significant independent variables were added to the analysis except lactate that was available only in 122 patients. Although the highest AUC was found for PSI (0.74 [0.64–0.85]), this was not significantly higher than the AUC obtained for MR-proADM, NT-proBNP and PCT (p > 0.05) (Fig 3a). The optimal cut-off point for MR-proADM for predicting adverse event was 0.85 nmol/L, with 97% sensitivity, 36% specificity, likelihood ratio for positive test (LR+) 1.5 and likelihood ratio for negative test (LR-) 0.08. A cut-off point of 1.3 nmol/L had a sensitivity and a specificity of 64 and 65%, respectively.

Table 2 shows the different predictive models of adverse event together with several parameters of goodness to fit and calibration.

**Table 2:** Multivariate predictive models of adverse event and 90-day mortality.

ADVERSE EVENT						
	OR	CI 95%	p value	AIC	McFadden´s R^2^	Calibration χ2 (p value)
Model 1 (MaxM)			0.00	170.2	0.14	240.9(0.17)
PSI	1.02	1.01–1.03				
MR-proADM	1.16	0.77–1.75				
NT-proBNP	1.00	0.99–1.00				
Intercept	0.02	0.01–0.07				
Model 2			0.00	167.4	0.12	172.6(0.00)
PSI	1.02	1.01–1.03				
Intercept	0.02	0.01–0.06				
Model 3			0.00	168.5	0.17	240.1(0.17)
PSI	1.02	1.01–1.03				
MR-proADM	1.16	0.79–1.72				
Intercept	0.02	0.01–0.07				
**90-DAY MORTALITY**						
	HR	CI 95%	p value	AIC	Atkinson R^2^	Test of proportional-hazards assumption χ2(p value)
Model 1 (MaxM)			0.00	84.3	0.22	2.56(0.47)
PSI	1.00	0.97–1.02				
MR-proADM	3.14	1.25–7.86				
NT-proBNP	1.00	0.99–1.01				
Model 2			0.00	80.4	0.26	0.34(0.56)
MR-proADM	2.70	1.79–4.05				
Model 3			0.00	82.3	0.24	2.67(0.26)
PSI	1.00	0.97–1.02				
MR-proADM	2.93	1.23–6.97				
Model 4			0.00	94.0	0.13	0.07(0.80)
PSI	1.03	1.01–1.03				

MaxM: Maximum Model: includes significant independent variables in the univariate analysis with AUC higher than 0.7.

OR: Odds Ratio and HR: Hazard Ratio.

CI 95%: confidence interval of 95%.

p: level of statistical significance.

AIC: Akaike Information Criterion (better fit of the model when AIC lower).

McFadden´s and Atkinson R2: proportion of uncertainty data explained by the model.

Calibration χ2 (p value): Hosmer and Lemeshow test.

The combination of PSI and MR-proADM was the well-calibrated predictive model with a lower AIC, as well as the one that best explained data uncertainty (McFadden´s R2 = 0.17), but failed to improve the predictive potential of PSI alone (AUC 0.75 [0.65–0.85, p = 0.56]) (Fig 3b).

Results of reclassification tables are shown in Table 3. Ten patients were correctly classified when the combined PSI and MR-proADM model was used as compared with the model of PSI alone. Net reclassification improvement (NRI) index was statistically significant (7.69%, p = 0.03) with an improvement percentage of 3.03% (p = 0.32) for adverse event, and 4.66% (P = 0.02) for no adverse-event.

**Table 3:** Reclassification table for adverse event in PSI plus MR-proADM compared to PSI alone.

Total cohort, N = 226									
Adverse Event	PSI+MR-proADM						Reclassified		
PSI	<5%	5–10%	10–20%	>20%	Total		Increased risk	Decreased risk	NET correctly reclassified
Patients with adverse event (n = 33)									
<5%	2	1	0	0	3				
5–10%	0	3	0	0	3				
10–20%	0	0	10	0	10		1(3.03%)	0(0%)	1(3.03%)
>20%	0	0	0	17	17				
TOTAL	2	4	10	17	33				
Patients without adverse event (n = 193)									
<5%	29	3	0	0	32				
5–10%	0	59	0	0	59				
10–20%	0	5	67	0	72		3(1.55%)	12(6.22%)	9(4.66%)
>20%	0	0	7	23	30				
TOTAL	29	67	74	23	193				
						Total	4	12	10

### Secondary outcome: MR-proADM levels and mid-term mortality

**Fig 4:**
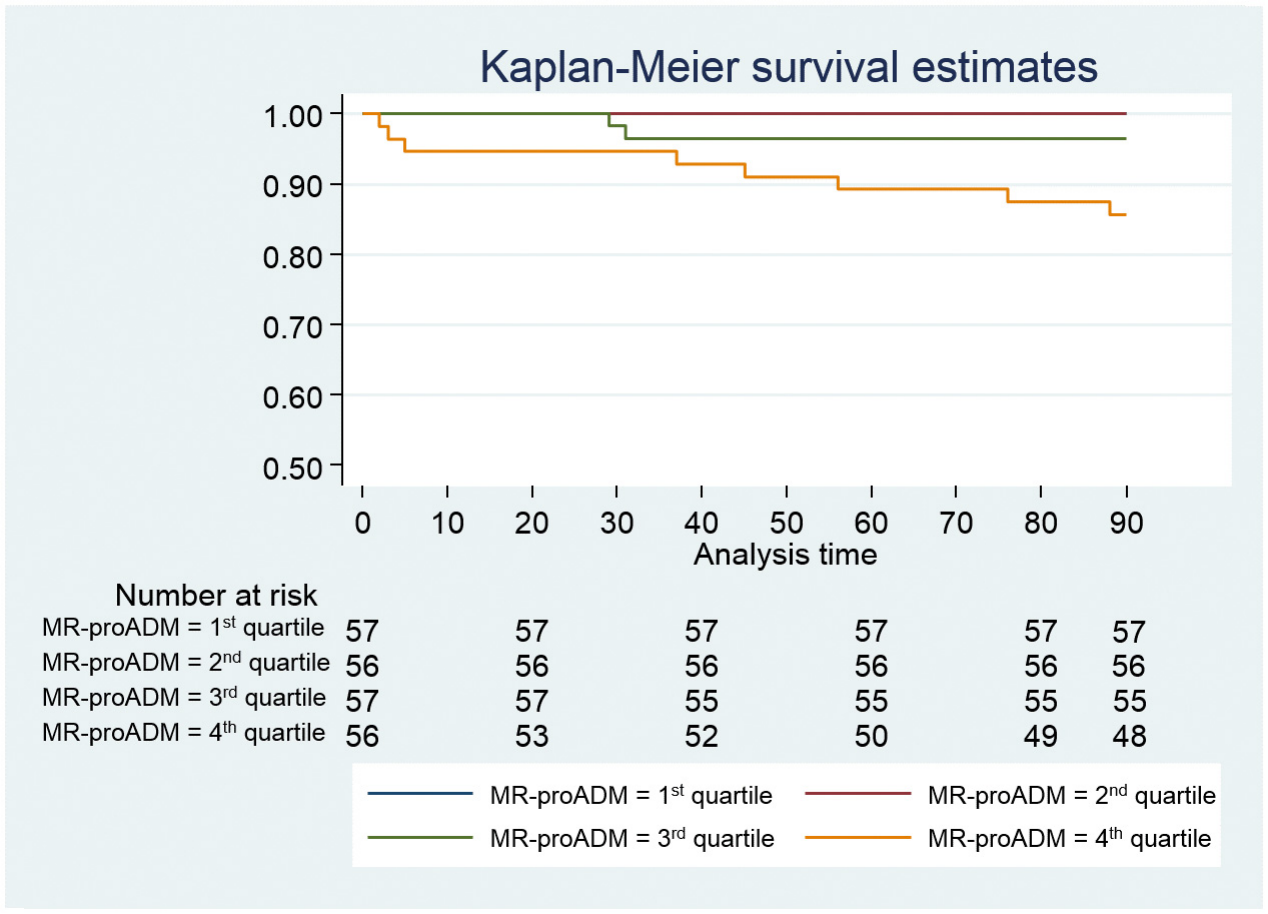
Kaplan-Meier survival curves at 90-days mortality according to MR-proADM quartiles.

Ten patients of the NACURG cohort died within 90 days after diagnosis. Variables associated with mid-term mortality are recorded in Table 1. MR-proADM levels were associated with mortality at 90 days (OR 2.70, 1.79–4.05) (Table 2). Significant differences (p < 0.01) were found among the survival curves according to MR-proADM quartiles (Fig 4).

**Fig 5:**
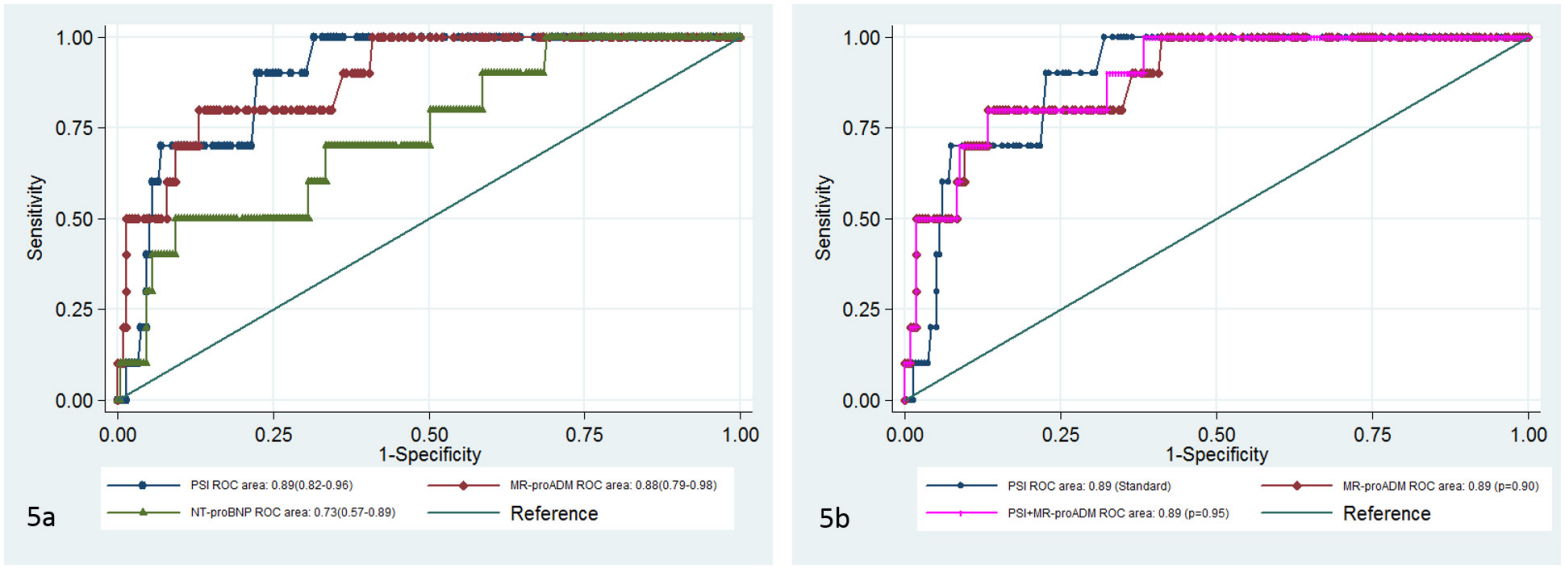
ROC curves for predicting 90-day mortality. Fig 5a. ROC curves for different biomarkers and PSI. Fig 5b. ROC curves for the PSI & MR-proADM prediction model compared to PSI.

MR-proADM was the analytical marker not included in the PSI with the highest AUC to predict mortality at 90 days (0.88 [0.79–0.98], showing an AUC similar to that obtained with the PSI (p = 0.90). ROC curve analysis to predict mortality at 90 days is depicted in (Fig 5a). A cut-off point for MR-proADM of 1.5 nmol/L had a sensitivity of 80%, specificity of 74%, likelihood ratio for positive test (LR+) of 3.09 and likelihood ratio for negative test (LR-) of 0.27 for predicting mid-term mortality. Both the MR-proADM alone and the combined PSI plus MR-proADM were the predictive models that showed the best adjustment (Table 2), but they did not improve the predictive ability of PSI alone (AUC 0.89 [0.80–0.98], p = 0.95) (Fig 5b).

The combined model failed to significantly improve classification of patients to predict mid-term risk mortality, NRI 5.74% (p = 0.83). The improvement percentage was -10% (p = 0.71) for event, and 15.74% (p = 0.00) for no event.

These and other supplementary results are depicted in (S1 File).

## Discussion

We found that MR-proADM, measured in the ED, is useful for predicting poor clinical outcome of CAP patients, showing a similar predictive ability than that found for PSI. Even though MR-proADM combined with PSI did not improve prognostic accuracy, it allowed a better reclassification of patients, mostly at the expense of a better classification of patients without adverse event.

Adrenomedullin (ADM) is a peptide produced by multiple tissues under stress conditions with vasodilatory, immunomodulatory, metabolic and bactericide attributed properties. MR-proADM is a more stable and easy-to-measure fragment that directly reflects ADM activity levels [27, 28]. Since 2006, several research groups have studied the prognostic role of MR-proADM in CAP patients finding a strong predictive potential of this peptide for short-term mortality [19, 21–23, 26, 29, 30]. Nonetheless, the decisions made in the ED cannot rely solely on the risk of mortality, especially in a sample of low mortality as ours which excludes cases with worse prognosis (Fig 1). It is also useful to know which patients are at increased risk of ICU admission or at early readmission after discharge. Certainly, an increased surveillance of these patients may improve survival.

Half of the patients at low risk of mortality according to PSI are hospitalised [31]. The combined method of PSI plus MR-proADM increases the number of patients without complications that are classified as at “low risk”. This can be useful in the clinical practice to decrease the number of unnecessary hospitalisations, avoiding nosocomial infections and reducing health costs [32].

Other studies had assessed the influence of MR-proADM in combined variables such as ours, although none had included early hospital readmission as a variable to predict poor outcome of CAP. Christ-Crain et al. [23] found that MR-proADM has a predictive ability similar to that of PSI, and that PSI combined with MR-proADM improves the predictive power of therapeutic failure defined as persistence or recurrence of clinical, analytical or radiologic alterations, or mortality during the follow-up (6.9 ± 1.9 weeks). Schuetz et al. [19]analysed the ProHOSP cohort [33] and concluded that the combined model of PSI improves the predictive potential of adverse event (empyema, abscess or acute respiratory distress syndrome, ICU admission or mortality at 30 days) as compared with PSI alone. Bello et al. [21] found that MR-proADM is a predictor of CAP complications (respiratory, renal and cardiac insufficiency among others) similar to PSI, but they did not examine the predictive power of the combined model. Other authors who also analysed the ProHOSP cohort concluded that MR-proADM plus CURB65 improves the predictive ability of adverse event with regard to CURB65 alone [20]. Additionally they found that combining MR-proADM with the REA-ICU score (Risk of Early Admission of ICU) improved the ability of predicting early severe community-acquired pneumonia (need of mechanical ventilation, vasoactive drugs or mortality at 30 days) in comparison with the REA-ICU score alone[31]. However, these studies did not analyse the prognostic usefulness of PSI.

As in the studies mentioned above, we found that MR-proADM is a good predictor of poor outcome of CAP. However, in our cohort we have not included patients *a priori* not eligible for hospital discharge. These patients were those needing specific antibiotic therapy (immunosuppressed or institutionalized) or those associated with worse CAP evolution such as patients with non-invasive home mechanical ventilation. Therefore, our cohort has a higher representation of patients at lower risk of mortality, the ones that most benefit from the determination of MR-proADM to decide whether they can receive outpatient treatment. These specific features of our cohort could be the reason for differences in optimal cut-off points compared to other studies.

A single clinical trial studied the feasibility and effects of adding MR-proADM to the CURB-65 score on triage decisions and length of stay [34]. There were no differences in adverse outcome or readmission but important logistic obstacles were found in this study. Moreover, CURB-65 score does not account for patients’ comorbidity, which may influence the CAP outcome. Our study hints at the possibility of designing clinical trials that evaluate the ability of new biomarkers, namely MR-proADM, to classify patients’ risks also accounting for additional clinically relevant endpoints beyond mortality, such as our study points to (i.e., ICU admission or short-term hospital readmission). MR-proADM could better inform clinical-making decisions in patients showing intermediate risk who are currently being admitted, or alternatively being discharged due to low specific mortality risk, albeit with a higher probability of short-term readmission.

We found that MR-proADM levels predict mid-term mortality as in previous studies [21, 29] but the combined model of PSI plus MR-proADM does not improve either the predictive potential or the reclassification of patients according to mid-term mortality risk. Our results are similar to that obtained by Huang et al.[29] that analysed a cohort with 60% of CAP patients with low risk (similar to our cohort) but differ from the ones reported by Bello et al. [21].

We would like to underline that, although NT-proBNP is also a predictor of poor outcome of CAP and mid-term mortality, it is not better than MR-proADM or PSI. We cannot discard that the substitution of the variable "cardiac insufficiency" included in the PSI by NT-proBNP levels could improve the predictive potential of this score, but this analysis was not included among the objectives of this study.

Several limitations of our study should be taken into consideration. Firstly, this is a single-center study, so that its results can be conditioned by both our patients’ profile and the way we work in our hospital and therefore be less generalisable. The endpoint ICU admission is vulnerable to bias by ICU admission policy and highly dependent on individual physician decisions. Moreover, “do not intubate” patients were not excluded. Our endpoint adverse event was mainly driven by a quite high readmission rate; however, ICU admission and 30-day mortality rates were low. Thus, generalisability of the findings to other health care systems is reduced. In addition, the analyses performed to predict short and mid-term mortality should be interpreted with caution because of the low number of adverse events recorded. We have not compared MR-proADM in relation to lactate levels in their prognostic ability because lactate level measurements were not available in more than 100 patients. Although the physician responsible for taking care of the patient did not know the results of MR-proADM, he/she did know that the patient could be included in the study, being able to produce an inclusion bias. Finally, even though the microbiological investigation of hospitalised patients was protocolised, it was not systematic, a fact that can explain the ethiological underdiagnosis.

In conclusion, as compared with PSI alone, MR-proADM in combination with PSI may be helpful in individual risk stratification for short-term poor outcome of CAP patients, allowing a better reclassification of patients. Although MR-proADM is a good predictor of mid-term mortality, similar to PSI, the combined model does not improve the predictive ability, and does not allow a more accurate reclassification of patients according to their mortality risk.

## Supporting Information

S1 File Supplementary results. (PDF)
