# Supplementary material for: Usefulness of Midregional Proadrenomedullin to Predict Poor Outcome in Patients with Community Acquired Pneumonia
Source: PLoS One. 2015 Jun 1;10(6):e0125212. doi: 10.1371/journal.pone.0125212 (PMC4452655; doi:10.1371/journal.pone.0125212)
Supplement: S1 File — (PDF) [file pone.0125212.s001.pdf]

```
  ___  ____  ____  ____  ____(R)
 /__    /   ____/   /   ____/
___/   /   /___/   /   /___/
  Statistics/Data Analysis
```

```
  ___  ____  ____  ____  ____ (R)
 /__    /   ____/   /   ____/
___/   /   /___/   /   /___/   12.1   Copyright 1985-2011 StataCorp LP
  Statistics/Data Analysis            StataCorp
                                      4905 Lakeway Drive
     Special Edition                  College Station, Texas 77845 USA
                                      800-STATA-PC        http://www.stata.com
                                      979-696-4600        stata@stata.com
                                      979-696-4601 (fax)
```

```
  1 . doedit "C:\Users\susan_000\Documents\NACURG\NUEVO\Definitivo\STATA NUEVO\NACURGPLOSOne.do"

  2 . do "C:\Users\susan_000\Documents\NACURG\NUEVO\Definitivo\STATA NUEVO\NACURGPLOSOne.do"

  3 . clear

  4 . use "C:\Users\susan_000\Documents\NACURG\NUEVO\NACURGPLOSOne.dta", clear
    (Estudio NACURG)

  5 .
  6 . rename ( Edad Sexo  CPSI PSIGru UCI Reing) (Age Sex PSI PSIClass ICU Readm)

  7 . label variable Age "Age (years)"

  8 . label variable Sex "Sex"

  9 . label define dSex 0 "Female" 1 "Male", replace

 10 . label variable PSIClass "PSI Class"

 11 . label define dSíntomas 0 "No" 1 "Yes", replace

 12 . label define dPSI 0 "Class I (Mild)" 1 "Class II (Mild)" 2 "Class III (Mild)" 3 "Class IV (Mo
    > re)", replace

 13 . label define dPSI3 0 " Mild " 1 " Moderate " 2 " Severe " , replace

 14 . label variable ICU "ICU admission"

 15 . label variable Mort30 "30-Day Mortality"

 16 . label variable MortGlo "90-Day Mortality"

 17 . label variable Readm "Hospital readmission"

 18 . label variable Etio "Microbiological isolation"

 19 . label variable Destino "Treatment site"

 20 . label define dDestino 0 "Discharge" 1 "Admission", replace
```

```
21 . label variable Atbpre "Prior antibiotic treatment"

22 . label variable FRM30 "Respiratory rate > 30 bpm"

23 . label variable RxExt "Rx extension"

24 . label variable Derrame "Rx effusion"

25 . generate HCP = HC ==1

26 . replace HCP = . if HC ==.
   (51 real changes made, 51 to missing)

27 . label variable HCP "Bacteremia"

28 . label values HCP dSíntomas

29 . label variable EndPoint1 "Adverse event"

30 .
31 . *RESULTS
32 . **BASELINE CHARACTERISTICS
33 . summarize Age, detail

                         Age (years)
   -------------------------------------------------------------
         Percentiles      Smallest
    1%     20.53114       19.08282
    5%     30.05886       20.07392
   10%     37.96304       20.53114       Obs                 226
   25%     56.03012       20.59138       Sum of Wgt.         226

   50%     75.64545                      Mean           68.70341
                           Largest       Std. Dev.      19.48983
   75%     83.85216        94.6037
   90%      88.5065       96.68994       Variance       379.8536
   95%     89.97947       97.51129       Skewness      -.8270216
   99%     96.68994       100.5394       Kurtosis       2.685489

34 . tab1 Sex

   -> tabulation of Sex

           Sex |      Freq.     Percent        Cum.
   ------------+-----------------------------------
        Female |        101       44.69       44.69
          Male |        125       55.31      100.00
   ------------+-----------------------------------
         Total |        226      100.00

35 . tab1 PSIClass

   -> tabulation of PSIClass

             PSI Class |      Freq.     Percent        Cum.
   --------------------+-----------------------------------
      Class I (Mild) |         44       19.47       19.47
     Class II (Mild) |         40       17.70       37.17
    Class III (Mild) |         53       23.45       60.62
   Class IV (Moderate) |         58       25.66       86.28
    Class V (Severe) |         31       13.72      100.00
   --------------------+-----------------------------------
                 Total |        226      100.00
```

36 . tab1 ICU

-> tabulation of ICU

ICU admission	Freq.	Percent	Cum.
No	222	98.23	98.23
Yes	4	1.77	100.00
Total	226	100.00	

37 . tab1 Readm

-> tabulation of Readm

Hospital readmission	Freq.	Percent	Cum.
No	195	88.24	88.24
Yes	26	11.76	100.00
Total	221	100.00	

38 . tab1 Mort30

-> tabulation of Mort30

30-Day Mortality	Freq.	Percent	Cum.
No	222	98.23	98.23
Yes	4	1.77	100.00
Total	226	100.00	

39 . tab1 MortGlo

-> tabulation of MortGlo

90-Day Mortality	Freq.	Percent	Cum.
No	216	95.58	95.58
Yes	10	4.42	100.00
Total	226	100.00	

40 . tab1 Destino

-> tabulation of Destino

Treatment site	Freq.	Percent	Cum.
Discharge	43	19.03	19.03
Admission	183	80.97	100.00
Total	226	100.00	

41 . by Destino, sort : tabulate Etio if Etio !=0 & Etio!=9

-> Destino = Discharge

Microbiological isolation	Freq.	Percent	Cum.
Neumococo	1	50.00	50.00
Micoplasma pneumonia	1	50.00	100.00
Total	2	100.00	

-> Destino = Admission

Microbiological isolation	Freq.	Percent	Cum.
Neumococo	22	68.75	68.75
Pseudomona	1	3.13	71.88
Legionella	1	3.13	75.00
S. viridans	1	3.13	78.13
H. influenza	1	3.13	81.25
Influenza B	5	15.63	96.88
Aspergillus	1	3.13	100.00
Total	32	100.00	

42 . tab1 Etio

-> tabulation of Etio

Microbiological isolation	Freq.	Percent	Cum.
No aislamiento	180	79.65	79.65
Neumococo	23	10.18	89.82
Pseudomona	1	0.44	90.27
Legionella	1	0.44	90.71
S. viridans	1	0.44	91.15
H. influenza	1	0.44	91.59
Influenza B	5	2.21	93.81
Aspergillus	1	0.44	94.25
Contaminante	12	5.31	99.56
Micoplasma pneumonia	1	0.44	100.00
Total	226	100.00	

43 . tab1 CCharlson

-> tabulation of CCharlson

Charlson>=3	Freq.	Percent	Cum.
No	177	78.32	78.32
Yes	49	21.68	100.00
Total	226	100.00	

44 . tab1 Atbpre

-> tabulation of Atbpre

Prior antibiotic treatment	Freq.	Percent	Cum.
No	165	73.33	73.33
Yes	60	26.67	100.00
Total	225	100.00	

45 . tab1 Confu

-> tabulation of Confu

Confusión	Freq.	Percent	Cum.
No	209	92.48	92.48
Yes	17	7.52	100.00
Total	226	100.00	

46 . tab1 FRM30

-> tabulation of FRM30

Respiratory rate > 30 bpm	Freq.	Percent	Cum.
No	216	95.58	95.58
Yes	10	4.42	100.00
Total	226	100.00	

47 . tab1 RxExt

-> tabulation of RxExt

Rx extension	Freq.	Percent	Cum.
Unilobar	173	76.55	76.55
Multilobar	27	11.95	88.50
Bilateral	26	11.50	100.00
Total	226	100.00	

48 . tab1 Derrame

-> tabulation of Derrame

Rx effusion	Freq.	Percent	Cum.
No	206	91.15	91.15
Yes	20	8.85	100.00
Total	226	100.00	

```
49 . tabstat Age PSI Na pO2 pH Lactico Leucocitos, statistics( median iqr )
```

stats	Age	PSI	Na	pO2	pH	Lactico	Leucoc~s
p50	75.64545	83.5	137	62	7.44	1.5	12200
iqr	27.82204	49	5	14	.07	1	8000

```
50 . tabstat PCR ProADM PCT ProBNP, statistics( median iqr )
```

stats	PCR	ProADM	PCT	ProBNP
p50	9.15	1.075	.13	510
iqr	15.65	.8	.465	1518

```
51 . tab1 HCP
```

-> tabulation of HCP

Bacteremia	Freq.	Percent	Cum.
No	169	96.57	96.57
Yes	6	3.43	100.00
Total	175	100.00	

```
52 .
53 . by PSIClass, sort : tabstat ProADM, statistics( median iqr )
```

-> PSIClass = Class I (Mild)

variable	p50	iqr
ProADM	.555	.25

-> PSIClass = Class II (Mild)

variable	p50	iqr
ProADM	.845	.335

-> PSIClass = Class III (Mild)

variable	p50	iqr
ProADM	1.17	.37

-> PSIClass = Class IV (Moderate)

variable	p50	iqr
ProADM	1.435	.53

-> PSIClass = Class V (Severe)

variable	p50	iqr
ProADM	2.5	2.35

```
54 . ktau ProADM PSIClass

  Number of obs =     226
Kendall's tau-a =       0.5497
Kendall's tau-b =       0.6182
Kendall's score =   13976
    SE of score =    1109.531   (corrected for ties)

Test of Ho: ProADM and PSIClass are independent
     Prob > |z| =       0.0000  (continuity corrected)

55 . by Destino, sort : tabstat ProADM, statistics( median iqr )

-------------------------------------------------------------------------------
-> Destino = Discharge

    variable |       p50       iqr
-------------+--------------------
      ProADM |       .58       .38
----------------------------------

-------------------------------------------------------------------------------
-> Destino = Admission

    variable |       p50       iqr
-------------+--------------------
      ProADM |      1.21       .71
----------------------------------

56 . ranksum ProADM, by(Destino)

Two-sample Wilcoxon rank-sum (Mann-Whitney) test

     Destino |      obs    rank sum    expected
-------------+---------------------------------
   Discharge |       43        2012      4880.5
   Admission |      183       23639     20770.5
-------------+---------------------------------
    combined |      226       25651       25651

unadjusted variance   148855.25
adjustment for ties      -15.09
                     ----------
adjusted variance     148840.16

Ho: ProADM(Destino==Discharge) = ProADM(Destino==Admission)
             z =  -7.435
    Prob > |z| =   0.0000

57 . by HCP, sort : tabstat ProADM, statistics( median iqr )

-------------------------------------------------------------------------------
-> HCP = No

    variable |       p50       iqr
-------------+--------------------
      ProADM |      1.11       .75
----------------------------------

-------------------------------------------------------------------------------
-> HCP = Yes

    variable |       p50       iqr
-------------+--------------------
      ProADM |      2.42       .93
----------------------------------

-------------------------------------------------------------------------------
-> HCP = .
```

```
    variable |       p50       iqr
-------------+--------------------
      ProADM |       .89       .81
----------------------------------

58 . ranksum ProADM, by(HCP)

Two-sample Wilcoxon rank-sum (Mann-Whitney) test

         HCP |      obs    rank sum    expected
-------------+---------------------------------
          No |      169       14476       14872
         Yes |        6         924         528
-------------+---------------------------------
    combined |      175       15400       15400

unadjusted variance    14872.00
adjustment for ties       -1.85
                     ----------
adjusted variance      14870.15

Ho: ProADM(HCP==No) = ProADM(HCP==Yes)
             z =  -3.247
    Prob > |z| =   0.0012

59 . by Readm, sort : tabstat ProADM, statistics( median iqr )

-------------------------------------------------------------------------------
-> Readm = No

    variable |       p50       iqr
-------------+--------------------
      ProADM |      1.05       .77
----------------------------------

-------------------------------------------------------------------------------
-> Readm = Yes

    variable |       p50       iqr
-------------+--------------------
      ProADM |     1.385      1.23
----------------------------------

-------------------------------------------------------------------------------
-> Readm = .

    variable |       p50       iqr
-------------+--------------------
      ProADM |      4.36       .44
----------------------------------

60 . ranksum ProADM, by(Readm)

Two-sample Wilcoxon rank-sum (Mann-Whitney) test

       Readm |      obs    rank sum    expected
-------------+---------------------------------
          No |      195     20763.5       21645
         Yes |       26      3767.5        2886
-------------+---------------------------------
    combined |      221       24531       24531

unadjusted variance    93795.00
adjustment for ties      -10.11
                     ----------
adjusted variance      93784.89

Ho: ProADM(Readm==No) = ProADM(Readm==Yes)
             z =  -2.878
    Prob > |z| =   0.0040
```

```
61 . by Mort30, sort : tabstat ProADM, statistics( median iqr )

-> Mort30 = No

    variable |       p50       iqr
-------------+--------------------
      ProADM |      1.07        .8
----------------------------------

-> Mort30 = Yes

    variable |       p50       iqr
-------------+--------------------
      ProADM |       4.3      6.21
----------------------------------

62 . ranksum ProADM, by(Mort30)

Two-sample Wilcoxon rank-sum (Mann-Whitney) test

      Mort30 |      obs    rank sum    expected
-------------+---------------------------------
          No |      222       24845       25197
         Yes |        4         806         454
-------------+---------------------------------
    combined |      226       25651       25651

unadjusted variance    16798.00
adjustment for ties       -1.70
                     ----------
adjusted variance      16796.30

Ho: ProADM(Mort30==No) = ProADM(Mort30==Yes)
             z =  -2.716
    Prob > |z| =   0.0066

63 . by ICU, sort : tabstat ProADM, statistics( median iqr )

-> ICU = No

    variable |       p50       iqr
-------------+--------------------
      ProADM |      1.07       .81
----------------------------------

-> ICU = Yes

    variable |       p50       iqr
-------------+--------------------
      ProADM |      2.21      2.67
----------------------------------

64 . ranksum ProADM, by(ICU)

Two-sample Wilcoxon rank-sum (Mann-Whitney) test

         ICU |      obs    rank sum    expected
-------------+---------------------------------
          No |      222     24966.5       25197
         Yes |        4       684.5         454
-------------+---------------------------------
    combined |      226       25651       25651

unadjusted variance    16798.00
adjustment for ties       -1.70
                     ----------
adjusted variance      16796.30
```

```
   Ho: ProADM(ICU==No) = ProADM(ICU==Yes)
                z =  -1.779
    Prob > |z| =   0.0753

65 .
66 . roctab Mort30 ProADM, detail
```

Detailed report of sensitivity and specificity

Cutpoint	Sensitivity	Specificity	Correctly Classified	LR+	LR-
(>= .33)	100.00%	0.00%	1.77%	1.0000	
(>= .34)	100.00%	0.45%	2.21%	1.0045	0.0000
(>= .35)	100.00%	0.90%	2.65%	1.0091	0.0000
(>= .36)	100.00%	1.35%	3.10%	1.0137	0.0000
(>= .37)	100.00%	1.80%	3.54%	1.0183	0.0000
(>= .4)	100.00%	2.25%	3.98%	1.0230	0.0000
(>= .41)	100.00%	3.15%	4.87%	1.0326	0.0000
(>= .42)	100.00%	3.60%	5.31%	1.0374	0.0000
(>= .43)	100.00%	4.95%	6.64%	1.0521	0.0000
(>= .44)	100.00%	5.86%	7.52%	1.0622	0.0000
(>= .45)	100.00%	6.76%	8.41%	1.0725	0.0000
(>= .48)	100.00%	7.66%	9.29%	1.0829	0.0000
(>= .49)	100.00%	8.11%	9.73%	1.0882	0.0000
(>= .51)	100.00%	8.56%	10.18%	1.0936	0.0000
(>= .52)	100.00%	9.46%	11.06%	1.1045	0.0000
(>= .53)	100.00%	10.36%	11.95%	1.1156	0.0000
(>= .55)	100.00%	11.26%	12.83%	1.1269	0.0000
(>= .56)	100.00%	12.16%	13.72%	1.1385	0.0000
(>= .57)	100.00%	13.06%	14.60%	1.1503	0.0000
(>= .58)	100.00%	13.96%	15.49%	1.1623	0.0000
(>= .61)	100.00%	14.86%	16.37%	1.1746	0.0000
(>= .62)	100.00%	16.22%	17.70%	1.1935	0.0000
(>= .63)	100.00%	17.57%	19.03%	1.2131	0.0000
(>= .64)	100.00%	18.47%	19.91%	1.2265	0.0000
(>= .66)	100.00%	18.92%	20.35%	1.2333	0.0000
(>= .67)	100.00%	19.82%	21.24%	1.2472	0.0000
(>= .68)	100.00%	20.27%	21.68%	1.2542	0.0000
(>= .7)	100.00%	21.62%	23.01%	1.2759	0.0000
(>= .71)	100.00%	22.97%	24.34%	1.2982	0.0000
(>= .72)	100.00%	23.87%	25.22%	1.3136	0.0000
(>= .73)	100.00%	24.32%	25.66%	1.3214	0.0000
(>= .74)	100.00%	24.77%	26.11%	1.3293	0.0000
(>= .75)	100.00%	25.23%	26.55%	1.3373	0.0000
(>= .77)	100.00%	25.68%	26.99%	1.3455	0.0000
(>= .78)	100.00%	26.13%	27.43%	1.3537	0.0000
(>= .79)	100.00%	26.58%	27.88%	1.3620	0.0000
(>= .8)	100.00%	27.48%	28.76%	1.3789	0.0000
(>= .82)	100.00%	29.73%	30.97%	1.4231	0.0000
(>= .83)	100.00%	30.63%	31.86%	1.4416	0.0000
(>= .84)	100.00%	31.08%	32.30%	1.4510	0.0000
(>= .85)	100.00%	31.53%	32.74%	1.4605	0.0000
(>= .86)	100.00%	34.68%	35.84%	1.5310	0.0000
(>= .87)	100.00%	35.14%	36.28%	1.5417	0.0000
(>= .88)	100.00%	36.04%	37.17%	1.5634	0.0000
(>= .89)	100.00%	36.49%	37.61%	1.5745	0.0000
(>= .9)	100.00%	36.94%	38.05%	1.5857	0.0000
(>= .91)	100.00%	38.29%	39.38%	1.6204	0.0000
(>= .92)	100.00%	39.19%	40.27%	1.6444	0.0000
(>= .93)	100.00%	39.64%	40.71%	1.6567	0.0000
(>= .96)	100.00%	40.09%	41.15%	1.6692	0.0000
(>= .97)	100.00%	40.54%	41.59%	1.6818	0.0000
(>= .98)	100.00%	41.44%	42.48%	1.7077	0.0000
(>= .99)	100.00%	42.34%	43.36%	1.7344	0.0000
(>= 1.02)	100.00%	43.24%	44.25%	1.7619	0.0000
(>= 1.03)	100.00%	44.59%	45.58%	1.8049	0.0000
(>= 1.04)	100.00%	46.40%	47.35%	1.8655	0.0000
(>= 1.05)	100.00%	47.30%	48.23%	1.8974	0.0000
(>= 1.06)	100.00%	48.20%	49.12%	1.9304	0.0000
(>= 1.07)	100.00%	49.55%	50.44%	1.9821	0.0000
(>= 1.08)	100.00%	50.90%	51.77%	2.0367	0.0000

(>= 1.09)	100.00%	51.35%	52.21%	2.0556	0.0000
(>= 1.1)	100.00%	52.25%	53.10%	2.0943	0.0000
(>= 1.11)	100.00%	53.15%	53.98%	2.1346	0.0000
(>= 1.12)	100.00%	54.05%	54.87%	2.1765	0.0000
(>= 1.14)	100.00%	54.50%	55.31%	2.1980	0.0000
(>= 1.15)	100.00%	55.41%	56.19%	2.2424	0.0000
(>= 1.17)	100.00%	55.86%	56.64%	2.2653	0.0000
(>= 1.18)	100.00%	56.31%	57.08%	2.2887	0.0000
(>= 1.19)	100.00%	57.21%	57.96%	2.3368	0.0000
(>= 1.2)	100.00%	58.11%	58.85%	2.3871	0.0000
(>= 1.21)	100.00%	59.01%	59.73%	2.4396	0.0000
(>= 1.22)	100.00%	59.46%	60.18%	2.4667	0.0000
(>= 1.23)	100.00%	59.91%	60.62%	2.4944	0.0000
(>= 1.26)	100.00%	60.36%	61.06%	2.5227	0.0000
(>= 1.28)	100.00%	60.81%	61.50%	2.5517	0.0000
(>= 1.29)	100.00%	61.71%	62.39%	2.6118	0.0000
(>= 1.3)	100.00%	62.16%	62.83%	2.6429	0.0000
(>= 1.31)	75.00%	63.96%	64.16%	2.0812	0.3908
(>= 1.32)	75.00%	64.41%	64.60%	2.1076	0.3881
(>= 1.33)	75.00%	65.32%	65.49%	2.1623	0.3828
(>= 1.34)	75.00%	65.77%	65.93%	2.1908	0.3801
(>= 1.35)	75.00%	66.67%	66.81%	2.2500	0.3750
(>= 1.36)	75.00%	67.12%	67.26%	2.2808	0.3725
(>= 1.38)	75.00%	67.57%	67.70%	2.3125	0.3700
(>= 1.39)	75.00%	68.47%	68.58%	2.3786	0.3651
(>= 1.42)	75.00%	68.92%	69.03%	2.4130	0.3627
(>= 1.43)	75.00%	69.37%	69.47%	2.4485	0.3604
(>= 1.44)	75.00%	70.27%	70.35%	2.5227	0.3558
(>= 1.45)	75.00%	71.17%	71.24%	2.6016	0.3513
(>= 1.47)	75.00%	71.62%	71.68%	2.6429	0.3491
(>= 1.5)	75.00%	72.52%	72.57%	2.7295	0.3447
(>= 1.51)	75.00%	72.97%	73.01%	2.7750	0.3426
(>= 1.53)	75.00%	74.32%	74.34%	2.9211	0.3364
(>= 1.54)	75.00%	74.77%	74.78%	2.9732	0.3343
(>= 1.55)	75.00%	75.23%	75.22%	3.0273	0.3323
(>= 1.56)	75.00%	76.13%	76.11%	3.1415	0.3284
(>= 1.57)	75.00%	77.03%	76.99%	3.2647	0.3246
(>= 1.59)	75.00%	77.48%	77.43%	3.3300	0.3227
(>= 1.6)	75.00%	78.83%	78.76%	3.5426	0.3171
(>= 1.61)	75.00%	79.73%	79.65%	3.7000	0.3136
(>= 1.65)	75.00%	80.18%	80.09%	3.7841	0.3118
(>= 1.67)	75.00%	80.63%	80.53%	3.8721	0.3101
(>= 1.69)	75.00%	81.53%	81.42%	4.0610	0.3066
(>= 1.7)	75.00%	81.98%	81.86%	4.1625	0.3049
(>= 1.71)	75.00%	82.43%	82.30%	4.2692	0.3033
(>= 1.74)	75.00%	82.88%	82.74%	4.3816	0.3016
(>= 1.8)	75.00%	83.33%	83.19%	4.5000	0.3000
(>= 1.82)	75.00%	83.78%	83.63%	4.6250	0.2984
(>= 1.89)	75.00%	84.68%	84.51%	4.8971	0.2952
(>= 1.97)	75.00%	85.14%	84.96%	5.0455	0.2937
(>= 2.03)	75.00%	85.59%	85.40%	5.2031	0.2921
(>= 2.06)	75.00%	86.04%	85.84%	5.3710	0.2906
(>= 2.12)	75.00%	86.49%	86.28%	5.5500	0.2891
(>= 2.19)	75.00%	86.94%	86.73%	5.7414	0.2876
(>= 2.22)	75.00%	87.39%	87.17%	5.9464	0.2861
(>= 2.25)	75.00%	87.84%	87.61%	6.1667	0.2846
(>= 2.27)	75.00%	88.29%	88.05%	6.4038	0.2832
(>= 2.29)	75.00%	88.74%	88.50%	6.6600	0.2817
(>= 2.31)	75.00%	89.19%	88.94%	6.9375	0.2803
(>= 2.35)	75.00%	89.64%	89.38%	7.2391	0.2789
(>= 2.37)	75.00%	90.09%	89.82%	7.5682	0.2775
(>= 2.39)	75.00%	90.54%	90.27%	7.9286	0.2761
(>= 2.42)	75.00%	90.99%	90.71%	8.3250	0.2748
(>= 2.44)	75.00%	91.89%	91.59%	9.2500	0.2721
(>= 2.5)	75.00%	92.34%	92.04%	9.7941	0.2707
(>= 2.55)	75.00%	92.79%	92.48%	10.4063	0.2694
(>= 2.66)	75.00%	93.69%	93.36%	11.8929	0.2668
(>= 2.72)	75.00%	94.14%	93.81%	12.8077	0.2656
(>= 2.99)	75.00%	94.59%	94.25%	13.8750	0.2643
(>= 3.23)	75.00%	95.50%	95.13%	16.6500	0.2618
(>= 3.29)	75.00%	95.95%	95.58%	18.5000	0.2606
(>= 3.3)	75.00%	96.40%	96.02%	20.8125	0.2593

(>= 3.86)	75.00%	96.85%	96.46%	23.7857	0.2581
(>= 3.9)	75.00%	97.30%	96.90%	27.7501	0.2569
(>= 4.24)	75.00%	97.75%	97.35%	33.3000	0.2558
(>= 4.36)	50.00%	97.75%	96.90%	22.2000	0.5115
(>= 4.5)	25.00%	97.75%	96.46%	11.1000	0.7673
(>= 4.52)	25.00%	98.20%	96.90%	13.8750	0.7638
(>= 4.68)	25.00%	98.65%	97.35%	18.5000	0.7603
(>= 4.75)	25.00%	99.10%	97.79%	27.7500	0.7568
(>= 5.14)	25.00%	99.55%	98.23%	55.5004	0.7534
(>= 13.6)	25.00%	100.00%	98.67%		0.7500
(> 13.6)	0.00%	100.00%	98.23%		1.0000

Obs	ROC Area	Std. Err.	—Asymptotic Normal— [95% Conf.	Interval]
226	0.8964	0.0893	0.72129	1.00000

67 . roctab Mort30 PSI

Obs	ROC Area	Std. Err.	—Asymptotic Normal— [95% Conf.	Interval]
226	0.9020	0.0506	0.80281	1.00000

68 . roccomp Mort30 ProADM PSI

	Obs	ROC Area	Std. Err.	—Asymptotic Normal— [95% Conf.	Interval]
**ProADM**	226	0.8964	0.0893	0.72129	1.00000
**PSI**	226	0.9020	0.0506	0.80281	1.00000

Ho: area(**ProADM**) = area(**PSI**)
chi2(**1**) = **0.02** Prob>chi2 = **0.9008**

69 .
70 . **MAIN OUTCOME: MR-PROADM AND ADVERSE EVENT
71 . tab1 EndPoint1

-> tabulation of EndPoint1

Adverse event	Freq.	Percent	Cum.
No	193	85.40	85.40
Yes	33	14.60	100.00
Total	226	100.00	

72 . by EndPoint1, sort : swilk PSI Age Charlson ProADM PCT ProBNP PCR Lactico Urea Na Leucocito

-> EndPoint1 = No

Shapiro-Wilk W test for normal data

Variable	Obs	W	V	z	Prob>z
PSI	193	0.96896	4.489	3.449	0.00028
Age	193	0.92165	11.332	5.576	0.00000
Charlson	193	0.91062	12.928	5.878	0.00000
ProADM	193	0.78011	31.803	7.946	0.00000
PCT	191	0.32387	96.910	10.499	0.00000
ProBNP	192	0.49053	73.354	9.862	0.00000
PCR	191	0.89242	15.419	6.280	0.00000
Lactico	99	0.86145	11.343	5.385	0.00000
Urea	193	0.84571	22.316	7.132	0.00000
Na	193	0.93305	9.683	5.214	0.00000
Leucocitos	193	0.92865	10.320	5.361	0.00000
pO2	117	0.98226	1.671	1.149	0.12537

pH	165	0.93885	7.722	4.657	0.00000

-> EndPoint1 = Yes

Shapiro-Wilk W test for normal data

Variable	Obs	W	V	z	Prob>z
PSI	33	0.98467	0.523	-1.347	0.91093
Age	33	0.80587	6.628	3.934	0.00004
Charlson	33	0.91143	3.024	2.301	0.01068
ProADM	33	0.56163	14.965	5.628	0.00000
PCT	33	0.59107	13.960	5.483	0.00000
ProBNP	33	0.55932	15.044	5.639	0.00000
PCR	33	0.85627	4.907	3.308	0.00047
Lactico	23	0.79566	5.345	3.408	0.00033
Urea	33	0.86652	4.557	3.154	0.00080
Na	32	0.88681	3.776	2.758	0.00291
Leucocitos	33	0.95930	1.389	0.684	0.24698
pO2	25	0.93964	1.677	1.057	0.14519
pH	30	0.94721	1.678	1.070	0.14226

73 . by EndPoint1, sort : tabstat Age, statistics(median iqr)

-> EndPoint1 = No

variable	p50	iqr
Age	74.63655	29.07597

-> EndPoint1 = Yes

variable	p50	iqr
Age	80.92539	13.93292

74 . ranksum Age, by(EndPoint1)

Two-sample Wilcoxon rank-sum (Mann-Whitney) test

EndPoint1	obs	rank sum	expected
No	193	21170	21905.5
Yes	33	4481	3745.5
combined	226	25651	25651

```
unadjusted variance   120480.25
adjustment for ties       -0.06
                      ---------
adjusted variance     120480.19

Ho: Age(EndPoi~1==No) = Age(EndPoi~1==Yes)
             z =  -2.119
    Prob > |z| =   0.0341
```

75 . cs EndPoint1 Sex, or

	Sex Exposed	Unexposed	Total		
Cases	21	12	33		
Noncases	104	89	193		
Total	125	101	226		
Risk	.168	.1188119	.1460177		
	Point estimate		[95% Conf. Interval]		
Risk difference	.0491881		-.041793	.1401693	
Risk ratio	1.414		.7315583	2.733065	
Attr. frac. ex.	.2927864		-.3669451	.6341104	
Attr. frac. pop	.1863186				
Odds ratio	1.497596		.7057624	3.173132	(Cornfield)

chi2(1) = 1.08 Pr>chi2 = 0.2978

76 . cs EndPoint1 CCharlson, or

	Charlson>=3 Exposed	Unexposed	Total		
Cases	14	19	33		
Noncases	35	158	193		
Total	49	177	226		
Risk	.2857143	.1073446	.1460177		
	Point estimate		[95% Conf. Interval]		
Risk difference	.1783697		.0439113	.312828	
Risk ratio	2.661654		1.441054	4.916125	
Attr. frac. ex.	.6242938		.3060636	.7965878	
Attr. frac. pop	.2648519				
Odds ratio	3.326316		1.539685	7.20006	(Cornfield)

chi2(1) = 9.79 Pr>chi2 = 0.0018

77 . cs EndPoint1 Atbpre, or

	Prior antibiotic treatment Exposed	Unexposed	Total		
Cases	8	25	33		
Noncases	52	140	192		
Total	60	165	225		
Risk	.1333333	.1515152	.1466667		
	Point estimate		[95% Conf. Interval]		
Risk difference	-.0181818		-.1201201	.0837564	
Risk ratio	.88		.4201627	1.843096	
Prev. frac. ex.	.12		-.8430956	.5798373	
Prev. frac. pop	.032				
Odds ratio	.8615385		.3728989	1.997737	(Cornfield)

chi2(1) = 0.12 Pr>chi2 = 0.7332

78 . cs EndPoint1 Confu, or

	Confusión Exposed	Unexposed	Total	
Cases	8	25	33	
Noncases	9	184	193	
Total	17	209	226	
Risk	.4705882	.1196172	.1460177	

	Point estimate	[95% Conf.	Interval]	
Risk difference	.350971	.1096576	.5922844	
Risk ratio	3.934118	2.107692	7.343238	
Attr. frac. ex.	.7458134	.5255473	.8638203	
Attr. frac. pop	.1808032			
Odds ratio	6.542222	2.381021	18.04016	(Cornfield)

chi2(1) = 15.53 Pr>chi2 = 0.0001

79 . cs EndPoint1 FRM30, or

	Respiratory rate > 30 bpm Exposed	Unexposed	Total
Cases	4	29	33
Noncases	6	187	193
Total	10	216	226
Risk	.4	.1342593	.1460177

	Point estimate	[95% Conf.	Interval]	
Risk difference	.2657407	-.0412807	.5727622	
Risk ratio	2.97931	1.297564	6.840733	
Attr. frac. ex.	.6643519	.2293253	.8538168	
Attr. frac. pop	.0805275			
Odds ratio	4.298851	1.227552	15.1628	(Cornfield)

chi2(1) = 5.41 Pr>chi2 = 0.0200

80 . tabulate EndPoint1 RxExt , chi2 column exact expected lrchi2

Key
frequency<br>*expected frequency*<br>*column percentage*

Enumerating sample-space combinations:
stage 3: enumerations = 1
stage 2: enumerations = 7
stage 1: enumerations = 0

Adverse event	Rx extension Unilobar	Multiloba	Bilateral	Total
No	151<br>147.7<br>87.28	23<br>23.1<br>85.19	19<br>22.2<br>73.08	193<br>193.0<br>85.40
Yes	22<br>25.3<br>12.72	4<br>3.9<br>14.81	7<br>3.8<br>26.92	33<br>33.0<br>14.60
Total	173	27	26	226

```
           |     173.0       27.0       26.0 |     226.0 
           |    100.00     100.00     100.00 |    100.00 

          Pearson chi2(2) =   3.6594   Pr = 0.160
 likelihood-ratio chi2(2) =   3.1577   Pr = 0.206
           Fisher's exact =                 0.152
```

81 . cs EndPoint1 Derrame, or

```
                 | Rx effusion            |
                 |   Exposed   Unexposed  |      Total
-----------------+------------------------+------------
           Cases |         6          27  |         33
        Noncases |        14         179  |        193
-----------------+------------------------+------------
           Total |        20         206  |        226
                 |                        |
            Risk |        .3     .131068  |   .1460177
                 |                        |
                 |      Point estimate    |    [95% Conf. Interval]
                 |------------------------+------------------------
 Risk difference |         .168932        |   -.037124    .3749881 
      Risk ratio |        2.288889        |    1.07454    4.875585 
 Attr. frac. ex. |        .5631068        |   .0693694    .7948964 
 Attr. frac. pop |        .1023831        |
      Odds ratio |         2.84127        |   1.040126    7.817551 (Cornfield)
                 +-------------------------------------------------
                               chi2(1) =     4.17  Pr>chi2 = 0.0411
```

82 . by EndPoint1, sort : tabstat Urea, statistics(median iqr)

```
-> EndPoint1 = No

    variable |       p50       iqr
-------------+--------------------
        Urea |        36        26
----------------------------------

-> EndPoint1 = Yes

    variable |       p50       iqr
-------------+--------------------
        Urea |        47        43
----------------------------------
```

83 . by EndPoint1, sort : tabstat Na, statistics(median iqr)

```
-> EndPoint1 = No

    variable |       p50       iqr
-------------+--------------------
          Na |       137         5
----------------------------------

-> EndPoint1 = Yes

    variable |       p50       iqr
-------------+--------------------
          Na |     136.5       9.5
----------------------------------
```

84 . by EndPoint1, sort : tabstat pO2, statistics(median iqr)

-> EndPoint1 = No

variable	p50	iqr
pO2	62	13

-> EndPoint1 = Yes

variable	p50	iqr
pO2	61	16

85 . by EndPoint1, sort : tabstat pH, statistics(median iqr)

-> EndPoint1 = No

variable	p50	iqr
pH	7.44	.07

-> EndPoint1 = Yes

variable	p50	iqr
pH	7.4	.15

86 . by EndPoint1, sort : tabstat Lactico, statistics(median iqr)

-> EndPoint1 = No

variable	p50	iqr
Lactico	1.4	.9

-> EndPoint1 = Yes

variable	p50	iqr
Lactico	2	1.6

87 . by EndPoint1, sort : tabstat Leucocitos, statistics(median iqr)

-> EndPoint1 = No

variable	p50	iqr
Leucocitos	11900	7700

-> EndPoint1 = Yes

variable	p50	iqr
Leucocitos	14400	8000

88 . by EndPoint1, sort : tabstat PCR, statistics(median iqr)

-> EndPoint1 = No

variable	p50	iqr
PCR	9.4	16.1

-> EndPoint1 = Yes

variable	p50	iqr
PCR	9.1	14

89 . by EndPoint1, sort : tabstat ProADM, statistics(median iqr)

-> EndPoint1 = No

variable	p50	iqr
ProADM	1.05	.77

-> EndPoint1 = Yes

variable	p50	iqr
ProADM	1.56	1.37

90 . by EndPoint1, sort : tabstat PCT, statistics(median iqr)

-> EndPoint1 = No

variable	p50	iqr
PCT	.12	.37

-> EndPoint1 = Yes

variable	p50	iqr
PCT	.22	1.43

91 . by EndPoint1, sort : tabstat ProBNP, statistics(median iqr)

-> EndPoint1 = No

variable	p50	iqr
ProBNP	384.5	1235.5

-> EndPoint1 = Yes

variable	p50	iqr
ProBNP	1621	3231

```
92 . by EndPoint1, sort : tabstat Age, statistics( median iqr )
```

-> EndPoint1 = No

variable	p50	iqr
Age	74.63655	29.07597

-> EndPoint1 = Yes

variable	p50	iqr
Age	80.92539	13.93292

```
93 . by EndPoint1, sort : tabstat PSI, statistics( median iqr )
```

-> EndPoint1 = No

variable	p50	iqr
PSI	81	46

-> EndPoint1 = Yes

variable	p50	iqr
PSI	122	59

```
94 . tabulate EndPoint1 PSI3 , chi2 column exact expected lrchi2
```

Key
frequency<br>*expected frequency*<br>*column percentage*

Enumerating sample-space combinations:
stage 3: enumerations = 1
stage 2: enumerations = 15
stage 1: enumerations = 0

Adverse event	PSI 3 categorias Mild	Moderate	Severe	Total
No	127<br>117.0<br>92.70	49<br>49.5<br>84.48	17<br>26.5<br>54.84	193<br>193.0<br>85.40
Yes	10<br>20.0<br>7.30	9<br>8.5<br>15.52	14<br>4.5<br>45.16	33<br>33.0<br>14.60
Total	137<br>137.0<br>100.00	58<br>58.0<br>100.00	31<br>31.0<br>100.00	226<br>226.0<br>100.00

```
          Pearson chi2(2) =  29.1145   Pr = 0.000
 likelihood-ratio chi2(2) =  23.5671   Pr = 0.000
           Fisher's exact =                 0.000
```

95 . tabulate EndPoint1 HC , chi2 column exact expected lrchi2

Key
frequency<br>*expected frequency*<br>*column percentage*

Enumerating sample-space combinations:
stage 3: enumerations = 1
stage 2: enumerations = 3
stage 1: enumerations = 0

Adverse event	Hemocultivos Negativo	Positivo	Contamina	Total
No	136<br>134.6<br>86.62	4<br>5.1<br>66.67	10<br>10.3<br>83.33	150<br>150.0<br>85.71
Yes	21<br>22.4<br>13.38	2<br>0.9<br>33.33	2<br>1.7<br>16.67	25<br>25.0<br>14.29
Total	157<br>157.0<br>100.00	6<br>6.0<br>100.00	12<br>12.0<br>100.00	175<br>175.0<br>100.00

```
         Pearson chi2(2) =   1.9395   Pr = 0.379
 likelihood-ratio chi2(2) =   1.5400   Pr = 0.463
           Fisher's exact =                 0.210
```

96 . cs EndPoint1 Destino, or

	Treatment site Exposed	Unexposed	Total
Cases	28	5	33
Noncases	155	38	193
Total	183	43	226
Risk	.1530055	.1162791	.1460177

	Point estimate	[95% Conf.	Interval]	
Risk difference	.0367264	-.0723627	.1458155	
Risk ratio	1.315847	.5394332	3.209764	
Attr. frac. ex.	.2400332	-.8537975	.6884506	
Attr. frac. pop	.2036646			
Odds ratio	1.372903	.5121665	3.656972	(Cornfield)

chi2(1) =   0.38 Pr>chi2 = 0.5394

97 .
98 . roctab EndPoint1 ProADM, detail graph

Detailed report of sensitivity and specificity

Cutpoint	Sensitivity	Specificity	Correctly Classified	LR+	LR-
(>= .33)	100.00%	0.00%	14.60%	1.0000	
(>= .34)	100.00%	0.52%	15.04%	1.0052	0.0000
(>= .35)	100.00%	1.04%	15.49%	1.0105	0.0000
(>= .36)	100.00%	1.55%	15.93%	1.0158	0.0000
(>= .37)	100.00%	2.07%	16.37%	1.0212	0.0000
(>= .4)	100.00%	2.59%	16.81%	1.0266	0.0000
(>= .41)	100.00%	3.63%	17.70%	1.0376	0.0000
(>= .42)	100.00%	4.15%	18.14%	1.0432	0.0000

(>= .43)	96.97%	5.18%	18.58%	1.0227	0.5848
(>= .44)	96.97%	6.22%	19.47%	1.0340	0.4874
(>= .45)	96.97%	7.25%	20.35%	1.0455	0.4177
(>= .48)	96.97%	8.29%	21.24%	1.0574	0.3655
(>= .49)	96.97%	8.81%	21.68%	1.0634	0.3440
(>= .51)	96.97%	9.33%	22.12%	1.0694	0.3249
(>= .52)	96.97%	10.36%	23.01%	1.0818	0.2924
(>= .53)	96.97%	11.40%	23.89%	1.0945	0.2658
(>= .55)	96.97%	12.44%	24.78%	1.1074	0.2437
(>= .56)	96.97%	13.47%	25.66%	1.1207	0.2249
(>= .57)	96.97%	14.51%	26.55%	1.1343	0.2089
(>= .58)	96.97%	15.54%	27.43%	1.1482	0.1949
(>= .61)	96.97%	16.58%	28.32%	1.1624	0.1828
(>= .62)	96.97%	18.13%	29.65%	1.1845	0.1671
(>= .63)	96.97%	19.69%	30.97%	1.2074	0.1539
(>= .64)	96.97%	20.73%	31.86%	1.2232	0.1462
(>= .66)	96.97%	21.24%	32.30%	1.2313	0.1426
(>= .67)	96.97%	22.28%	33.19%	1.2477	0.1360
(>= .68)	96.97%	22.80%	33.63%	1.2561	0.1329
(>= .7)	96.97%	24.35%	34.96%	1.2819	0.1244
(>= .71)	96.97%	25.91%	36.28%	1.3088	0.1170
(>= .72)	96.97%	26.94%	37.17%	1.3273	0.1125
(>= .73)	96.97%	27.46%	37.61%	1.3368	0.1103
(>= .74)	96.97%	27.98%	38.05%	1.3464	0.1083
(>= .75)	96.97%	28.50%	38.50%	1.3562	0.1063
(>= .77)	96.97%	29.02%	38.94%	1.3661	0.1044
(>= .78)	96.97%	29.53%	39.38%	1.3761	0.1026
(>= .79)	96.97%	30.05%	39.82%	1.3863	0.1008
(>= .8)	96.97%	31.09%	40.71%	1.4072	0.0975
(>= .82)	96.97%	33.68%	42.92%	1.4621	0.0900
(>= .83)	96.97%	34.72%	43.81%	1.4853	0.0873
(>= .84)	96.97%	35.23%	44.25%	1.4972	0.0860
(>= .85)	96.97%	35.75%	44.69%	1.5093	0.0848
(>= .86)	90.91%	38.34%	46.02%	1.4744	0.2371
(>= .87)	90.91%	38.86%	46.46%	1.4869	0.2339
(>= .88)	87.88%	39.38%	46.46%	1.4496	0.3078
(>= .89)	87.88%	39.90%	46.90%	1.4621	0.3038
(>= .9)	84.85%	39.90%	46.46%	1.4117	0.3798
(>= .91)	84.85%	41.45%	47.79%	1.4492	0.3655
(>= .92)	84.85%	42.49%	48.67%	1.4753	0.3566
(>= .93)	84.85%	43.01%	49.12%	1.4887	0.3523
(>= .96)	84.85%	43.52%	49.56%	1.5024	0.3481
(>= .97)	84.85%	44.04%	50.00%	1.5163	0.3440
(>= .98)	81.82%	44.56%	50.00%	1.4758	0.4080
(>= .99)	81.82%	45.60%	50.88%	1.5039	0.3988
(>= 1.02)	78.79%	46.11%	50.88%	1.4621	0.4600
(>= 1.03)	72.73%	46.63%	50.44%	1.3628	0.5848
(>= 1.04)	72.73%	48.70%	52.21%	1.4178	0.5600
(>= 1.05)	72.73%	49.74%	53.10%	1.4470	0.5483
(>= 1.06)	72.73%	50.78%	53.98%	1.4775	0.5371
(>= 1.07)	72.73%	52.33%	55.31%	1.5257	0.5212
(>= 1.08)	72.73%	53.89%	56.64%	1.5771	0.5061
(>= 1.09)	72.73%	54.40%	57.08%	1.5950	0.5013
(>= 1.1)	69.70%	54.92%	57.08%	1.5462	0.5517
(>= 1.11)	69.70%	55.96%	57.96%	1.5825	0.5415
(>= 1.12)	66.67%	56.48%	57.96%	1.5317	0.5902
(>= 1.14)	66.67%	56.99%	58.41%	1.5502	0.5848
(>= 1.15)	66.67%	58.03%	59.29%	1.5885	0.5744
(>= 1.17)	66.67%	58.55%	59.73%	1.6083	0.5693
(>= 1.18)	66.67%	59.07%	60.18%	1.6287	0.5643
(>= 1.19)	66.67%	60.10%	61.06%	1.6710	0.5546
(>= 1.2)	66.67%	61.14%	61.95%	1.7156	0.5452
(>= 1.21)	66.67%	62.18%	62.83%	1.7626	0.5361
(>= 1.22)	66.67%	62.69%	63.27%	1.7870	0.5317
(>= 1.23)	66.67%	63.21%	63.72%	1.8122	0.5273
(>= 1.26)	66.67%	63.73%	64.16%	1.8381	0.5230
(>= 1.28)	66.67%	64.25%	64.60%	1.8647	0.5188
(>= 1.29)	63.64%	64.77%	64.60%	1.8061	0.5615
(>= 1.3)	63.64%	65.28%	65.04%	1.8331	0.5570
(>= 1.31)	60.61%	67.36%	66.37%	1.8567	0.5848
(>= 1.32)	60.61%	67.88%	66.81%	1.8866	0.5804
(>= 1.33)	60.61%	68.91%	67.70%	1.9495	0.5717

(>= 1.34)	60.61%	69.43%	68.14%	1.9825	0.5674
(>= 1.35)	60.61%	70.47%	69.03%	2.0521	0.5590
(>= 1.36)	60.61%	70.98%	69.47%	2.0887	0.5550
(>= 1.38)	60.61%	71.50%	69.91%	2.1267	0.5509
(>= 1.39)	57.58%	72.02%	69.91%	2.0578	0.5891
(>= 1.42)	54.55%	72.02%	69.47%	1.9495	0.6311
(>= 1.43)	54.55%	72.54%	69.91%	1.9863	0.6266
(>= 1.44)	51.52%	73.06%	69.91%	1.9120	0.6637
(>= 1.45)	51.52%	74.09%	70.80%	1.9885	0.6544
(>= 1.47)	51.52%	74.61%	71.24%	2.0291	0.6498
(>= 1.5)	51.52%	75.65%	72.12%	2.1154	0.6409
(>= 1.51)	51.52%	76.17%	72.57%	2.1614	0.6366
(>= 1.53)	51.52%	77.72%	73.89%	2.3122	0.6238
(>= 1.54)	51.52%	78.24%	74.34%	2.3672	0.6197
(>= 1.55)	51.52%	78.76%	74.78%	2.4250	0.6156
(>= 1.56)	51.52%	79.79%	75.66%	2.5493	0.6076
(>= 1.57)	48.48%	80.31%	75.66%	2.4625	0.6414
(>= 1.59)	48.48%	80.83%	76.11%	2.5291	0.6373
(>= 1.6)	48.48%	82.38%	77.43%	2.7522	0.6253
(>= 1.61)	45.45%	82.90%	77.43%	2.6584	0.6580
(>= 1.65)	45.45%	83.42%	77.88%	2.7415	0.6539
(>= 1.67)	45.45%	83.94%	78.32%	2.8299	0.6498
(>= 1.69)	45.45%	84.97%	79.20%	3.0251	0.6419
(>= 1.7)	45.45%	85.49%	79.65%	3.1331	0.6380
(>= 1.71)	45.45%	86.01%	80.09%	3.2492	0.6342
(>= 1.74)	45.45%	86.53%	80.53%	3.3741	0.6304
(>= 1.8)	45.45%	87.05%	80.97%	3.5091	0.6266
(>= 1.82)	45.45%	87.56%	81.42%	3.6553	0.6229
(>= 1.89)	45.45%	88.60%	82.30%	3.9876	0.6156
(>= 1.97)	45.45%	89.12%	82.74%	4.1775	0.6121
(>= 2.03)	45.45%	89.64%	83.19%	4.3864	0.6085
(>= 2.06)	42.42%	89.64%	82.74%	4.0939	0.6423
(>= 2.12)	39.39%	89.64%	82.30%	3.8015	0.6761
(>= 2.19)	36.36%	89.64%	81.86%	3.5091	0.7099
(>= 2.22)	36.36%	90.16%	82.30%	3.6938	0.7059
(>= 2.25)	36.36%	90.67%	82.74%	3.8990	0.7018
(>= 2.27)	33.33%	90.67%	82.30%	3.5741	0.7352
(>= 2.29)	33.33%	91.19%	82.74%	3.7843	0.7311
(>= 2.31)	30.30%	91.19%	82.30%	3.4403	0.7643
(>= 2.35)	27.27%	91.19%	81.86%	3.0963	0.7975
(>= 2.37)	27.27%	91.71%	82.30%	3.2898	0.7930
(>= 2.39)	27.27%	92.23%	82.74%	3.5091	0.7886
(>= 2.42)	24.24%	92.23%	82.30%	3.1192	0.8214
(>= 2.44)	21.21%	92.75%	82.30%	2.9242	0.8495
(>= 2.5)	21.21%	93.26%	82.74%	3.1492	0.8448
(>= 2.55)	18.18%	93.26%	82.30%	2.6993	0.8773
(>= 2.66)	18.18%	94.30%	83.19%	3.1901	0.8676
(>= 2.72)	18.18%	94.82%	83.63%	3.5091	0.8629
(>= 2.99)	18.18%	95.34%	84.07%	3.8990	0.8582
(>= 3.23)	15.15%	95.85%	84.07%	3.6553	0.8852
(>= 3.29)	15.15%	96.37%	84.51%	4.1775	0.8804
(>= 3.3)	12.12%	96.37%	84.07%	3.3420	0.9119
(>= 3.86)	12.12%	96.89%	84.51%	3.8990	0.9070
(>= 3.9)	12.12%	97.41%	84.96%	4.6788	0.9022
(>= 4.24)	12.12%	97.93%	85.40%	5.8485	0.8974
(>= 4.36)	9.09%	97.93%	84.96%	4.3864	0.9283
(>= 4.5)	6.06%	97.93%	84.51%	2.9242	0.9593
(>= 4.52)	6.06%	98.45%	84.96%	3.8990	0.9542
(>= 4.68)	6.06%	98.96%	85.40%	5.8485	0.9492
(>= 4.75)	6.06%	99.48%	85.84%	11.6970	0.9443
(>= 5.14)	3.03%	99.48%	85.40%	5.8485	0.9747
(>= 13.6)	3.03%	100.00%	85.84%		0.9697
(> 13.6)	0.00%	100.00%	85.40%		1.0000

Obs	ROC Area	Std. Err.	—Asymptotic Normal— [95% Conf. Interval]	
226	0.7179	0.0474	0.62500	0.81071

```
 99 . roctab  EndPoint1 PSI

                        ROC                    —Asymptotic Normal——
            Obs        Area     Std. Err.      [95% Conf. Interval]
         ──────────────────────────────────────────────────────────
            226      0.7397       0.0536        0.63453     0.84482

100 . roctab  EndPoint1 ProBNP

                        ROC                    —Asymptotic Normal——
            Obs        Area     Std. Err.      [95% Conf. Interval]
         ──────────────────────────────────────────────────────────
            225      0.7344       0.0439        0.64834     0.82041

101 . roctab  EndPoint1 PCT

                        ROC                    —Asymptotic Normal——
            Obs        Area     Std. Err.      [95% Conf. Interval]
         ──────────────────────────────────────────────────────────
            224      0.6280       0.0556        0.51900     0.73691

102 . roctab  EndPoint1 PCR

                        ROC                    —Asymptotic Normal——
            Obs        Area     Std. Err.      [95% Conf. Interval]
         ──────────────────────────────────────────────────────────
            224      0.5157       0.0577        0.40252     0.62889

103 .
104 . roccomp EndPoint1 PSI ProADM

                                    ROC                    —Asymptotic Normal——
                        Obs        Area     Std. Err.      [95% Conf. Interval]
    ───────────────────────────────────────────────────────────────────────────
    PSI                 226      0.7397       0.0536        0.63453     0.84482
    ProADM              226      0.7179       0.0474        0.62500     0.81071
    ───────────────────────────────────────────────────────────────────────────
    Ho: area(PSI) = area(ProADM)
        chi2(1) =      0.30       Prob>chi2 =   0.5811

105 . roccomp EndPoint1 PSI ProBNP

                                    ROC                    —Asymptotic Normal——
                        Obs        Area     Std. Err.      [95% Conf. Interval]
    ───────────────────────────────────────────────────────────────────────────
    PSI                 225      0.7429       0.0537        0.63758     0.84822
    ProBNP              225      0.7344       0.0439        0.64834     0.82041
    ───────────────────────────────────────────────────────────────────────────
    Ho: area(PSI) = area(ProBNP)
        chi2(1) =      0.05       Prob>chi2 =   0.8223

106 . roccomp EndPoint1 PSI PCT

                                    ROC                    —Asymptotic Normal——
                        Obs        Area     Std. Err.      [95% Conf. Interval]
    ───────────────────────────────────────────────────────────────────────────
    PSI                 224      0.7382       0.0537        0.63292     0.84352
    PCT                 224      0.6280       0.0556        0.51900     0.73691
    ───────────────────────────────────────────────────────────────────────────
    Ho: area(PSI) = area(PCT)
        chi2(1) =      2.73       Prob>chi2 =   0.0987
```

```
107 .
108 . *Table 2
109 . logistic EndPoint1 PSI ProADM ProBNP

    Logistic regression                               Number of obs   =        225
                                                      LR chi2(3)      =      25.38
                                                      Prob > chi2     =     0.0000
    Log likelihood = -81.111209                       Pseudo R2       =     0.1353
```

EndPoint1	Odds Ratio	Std. Err.	z	P>|z|	[95% Conf.	Interval]
PSI	1.018638	.0068575	2.74	0.006	1.005285	1.032167
ProADM	1.158511	.2439571	0.70	0.485	.7667532	1.750429
ProBNP	1.000029	.0000524	0.56	0.573	.9999268	1.000132
_cons	.0212151	.012477	-6.55	0.000	.0066994	.0671821

```
110 . estat gof
```

Logistic model for EndPoint1, goodness-of-fit test

```
       number of observations =       225
 number of covariate patterns =       225
            Pearson chi2(221) =       240.94
                  Prob > chi2 =         0.1704

111 . allsets EndPoint1 PSI  ProADM ProBNP , logistic
```

ALLSETS - Logistic regression

ALL VARIABLES
Dependent: **EndPoint1**
Continuous: **PSI ProADM ProBNP**

Variable	Valid	Missing
EndPoint1	226	0
PSI	226	0
ProADM	226	0
ProBNP	225	1

Valid number of cases (listwise): **225**
Total number of hierarchical submodels estimated: **7**
Total time: **0.2** seconds

A new dataset has been created with the results
Execute **use "allsets_results.dta"** to open the dataset with the results

stats	AIC	BIC	AUC	Se	Sp	_2ll	pfitHL
min	167	174	.721	3.03	97.9	162	.000209
max	183	190	.748	15.2	99	179	.858

```
112 . save "C:\Users\susan_000\Documents\NACURG\NUEVO\NACURGPLOS",replace
    file C:\Users\susan_000\Documents\NACURG\NUEVO\NACURGPLOS.dta saved

113 . use "allsets_results.dta"
```

114 . list NVar - pfitHL, clean noobs

NVar	Variables	AIC	BIC	AUC	Se	Sp	_2ll	pfitHL
1	PSI	167.4	174.2	0.743	12.1	99.0	163.4	0.000
2	PSI ProADM	168.5	178.8	0.746	12.1	98.4	162.5	0.147
2	PSI ProBNP	168.8	179.1	0.744	15.2	97.9	162.8	0.082
3	PSI ProADM ProBNP	170.2	183.9	0.748	12.1	98.4	162.2	0.170
1	ProADM	174.3	181.2	0.721	12.1	98.4	170.3	0.858
2	ProADM ProBNP	175.6	185.8	0.734	12.1	98.4	169.6	0.675
1	ProBNP	183.1	189.9	0.734	3.0	99.0	179.1	0.582

115 . use "C:\Users\susan_000\Documents\NACURG\NUEVO\NACURGPLOS.dta"
(Estudio NACURG)

116 .
117 . logistic EndPoint1 PSI

```
Logistic regression                               Number of obs   =        226
                                                  LR chi2(1)      =      23.06
                                                  Prob > chi2     =     0.0000
Log likelihood = -82.425746                       Pseudo R2       =     0.1227
```

EndPoint1	Odds Ratio	Std. Err.	z	P>|z|	[95% Conf.	Interval]
PSI	1.022486	.0050356	4.52	0.000	1.012663	1.032403
_cons	.0193929	.0109752	-6.97	0.000	.0063961	.058799

118 . estat gof

Logistic model for EndPoint1, goodness-of-fit test

```
       number of observations =       226
 number of covariate patterns =       116
            Pearson chi2(114) =       170.62
                  Prob > chi2 =         0.0005
```

119 . fitstat

Measures of Fit for **logistic** of **EndPoint1**

Log-Lik Intercept Only:	-93.957	Log-Lik Full Model:	-82.426
D(224):	164.851	LR(1):	23.062
		Prob > LR:	0.000
McFadden's R2:	0.123	McFadden's Adj R2:	0.101
ML (Cox-Snell) R2:	0.097	Cragg-Uhler(Nagelkerke) R2:	0.172
McKelvey & Zavoina's R2:	0.193	Efron's R2:	0.136
Variance of y*:	4.077	Variance of error:	3.290
Count R2:	0.863	Adj Count R2:	0.061
AIC:	0.747	AIC*n:	168.851
BIC:	-1049.348	BIC':	-17.642
BIC used by Stata:	175.693	AIC used by Stata:	168.851

120 . logistic EndPoint1 PSI ProADM

```
Logistic regression                               Number of obs   =        226
                                                  LR chi2(2)      =      23.73
                                                  Prob > chi2     =     0.0000
Log likelihood = -82.093105                       Pseudo R2       =     0.1263
```

EndPoint1	Odds Ratio	Std. Err.	z	P>|z|	[95% Conf.	Interval]
PSI	1.018953	.0066858	2.86	0.004	1.005933	1.032141
ProADM	1.161395	.2310085	0.75	0.452	.7864497	1.715099
_cons	.0215805	.0123896	-6.68	0.000	.0070045	.0664883

```
121 . estat gof

    Logistic model for EndPoint1, goodness-of-fit test

           number of observations =       226
     number of covariate patterns =       224
                Pearson chi2(221) =       240.84
                      Prob > chi2 =         0.1715

122 . fitstat

    Measures of Fit for logistic of EndPoint1

    Log-Lik Intercept Only:        -93.957   Log-Lik Full Model:                -82.093
    D(223):                        164.186   LR(2):                              23.728
                                             Prob > LR:                           0.000
    McFadden's R2:                   0.126   McFadden's Adj R2:                   0.094
    ML (Cox-Snell) R2:               0.100   Cragg-Uhler(Nagelkerke) R2:          0.177
    McKelvey & Zavoina's R2:         0.190   Efron's R2:                          0.134
    Variance of y*:                  4.061   Variance of error:                   3.290
    Count R2:                        0.863   Adj Count R2:                        0.061
    AIC:                             0.753   AIC*n:                             170.186
    BIC:                         -1044.593   BIC':                              -12.887
    BIC used by Stata:             180.448   AIC used by Stata:                 170.186

123 . set seed 20131112

124 . crossfold logit EndPoint1 PSI ProADM, r2

                 |  Pseudo-R2
    -------------+-----------
            est1 |  .0000528
            est2 |   .246918
            est3 |   .424912
            est4 |  .0465819
            est5 |  .1195042

125 . mata: mean(st_matrix("r(est)"))
      .1675937755

126 . *PSI & ProADM model vs PSI alone
127 . predict EndPoint1_Pre, p

128 . roctab EndPoint1 EndPoint1_Pre, detail graph

    Detailed report of sensitivity and specificity
```

Cutpoint	Sensitivity	Specificity	Correctly Classified	LR+	LR-
(>= .0252..)	100.00%	0.00%	14.60%	1.0000	
(>= .027388)	100.00%	0.52%	15.04%	1.0052	0.0000
(>= .0287..)	96.97%	0.52%	14.60%	0.9747	5.8485
(>= .0311..)	96.97%	1.04%	15.04%	0.9799	2.9242
(>= .0315..)	96.97%	1.55%	15.49%	0.9850	1.9495
(>= .0315..)	96.97%	2.07%	15.93%	0.9902	1.4621
(>= .032201)	96.97%	2.59%	16.37%	0.9955	1.1697
(>= .0323..)	96.97%	3.11%	16.81%	1.0008	0.9747
(>= .0329..)	96.97%	3.63%	17.26%	1.0062	0.8355
(>= .0340..)	93.94%	3.63%	16.81%	0.9747	1.6710
(>= .0346..)	93.94%	4.15%	17.26%	0.9800	1.4621
(>= .034693)	93.94%	4.66%	17.70%	0.9853	1.2997
(>= .0352..)	93.94%	5.18%	18.14%	0.9907	1.1697
(>= .0359..)	93.94%	5.70%	18.58%	0.9962	1.0634
(>= .0366..)	93.94%	6.22%	19.03%	1.0017	0.9747
(>= .0373..)	93.94%	6.74%	19.47%	1.0072	0.8998
(>= .0375..)	93.94%	7.25%	19.91%	1.0129	0.8355
(>= .0377..)	93.94%	7.77%	20.35%	1.0186	0.7798
(>= .0380..)	93.94%	8.29%	20.80%	1.0243	0.7311
(>= .0386..)	93.94%	8.81%	21.24%	1.0301	0.6881
(>= .0393..)	93.94%	9.33%	21.68%	1.0360	0.6498
(>= .039906)	93.94%	9.84%	22.12%	1.0420	0.6156

(>= .0402..)	93.94%	10.36%	22.57%	1.0480	0.5848
(>= .0407..)	93.94%	10.88%	23.01%	1.0541	0.5570
(>= .0410..)	93.94%	11.40%	23.45%	1.0603	0.5317
(>= .043731)	93.94%	11.92%	23.89%	1.0665	0.5086
(>= .0441..)	93.94%	12.44%	24.34%	1.0728	0.4874
(>= .0442..)	93.94%	12.95%	24.78%	1.0792	0.4679
(>= .0444..)	93.94%	13.47%	25.22%	1.0856	0.4499
(>= .0444..)	93.94%	13.99%	25.66%	1.0922	0.4332
(>= .0445..)	93.94%	14.51%	26.11%	1.0988	0.4177
(>= .0448..)	93.94%	15.03%	26.55%	1.1055	0.4033
(>= .0458..)	93.94%	15.54%	26.99%	1.1123	0.3899
(>= .0463..)	93.94%	16.06%	27.43%	1.1192	0.3773
(>= .0466..)	90.91%	16.06%	26.99%	1.0831	0.5660
(>= .0487..)	90.91%	16.58%	27.43%	1.0898	0.5483
(>= .0492..)	90.91%	17.10%	27.88%	1.0966	0.5317
(>= .0493..)	90.91%	17.62%	28.32%	1.1035	0.5160
(>= .0499..)	90.91%	18.13%	28.76%	1.1105	0.5013
(>= .0500..)	90.91%	18.65%	29.20%	1.1175	0.4874
(>= .050714)	90.91%	19.17%	29.65%	1.1247	0.4742
(>= .0508..)	90.91%	19.69%	30.09%	1.1320	0.4617
(>= .0514..)	90.91%	20.21%	30.53%	1.1393	0.4499
(>= .0518..)	90.91%	20.73%	30.97%	1.1468	0.4386
(>= .0522..)	90.91%	21.24%	31.42%	1.1543	0.4279
(>= .0523..)	90.91%	21.76%	31.86%	1.1620	0.4177
(>= .0533..)	90.91%	22.28%	32.30%	1.1697	0.4080
(>= .0536..)	90.91%	22.80%	32.74%	1.1775	0.3988
(>= .0548..)	87.88%	22.80%	32.30%	1.1383	0.5317
(>= .0564..)	87.88%	23.32%	32.74%	1.1460	0.5199
(>= .0578..)	87.88%	23.83%	33.19%	1.1538	0.5086
(>= .0582..)	87.88%	24.35%	33.63%	1.1617	0.4977
(>= .0589..)	87.88%	24.87%	34.07%	1.1697	0.4874
(>= .0599..)	87.88%	25.39%	34.51%	1.1778	0.4774
(>= .0608..)	87.88%	25.91%	34.96%	1.1861	0.4679
(>= .0610..)	87.88%	26.42%	35.40%	1.1944	0.4587
(>= .0620..)	87.88%	26.94%	35.84%	1.2029	0.4499
(>= .0646..)	87.88%	27.46%	36.28%	1.2115	0.4414
(>= .065136)	87.88%	27.98%	36.73%	1.2202	0.4332
(>= .0659..)	87.88%	28.50%	37.17%	1.2290	0.4253
(>= .0682..)	87.88%	29.02%	37.61%	1.2380	0.4177
(>= .0684..)	87.88%	29.53%	38.05%	1.2471	0.4104
(>= .0685..)	87.88%	30.05%	38.50%	1.2563	0.4033
(>= .0686..)	87.88%	30.57%	38.94%	1.2657	0.3965
(>= .0688..)	87.88%	31.09%	39.38%	1.2752	0.3899
(>= .0694..)	87.88%	31.61%	39.82%	1.2849	0.3835
(>= .070368)	87.88%	32.12%	40.27%	1.2947	0.3773
(>= .0704..)	87.88%	32.64%	40.71%	1.3047	0.3713
(>= .070585)	87.88%	33.16%	41.15%	1.3148	0.3655
(>= .0706..)	87.88%	33.68%	41.59%	1.3250	0.3599
(>= .070818)	87.88%	34.20%	42.04%	1.3355	0.3545
(>= .0713..)	87.88%	34.72%	42.48%	1.3461	0.3492
(>= .0717..)	87.88%	35.23%	42.92%	1.3568	0.3440
(>= .0722..)	87.88%	35.75%	43.36%	1.3678	0.3390
(>= .0726..)	87.88%	36.27%	43.81%	1.3789	0.3342
(>= .0740..)	84.85%	36.27%	43.36%	1.3314	0.4177
(>= .0744..)	84.85%	36.79%	43.81%	1.3423	0.4119
(>= .0745..)	84.85%	37.31%	44.25%	1.3534	0.4061
(>= .0756..)	84.85%	37.82%	44.69%	1.3646	0.4006
(>= .0766..)	84.85%	38.34%	45.13%	1.3761	0.3952
(>= .0783..)	84.85%	38.86%	45.58%	1.3878	0.3899
(>= .0787..)	84.85%	39.90%	46.46%	1.4117	0.3798
(>= .0803..)	84.85%	40.41%	46.90%	1.4240	0.3749
(>= .0810..)	84.85%	40.93%	47.35%	1.4365	0.3702
(>= .0822..)	84.85%	41.45%	47.79%	1.4492	0.3655
(>= .0824..)	81.82%	41.45%	47.35%	1.3974	0.4386
(>= .0827..)	81.82%	41.97%	47.79%	1.4099	0.4332
(>= .0847..)	81.82%	42.49%	48.23%	1.4226	0.4279
(>= .0859..)	81.82%	43.01%	48.67%	1.4355	0.4228
(>= .0860..)	81.82%	43.52%	49.12%	1.4487	0.4177
(>= .08679)	81.82%	44.04%	49.56%	1.4621	0.4128
(>= .0872..)	81.82%	44.56%	50.00%	1.4758	0.4080
(>= .0880..)	81.82%	45.08%	50.44%	1.4897	0.4033
(>= .0882..)	81.82%	45.60%	50.88%	1.5039	0.3988

(>= .0906..)	81.82%	46.11%	51.33%	1.5184	0.3943
(>= .0910..)	81.82%	46.63%	51.77%	1.5331	0.3899
(>= .0927..)	81.82%	47.15%	52.21%	1.5481	0.3856
(>= .0929..)	81.82%	47.67%	52.65%	1.5635	0.3814
(>= .0932..)	81.82%	48.19%	53.10%	1.5791	0.3773
(>= .0943..)	81.82%	48.70%	53.54%	1.5950	0.3733
(>= .0945..)	81.82%	49.22%	53.98%	1.6113	0.3694
(>= .0953..)	81.82%	49.74%	54.42%	1.6279	0.3655
(>= .0964..)	81.82%	50.26%	54.87%	1.6449	0.3618
(>= .0983..)	81.82%	50.78%	55.31%	1.6622	0.3581
(>= .0986..)	81.82%	51.30%	55.75%	1.6799	0.3545
(>= .0994..)	81.82%	51.81%	56.19%	1.6979	0.3509
(>= .1007..)	81.82%	52.33%	56.64%	1.7164	0.3474
(>= .101439)	81.82%	52.85%	57.08%	1.7353	0.3440
(>= .1025..)	81.82%	53.37%	57.52%	1.7545	0.3407
(>= .102937)	81.82%	53.89%	57.96%	1.7743	0.3374
(>= .1032..)	81.82%	54.40%	58.41%	1.7944	0.3342
(>= .1048..)	81.82%	54.92%	58.85%	1.8150	0.3310
(>= .1054..)	81.82%	55.44%	59.29%	1.8362	0.3280
(>= .1071..)	81.82%	55.96%	59.73%	1.8578	0.3249
(>= .10726)	81.82%	56.48%	60.18%	1.8799	0.3219
(>= .1074..)	81.82%	56.99%	60.62%	1.9025	0.3190
(>= .1076..)	81.82%	57.51%	61.06%	1.9257	0.3161
(>= .1076..)	81.82%	58.03%	61.50%	1.9495	0.3133
(>= .1078..)	81.82%	58.55%	61.95%	1.9739	0.3105
(>= .1082..)	78.79%	58.55%	61.50%	1.9008	0.3623
(>= .1083..)	78.79%	59.07%	61.95%	1.9248	0.3591
(>= .109178)	78.79%	59.59%	62.39%	1.9495	0.3560
(>= .1092..)	78.79%	60.10%	62.83%	1.9748	0.3529
(>= .1100..)	78.79%	60.62%	63.27%	2.0008	0.3499
(>= .1111..)	78.79%	61.14%	63.72%	2.0275	0.3469
(>= .1116..)	78.79%	61.66%	64.16%	2.0549	0.3440
(>= .1124..)	78.79%	62.18%	64.60%	2.0830	0.3412
(>= .114535)	78.79%	62.69%	65.04%	2.1120	0.3383
(>= .1152..)	78.79%	63.21%	65.49%	2.1417	0.3356
(>= .1163..)	78.79%	63.73%	65.93%	2.1723	0.3328
(>= .1166..)	75.76%	63.73%	65.49%	2.0887	0.3804
(>= .1169..)	75.76%	64.25%	65.93%	2.1190	0.3773
(>= .1174..)	75.76%	64.77%	66.37%	2.1502	0.3743
(>= .1179..)	75.76%	65.28%	66.81%	2.1823	0.3713
(>= .1180..)	75.76%	65.80%	67.26%	2.2153	0.3684
(>= .1183..)	75.76%	66.32%	67.70%	2.2494	0.3655
(>= .1206..)	72.73%	66.32%	67.26%	2.1594	0.4112
(>= .1206..)	72.73%	66.84%	67.70%	2.1932	0.4080
(>= .1231..)	69.70%	66.84%	67.26%	2.1018	0.4534
(>= .1251..)	69.70%	67.36%	67.70%	2.1352	0.4499
(>= .1254..)	69.70%	67.88%	68.14%	2.1696	0.4464
(>= .1266..)	69.70%	68.39%	68.58%	2.2052	0.4431
(>= .1266..)	69.70%	68.91%	69.03%	2.2419	0.4397
(>= .1272..)	69.70%	69.43%	69.47%	2.2799	0.4365
(>= .1337..)	66.67%	69.43%	69.03%	2.1808	0.4801
(>= .1344..)	66.67%	69.95%	69.47%	2.2184	0.4765
(>= .136088)	66.67%	70.47%	69.91%	2.2573	0.4730
(>= .1363..)	66.67%	70.98%	70.35%	2.2976	0.4696
(>= .1367..)	66.67%	71.50%	70.80%	2.3394	0.4662
(>= .1369..)	63.64%	71.50%	70.35%	2.2331	0.5086
(>= .1372..)	63.64%	72.02%	70.80%	2.2744	0.5049
(>= .1383..)	63.64%	72.54%	71.24%	2.3173	0.5013
(>= .1388..)	63.64%	73.06%	71.68%	2.3619	0.4977
(>= .1395..)	60.61%	73.06%	71.24%	2.2494	0.5392
(>= .140352)	60.61%	73.58%	71.68%	2.2935	0.5354
(>= .1418..)	60.61%	74.61%	72.57%	2.3871	0.5280
(>= .1446..)	60.61%	75.13%	73.01%	2.4369	0.5243
(>= .1453..)	60.61%	75.65%	73.45%	2.4887	0.5208
(>= .1473..)	60.61%	76.17%	73.89%	2.5428	0.5172
(>= .1481..)	57.58%	76.17%	73.45%	2.4157	0.5570
(>= .1486..)	57.58%	76.68%	73.89%	2.4694	0.5532
(>= .1487..)	57.58%	77.20%	74.34%	2.5255	0.5495
(>= .1492..)	57.58%	77.72%	74.78%	2.5842	0.5459
(>= .1493..)	57.58%	78.24%	75.22%	2.6457	0.5422
(>= .1533..)	57.58%	78.76%	75.66%	2.7103	0.5387
(>= .1554..)	57.58%	79.27%	76.11%	2.7780	0.5352

(>= .1556..)	57.58%	79.79%	76.55%	2.8493	0.5317
(>= .156192)	57.58%	80.31%	76.99%	2.9242	0.5283
(>= .1577..)	57.58%	80.83%	77.43%	3.0033	0.5249
(>= .1600..)	54.55%	80.83%	76.99%	2.8452	0.5624
(>= .16063)	51.52%	80.83%	76.55%	2.6871	0.5998
(>= .1639..)	51.52%	81.35%	76.99%	2.7618	0.5960
(>= .1782..)	51.52%	81.87%	77.43%	2.8407	0.5923
(>= .1791..)	51.52%	82.38%	77.88%	2.9242	0.5885
(>= .180174)	51.52%	82.90%	78.32%	3.0129	0.5848
(>= .1808..)	51.52%	83.42%	78.76%	3.1070	0.5812
(>= .1821..)	51.52%	83.94%	79.20%	3.2072	0.5776
(>= .1870..)	51.52%	84.46%	79.65%	3.3141	0.5741
(>= .1874..)	51.52%	84.97%	80.09%	3.4284	0.5706
(>= .187436)	51.52%	85.49%	80.53%	3.5509	0.5671
(>= .1881..)	51.52%	86.01%	80.97%	3.6824	0.5637
(>= .1885..)	51.52%	86.53%	81.42%	3.8240	0.5603
(>= .1923..)	51.52%	87.05%	81.86%	3.9770	0.5570
(>= .1948..)	51.52%	87.56%	82.30%	4.1427	0.5537
(>= .1965..)	51.52%	88.08%	82.74%	4.3228	0.5504
(>= .1993..)	51.52%	88.60%	83.19%	4.5193	0.5472
(>= .2022..)	51.52%	89.12%	83.63%	4.7345	0.5440
(>= .2037..)	51.52%	89.64%	84.07%	4.9712	0.5409
(>= .2118..)	48.48%	89.64%	83.63%	4.6788	0.5747
(>= .2147..)	45.45%	89.64%	83.19%	4.3864	0.6085
(>= .2298..)	45.45%	90.16%	83.63%	4.6172	0.6050
(>= .2444..)	45.45%	90.67%	84.07%	4.8737	0.6016
(>= .250776)	42.42%	90.67%	83.63%	4.5488	0.6350
(>= .262312)	42.42%	91.19%	84.07%	4.8164	0.6314
(>= .2645..)	42.42%	91.71%	84.51%	5.1174	0.6278
(>= .2677..)	39.39%	91.71%	84.07%	4.7519	0.6608
(>= .2694..)	39.39%	92.23%	84.51%	5.0687	0.6571
(>= .2747..)	39.39%	92.75%	84.96%	5.4307	0.6535
(>= .2749..)	36.36%	92.75%	84.51%	5.0130	0.6861
(>= .2751..)	36.36%	93.26%	84.96%	5.3986	0.6823
(>= .2797..)	36.36%	93.78%	85.40%	5.8485	0.6786
(>= .2901..)	36.36%	94.30%	85.84%	6.3802	0.6748
(>= .2971..)	36.36%	94.82%	86.28%	7.0182	0.6711
(>= .3019..)	36.36%	95.34%	86.73%	7.7980	0.6675
(>= .3054..)	36.36%	95.85%	87.17%	8.7727	0.6639
(>= .3089..)	33.33%	95.85%	86.73%	8.0417	0.6955
(>= .3118..)	33.33%	96.37%	87.17%	9.1905	0.6918
(>= .3278..)	30.30%	96.37%	86.73%	8.3550	0.7232
(>= .3377..)	27.27%	96.37%	86.28%	7.5195	0.7546
(>= .3590..)	24.24%	96.37%	85.84%	6.6840	0.7861
(>= .3695..)	21.21%	96.37%	85.40%	5.8485	0.8175
(>= .3721..)	18.18%	96.37%	84.96%	5.0130	0.8490
(>= .3904..)	18.18%	96.89%	85.40%	5.8485	0.8444
(>= .4346..)	15.15%	96.89%	84.96%	4.8737	0.8757
(>= .4384..)	12.12%	96.89%	84.51%	3.8990	0.9070
(>= .447915)	12.12%	97.41%	84.96%	4.6788	0.9022
(>= .4762..)	12.12%	97.93%	85.40%	5.8485	0.8974
(>= .4763..)	12.12%	98.45%	85.84%	7.7980	0.8927
(>= .5100..)	12.12%	98.96%	86.28%	11.6970	0.8880
(>= .5270..)	9.09%	98.96%	85.84%	8.7727	0.9186
(>= .6081..)	9.09%	99.48%	86.28%	17.5454	0.9138
(>= .6894..)	6.06%	99.48%	85.84%	11.6970	0.9443
(>= .7617..)	3.03%	99.48%	85.40%	5.8485	0.9747
(>= .8041..)	3.03%	100.00%	85.84%		0.9697
(> .8041..)	0.00%	100.00%	85.40%		1.0000

Obs	ROC Area	Std. Err.	—Asymptotic Normal— [95% Conf.	Interval]
226	0.7430	0.0531	0.63887	0.84707

129 . *NRI
130 . nri3 EndPoint1 PSI, prvars(PSI ProADM) cut(5 10 20)

NRI	Estimate	Std. Err.	Z	P-value
	0.07694	0.03635	2.11679	0.03428

EndPoint1 and Established risk factors	Established risk factors + new predictors <5%	5-10%	10 - 20%	>=20%	Total
1					
<5%	2	1			3
5-10%		3			3
10 - 20%			10		10
>=20%				17	17
Total	2	4	10	17	33
0					
<5%	29	3			32
5-10%		59			59
10 - 20%		5	67		72
>=20%			7	23	30
Total	29	67	74	23	193

131 .
132 .
133 . **SECUNDARY OUTCOME: MR-PROADM LEVELS AND MID-TERM MORTALITY
134 . tab1 MortGlo

-> tabulation of MortGlo

90-Day Mortality	Freq.	Percent	Cum.
No	216	95.58	95.58
Yes	10	4.42	100.00
Total	226	100.00	

135 . stset TSup, f(MortGlo==1)

```
     failure event:  MortGlo == 1
obs. time interval:  (0, TSup]
 exit on or before:  failure

------------------------------------------------------------------------------
      226  total obs.
        0  exclusions
------------------------------------------------------------------------------
      226  obs. remaining, representing
       10  failures in single record/single failure data
    19812  total analysis time at risk, at risk from t =         0
                             earliest observed entry t =         0
                                  last observed exit t =        90
```

```
136 .
137 . stcox Age

         failure _d:  MortGlo == 1
   analysis time _t:  TSup

Iteration 0:   log likelihood = -54.003385
Iteration 1:   log likelihood = -48.941016
Iteration 2:   log likelihood = -47.533017
Iteration 3:   log likelihood = -47.332306
Iteration 4:   log likelihood = -47.329041
Iteration 5:   log likelihood = -47.329041
Refining estimates:
Iteration 0:   log likelihood = -47.329041

Cox regression -- no ties

No. of subjects =          226                   Number of obs   =        226
No. of failures =           10
Time at risk    =        19812
                                                 LR chi2(1)      =      13.35
Log likelihood  =   -47.329041                   Prob > chi2     =     0.0003
```

_t	Haz. Ratio	Std. Err.	z	P>|z|	[95% Conf.	Interval]
Age	1.117454	.045877	2.70	0.007	1.031059	1.211088

```
138 . estat phtest

      Test of proportional-hazards assumption

      Time:  Time
```

	chi2	df	Prob>chi2
global test	0.03	1	0.8706

```
139 . by MortGlo, sort : summarize Age, detail

-> MortGlo = No

                         Age (years)
-------------------------------------------------------------
      Percentiles      Smallest
 1%     20.53114       19.08282
 5%     29.38261       20.07392
10%     37.67556       20.53114       Obs                 216
25%     55.36893       20.59138       Sum of Wgt.         216

50%     74.22587                      Mean           67.90249
                        Largest       Std. Dev.      19.54299
75%     83.38535       93.87269
90%     88.39151       96.68994       Variance       381.9284
95%     89.79329       97.51129       Skewness      -.7747369
99%     96.68994       100.5394       Kurtosis       2.601299

-> MortGlo = Yes

                         Age (years)
-------------------------------------------------------------
      Percentiles      Smallest
 1%      77.7139        77.7139
 5%      77.7139       80.77755
10%     79.24572       82.37372       Obs                  10
25%     82.37372       83.87953       Sum of Wgt.          10
```

```
50%      87.05818                        Mean           86.00329
                         Largest         Std. Dev.      4.955814
75%      87.83573        87.75907
90%       92.7885        87.83573        Variance       24.56009
95%       94.6037         90.9733        Skewness       .0030114
99%       94.6037         94.6037        Kurtosis       2.408105
```

```
140 . sts, by(Sex) risktable

         failure _d:  MortGlo == 1
   analysis time _t:  TSup

141 . sts list, by(Sex)

         failure _d:  MortGlo == 1
   analysis time _t:  TSup
```

Time	Beg. Total	Fail	Net Lost	Survivor Function	Std. Error	[95% Conf.	Int.]
Female							
88	101	1	0	0.9901	0.0099	0.9318	0.9986
90	100	0	100	0.9901	0.0099	0.9318	0.9986
Male							
2	125	1	0	0.9920	0.0080	0.9446	0.9989
3	124	1	0	0.9840	0.0112	0.9375	0.9960
5	123	1	0	0.9760	0.0137	0.9274	0.9922
29	122	1	0	0.9680	0.0157	0.9170	0.9879
31	121	1	0	0.9600	0.0175	0.9066	0.9832
37	120	1	0	0.9520	0.0191	0.8963	0.9781
45	119	1	0	0.9440	0.0206	0.8861	0.9729
56	118	1	0	0.9360	0.0219	0.8761	0.9675
76	117	1	0	0.9280	0.0231	0.8662	0.9619
90	116	0	116	0.9280	0.0231	0.8662	0.9619

```
142 . sts test  Sex

         failure _d:  MortGlo == 1
   analysis time _t:  TSup
```

Log-rank test for equality of survivor functions

Sex	Events observed	Events expected
Female	1	4.56
Male	9	5.44
Total	10	10.00

```
          chi2(1) =       5.11
          Pr>chi2 =     0.0238

143 . by MortGlo, sort: tabulate Sex

-> MortGlo = No
```

Sex	Freq.	Percent	Cum.
Female	100	46.30	46.30
Male	116	53.70	100.00
Total	216	100.00	

```
-> MortGlo = Yes
```

Sex	Freq.	Percent	Cum.
Female	1	10.00	10.00
Male	9	90.00	100.00
Total	10	100.00	

```
144 . sts, by(CCharlson) risktable

         failure _d:  MortGlo == 1
   analysis time _t:  TSup

145 . sts list, by(CCharlson)

         failure _d:  MortGlo == 1
   analysis time _t:  TSup
```

Time	Beg. Total	Fail	Net Lost	Survivor Function	Std. Error	[95% Conf.	Int.]
No							
3	177	1	0	0.9944	0.0056	0.9606	0.9992
29	176	1	0	0.9887	0.0079	0.9556	0.9972
37	175	1	0	0.9831	0.0097	0.9484	0.9945
45	174	1	0	0.9774	0.0112	0.9409	0.9915
90	173	0	173	0.9774	0.0112	0.9409	0.9915
Yes							
2	49	1	0	0.9796	0.0202	0.8638	0.9971
5	48	1	0	0.9592	0.0283	0.8465	0.9896
31	47	1	0	0.9388	0.0342	0.8221	0.9798
56	46	1	0	0.9184	0.0391	0.7970	0.9686
76	45	1	0	0.8980	0.0432	0.7721	0.9562
88	44	1	0	0.8776	0.0468	0.7476	0.9430
90	43	0	43	0.8776	0.0468	0.7476	0.9430

```
146 . sts test CCharlson

         failure _d:  MortGlo == 1
   analysis time _t:  TSup
```

Log-rank test for equality of survivor functions

CCharlson	Events observed	Events expected
No	4	7.90
Yes	6	2.10
Total	10	10.00

```
             chi2(1) =       9.14
             Pr>chi2 =     0.0025

147 . by MortGlo, sort: tabulate CCharlson
```

-> MortGlo = No

Charlson>=3	Freq.	Percent	Cum.
No	173	80.09	80.09
Yes	43	19.91	100.00
Total	216	100.00	

-> MortGlo = Yes

Charlson>=3	Freq.	Percent	Cum.
No	4	40.00	40.00
Yes	6	60.00	100.00
Total	10	100.00	

```
148 . sts, by(Confu) risktable

         failure _d:  MortGlo == 1
   analysis time _t:  TSup

149 . sts list, by(Confu)

         failure _d:  MortGlo == 1
   analysis time _t:  TSup
```

Time	Beg. Total	Fail	Net Lost	Survivor Function	Std. Error	[95% Conf.	Int.]
No							
3	209	1	0	0.9952	0.0048	0.9665	0.9993
5	208	1	0	0.9904	0.0067	0.9623	0.9976
31	207	1	0	0.9856	0.0082	0.9562	0.9953
37	206	1	0	0.9809	0.0095	0.9498	0.9928
45	205	1	0	0.9761	0.0106	0.9435	0.9900
56	204	1	0	0.9713	0.0116	0.9372	0.9870
88	203	1	0	0.9665	0.0124	0.9310	0.9839
90	202	0	202	0.9665	0.0124	0.9310	0.9839
Yes							
2	17	1	0	0.9412	0.0571	0.6502	0.9915
29	16	1	0	0.8824	0.0781	0.6060	0.9692
76	15	1	0	0.8235	0.0925	0.5471	0.9394
90	14	0	14	0.8235	0.0925	0.5471	0.9394

```
150 . sts test  Confu

         failure _d:  MortGlo == 1
   analysis time _t:  TSup
```

Log-rank test for equality of survivor functions

Confu	Events observed	Events expected
No	7	9.31
Yes	3	0.69
Total	10	10.00

```
            chi2(1) =       8.22
            Pr>chi2 =     0.0041
```

```
151 . by MortGlo, sort: tabulate Confu
```

-> MortGlo = No

Confusión	Freq.	Percent	Cum.
No	202	93.52	93.52
Yes	14	6.48	100.00
Total	216	100.00	

-> MortGlo = Yes

Confusión	Freq.	Percent	Cum.
No	7	70.00	70.00
Yes	3	30.00	100.00
Total	10	100.00	

152 . sts, by(FRM30) risktable

failure _d: **MortGlo == 1**
analysis time _t: **TSup**

153 . sts list, by(FRM30)

failure _d: **MortGlo == 1**
analysis time _t: **TSup**

Time	Beg. Total	Fail	Net Lost	Survivor Function	Std. Error	[95% Conf.	Int.]
No							
3	216	1	0	0.9954	0.0046	0.9676	0.9993
5	215	1	0	0.9907	0.0065	0.9635	0.9977
29	214	1	0	0.9861	0.0080	0.9576	0.9955
31	213	1	0	0.9815	0.0092	0.9514	0.9930
45	212	1	0	0.9769	0.0102	0.9453	0.9903
56	211	1	0	0.9722	0.0112	0.9392	0.9874
88	210	1	0	0.9676	0.0120	0.9332	0.9844
90	209	0	209	0.9676	0.0120	0.9332	0.9844
Yes							
2	10	1	0	0.9000	0.0949	0.4730	0.9853
37	9	1	0	0.8000	0.1265	0.4087	0.9459
76	8	1	0	0.7000	0.1449	0.3287	0.8919
90	7	0	7	0.7000	0.1449	0.3287	0.8919

154 . sts test FRM30

failure _d: **MortGlo == 1**
analysis time _t: **TSup**

Log-rank test for equality of survivor functions

FRM30	Events observed	Events expected
No	7	9.61
Yes	3	0.39
Total	10	10.00

chi2(1) = 18.30
Pr>chi2 = 0.0000

155 . by MortGlo, sort: tabulate FRM30

-> MortGlo = No

Respiratory rate > 30 bpm	Freq.	Percent	Cum.
No	209	96.76	96.76
Yes	7	3.24	100.00
Total	216	100.00	

-> MortGlo = Yes

Respiratory rate > 30 bpm	Freq.	Percent	Cum.
No	7	70.00	70.00
Yes	3	30.00	100.00
Total	10	100.00	

```
156 .
157 . generate RxExt2 = .
    (226 missing values generated)

158 . replace RxExt2=0 if RxExt==0
    (173 real changes made)

159 . replace RxExt2=1 if RxExt>0
    (53 real changes made)

160 . label define dRxExt2 0 "Unilobar" 1 "Multilobar", replace

161 . label values RxExt2 dRxExt2

162 . label variable RxExt2 "Rx extension"

163 . sts, by(RxExt2) risktable

         failure _d:  MortGlo == 1
   analysis time _t:  TSup

164 . sts list, by(RxExt2)

         failure _d:  MortGlo == 1
   analysis time _t:  TSup
```

Time	Beg. Total	Fail	Net Lost	Survivor Function	Std. Error	[95% Conf.	Int.]
Unilobar							
2	173	1	0	0.9942	0.0058	0.9597	0.9992
3	172	1	0	0.9884	0.0081	0.9546	0.9971
31	171	1	0	0.9827	0.0099	0.9472	0.9944
45	170	1	0	0.9769	0.0114	0.9396	0.9913
56	169	1	0	0.9711	0.0127	0.9320	0.9879
76	168	1	0	0.9653	0.0139	0.9244	0.9843
90	167	0	167	0.9653	0.0139	0.9244	0.9843
Multilobar							
5	53	1	0	0.9811	0.0187	0.8735	0.9973
29	52	1	0	0.9623	0.0262	0.8574	0.9904
37	51	1	0	0.9434	0.0317	0.8347	0.9814
88	50	1	0	0.9245	0.0363	0.8113	0.9710
90	49	0	49	0.9245	0.0363	0.8113	0.9710

```
165 . sts test RxExt2

         failure _d:  MortGlo == 1
   analysis time _t:  TSup
```

Log-rank test for equality of survivor functions

RxExt2	Events observed	Events expected
Unilobar	6	7.68
Multilobar	4	2.32
Total	10	10.00

chi2(1) = 1.59
Pr>chi2 = 0.2067

166 . by MortGlo, sort: tabulate RxExt2

-> MortGlo = No

Rx extension	Freq.	Percent	Cum.
Unilobar	167	77.31	77.31
Multilobar	49	22.69	100.00
Total	216	100.00	

-> MortGlo = Yes

Rx extension	Freq.	Percent	Cum.
Unilobar	6	60.00	60.00
Multilobar	4	40.00	100.00
Total	10	100.00	

167 . sts, by(Derrame) risktable

failure _d: **MortGlo == 1**
analysis time _t: **TSup**

168 . sts list, by(Derrame)

failure _d: **MortGlo == 1**
analysis time _t: **TSup**

Time	Beg. Total	Fail	Net Lost	Survivor Function	Std. Error	[95% Conf.	Int.]
No							
2	206	1	0	0.9951	0.0048	0.9660	0.9993
3	205	1	0	0.9903	0.0068	0.9617	0.9976
5	204	1	0	0.9854	0.0083	0.9555	0.9953
29	203	1	0	0.9806	0.0096	0.9491	0.9927
31	202	1	0	0.9757	0.0107	0.9427	0.9898
45	201	1	0	0.9709	0.0117	0.9363	0.9868
76	200	1	0	0.9660	0.0126	0.9300	0.9837
88	199	1	0	0.9612	0.0135	0.9238	0.9804
90	198	0	198	0.9612	0.0135	0.9238	0.9804
Yes							
37	20	1	0	0.9500	0.0487	0.6947	0.9928
56	19	1	0	0.9000	0.0671	0.6560	0.9740
90	18	0	18	0.9000	0.0671	0.6560	0.9740

```
169 . sts test Derrame

         failure _d:  MortGlo == 1
   analysis time _t:  TSup


Log-rank test for equality of survivor functions

        |   Events         Events
Derrame |  observed       expected
--------+-------------------------
No      |         8           9.12
Yes     |         2           0.88
--------+-------------------------
Total   |        10          10.00

              chi2(1) =       1.58
              Pr>chi2 =     0.2084

170 . by MortGlo, sort: tabulate Derrame

-------------------------------------------------------------------------------------------------------------
-> MortGlo = No

Rx effusion |      Freq.     Percent        Cum.
------------+-----------------------------------
         No |        198       91.67       91.67
        Yes |         18        8.33      100.00
------------+-----------------------------------
      Total |        216      100.00

-------------------------------------------------------------------------------------------------------------
-> MortGlo = Yes

Rx effusion |      Freq.     Percent        Cum.
------------+-----------------------------------
         No |          8       80.00       80.00
        Yes |          2       20.00      100.00
------------+-----------------------------------
      Total |         10      100.00


171 .
172 . stcox Urea

         failure _d:  MortGlo == 1
   analysis time _t:  TSup

Iteration 0:   log likelihood = -54.003385
Iteration 1:   log likelihood = -46.505582
Iteration 2:   log likelihood = -46.497048
Iteration 3:   log likelihood = -46.497048
Refining estimates:
Iteration 0:   log likelihood = -46.497048

Cox regression -- no ties

No. of subjects =          226                     Number of obs    =       226
No. of failures =           10
Time at risk    =        19812
                                                   LR chi2(1)       =     15.01
Log likelihood  =   -46.497048                     Prob > chi2      =    0.0001

------------------------------------------------------------------------------
          _t | Haz. Ratio   Std. Err.      z    P>|z|     [95% Conf. Interval]
-------------+----------------------------------------------------------------
        Urea |   1.030879   .0071064     4.41   0.000     1.017044    1.044902
------------------------------------------------------------------------------
```

```
173 . estat phtest

      Test of proportional-hazards assumption

      Time:  Time
```

	chi2	df	Prob>chi2
global test	0.01	1	0.9245

```
174 . by MortGlo, sort : tabstat Urea, statistics( median iqr )

-> MortGlo = No
```

variable	p50	iqr
Urea	36	27

```
-> MortGlo = Yes
```

variable	p50	iqr
Urea	69	94

```
175 . stcox Na

         failure _d:  MortGlo == 1
   analysis time _t:  TSup

Iteration 0:   log likelihood = -53.958128
Iteration 1:   log likelihood = -52.804428
Iteration 2:   log likelihood = -52.686251
Iteration 3:   log likelihood = -52.685815
Refining estimates:
Iteration 0:   log likelihood = -52.685815

Cox regression -- no ties

No. of subjects =          225                   Number of obs   =        225
No. of failures =           10
Time at risk    =        19722
                                                 LR chi2(1)      =       2.54
Log likelihood  =   -52.685815                   Prob > chi2     =     0.1107
```

_t	Haz. Ratio	Std. Err.	z	P>|z|	[95% Conf. Interval]	
Na	1.140652	.101604	1.48	0.140	.9579265	1.358233

```
176 . estat phtest

      Test of proportional-hazards assumption

      Time:  Time
```

	chi2	df	Prob>chi2
global test	0.24	1	0.6243

```
177 . by MortGlo, sort : tabstat Na, statistics( median iqr )

-------------------------------------------------------------------------------
-> MortGlo = No

    variable |       p50       iqr
-------------+--------------------
          Na |       137         5
----------------------------------

-------------------------------------------------------------------------------
-> MortGlo = Yes

    variable |       p50       iqr
-------------+--------------------
          Na |       137         7
----------------------------------

178 . stcox pO2

         failure _d:  MortGlo == 1
   analysis time _t:  TSup

Iteration 0:   log likelihood = -44.343707
Iteration 1:   log likelihood =   -44.3263
Iteration 2:   log likelihood =  -44.32629
Refining estimates:
Iteration 0:   log likelihood =  -44.32629

Cox regression -- no ties

No. of subjects =          142                 Number of obs   =        142
No. of failures =            9
Time at risk    =        12305
                                               LR chi2(1)      =       0.03
Log likelihood  =    -44.32629                 Prob > chi2     =     0.8519

------------------------------------------------------------------------------
          _t | Haz. Ratio   Std. Err.      z    P>|z|     [95% Conf. Interval]
-------------+----------------------------------------------------------------
         pO2 |   .9948926   .0275025    -0.19   0.853     .9424229    1.050284
------------------------------------------------------------------------------

179 . estat phtest

      Test of proportional-hazards assumption

      Time:  Time
      ----------------------------------------------------------------
                  |                     chi2       df       Prob>chi2
      ------------+---------------------------------------------------
      global test |                     2.83        1         0.0926
      ----------------------------------------------------------------

180 . by MortGlo, sort : tabstat pO2, statistics( median iqr )

-------------------------------------------------------------------------------
-> MortGlo = No

    variable |       p50       iqr
-------------+--------------------
         pO2 |        62        14
----------------------------------

-------------------------------------------------------------------------------
-> MortGlo = Yes

    variable |       p50       iqr
-------------+--------------------
         pO2 |        58        21
----------------------------------
```

```
181 . stcox pH

         failure _d:  MortGlo == 1
   analysis time _t:  TSup

Iteration 0:   log likelihood = -52.495385
Iteration 1:   log likelihood = -48.769494
Iteration 2:   log likelihood = -48.704388
Iteration 3:   log likelihood = -48.704124
Refining estimates:
Iteration 0:   log likelihood = -48.704124

Cox regression -- no ties

No. of subjects =          195                     Number of obs   =       195
No. of failures =           10
Time at risk    =        17022
                                                   LR chi2(1)      =      7.58
Log likelihood  =   -48.704124                     Prob > chi2     =    0.0059
```

_t	Haz. Ratio	Std. Err.	z	P>|z|	[95% Conf. Interval]	
pH	.0000784	.0002275	-3.26	0.001	2.65e-07	.0231899

```
182 . estat phtest

      Test of proportional-hazards assumption

      Time:  Time
```

	chi2	df	Prob>chi2
global test	0.88	1	0.3469

```
183 . by MortGlo, sort : tabstat pH, statistics( median iqr )

-> MortGlo = No
```

variable	p50	iqr
pH	7.44	.07

```
-> MortGlo = Yes
```

variable	p50	iqr
pH	7.34	.16

```
184 . stcox Lactico

         failure _d:  MortGlo == 1
   analysis time _t:  TSup

Iteration 0:   log likelihood = -42.934006
Iteration 1:   log likelihood = -38.568857
Iteration 2:   log likelihood = -38.283698
Iteration 3:   log likelihood =  -38.28335
Refining estimates:
Iteration 0:   log likelihood =  -38.28335

Cox regression -- no ties
```

```
No. of subjects =          122                  Number of obs    =       122
No. of failures =            9
Time at risk    =        10505
                                                LR chi2(1)       =      9.30
Log likelihood  =    -38.28335                  Prob > chi2      =    0.0023
```

_t	Haz. Ratio	Std. Err.	z	P>|z|	[95% Conf.	Interval]
Lactico	1.962211	.3575349	3.70	0.000	1.372929	2.804422

185 . estat phtest

Test of proportional-hazards assumption

Time: **Time**

	chi2	df	Prob>chi2
global test	0.58	1	0.4472

186 . by MortGlo, sort : tabstat Lactico, statistics(median iqr)

-> MortGlo = No

variable	p50	iqr
Lactico	1.5	1

-> MortGlo = Yes

variable	p50	iqr
Lactico	1.7	3

187 . stcox Leucocitos

```
         failure _d:  MortGlo == 1
   analysis time _t:  TSup

Iteration 0:   log likelihood = -54.003385
Iteration 1:   log likelihood = -53.455383
Iteration 2:   log likelihood = -53.437927
Iteration 3:   log likelihood = -53.437919
Refining estimates:
Iteration 0:   log likelihood = -53.437919

Cox regression -- no ties

No. of subjects =          226                  Number of obs    =       226
No. of failures =           10
Time at risk    =        19812
                                                LR chi2(1)       =      1.13
Log likelihood  =   -53.437919                  Prob > chi2      =    0.2876
```

_t	Haz. Ratio	Std. Err.	z	P>|z|	[95% Conf.	Interval]
Leucocitos	1.00005	.0000451	1.11	0.265	.9999619	1.000139

188 . estat phtest

Test of proportional-hazards assumption

Time: **Time**

	chi2	df	Prob>chi2
global test	0.00	1	0.9962

189 . by MortGlo, sort : tabstat Leucocitos, statistics(median iqr)

-> MortGlo = No

variable	p50	iqr
Leucocitos	12100	7800

-> MortGlo = Yes

variable	p50	iqr
Leucocitos	15050	6800

190 . stcox Glu

```
         failure _d:  MortGlo == 1
   analysis time _t:  TSup

Iteration 0:   log likelihood = -53.775025
Iteration 1:   log likelihood = -53.774309
Iteration 2:   log likelihood = -53.774309
Refining estimates:
Iteration 0:   log likelihood = -53.774309

Cox regression -- no ties

No. of subjects =          221                 Number of obs   =        221
No. of failures =           10
Time at risk    =        19362
                                               LR chi2(1)      =       0.00
Log likelihood  =   -53.774309                 Prob > chi2     =     0.9698
```

_t	Haz. Ratio	Std. Err.	z	P>|z|	[95% Conf. Interval]	
Glu	.9998183	.0048263	-0.04	0.970	.9904035	1.009323

191 . estat phtest

Test of proportional-hazards assumption

Time: **Time**

	chi2	df	Prob>chi2
global test	0.88	1	0.3494

```
192 . by MortGlo, sort : tabstat Glu, statistics( median iqr )

-> MortGlo = No

    variable |       p50       iqr
-------------+--------------------
         Glu |       117        54
----------------------------------

-> MortGlo = Yes

    variable |       p50       iqr
-------------+--------------------
         Glu |       129        49
----------------------------------

193 . stcox Hcto

         failure _d:  MortGlo == 1
   analysis time _t:  TSup

Iteration 0:   log likelihood = -54.003385
Iteration 1:   log likelihood = -53.317535
Iteration 2:   log likelihood = -53.280608
Iteration 3:   log likelihood = -53.280438
Refining estimates:
Iteration 0:   log likelihood = -53.280438

Cox regression -- no ties

No. of subjects =          226                     Number of obs   =       226
No. of failures =           10
Time at risk    =        19812
                                                   LR chi2(1)      =      1.45
Log likelihood  =   -53.280438                     Prob > chi2     =    0.2292

------------------------------------------------------------------------------
          _t | Haz. Ratio   Std. Err.      z    P>|z|     [95% Conf. Interval]
-------------+----------------------------------------------------------------
        Hcto |   .9332723   .0490864    -1.31   0.189     .8418574    1.034614
------------------------------------------------------------------------------

194 . estat phtest

      Test of proportional-hazards assumption

      Time:  Time
      ----------------------------------------------------------------
                  |                 chi2       df       Prob>chi2
      ------------+---------------------------------------------------
      global test |                 2.40        1         0.1213
      ----------------------------------------------------------------

195 . by MortGlo, sort : tabstat Hcto, statistics( median iqr )

-> MortGlo = No

    variable |       p50       iqr
-------------+--------------------
        Hcto |      39.7       5.4
----------------------------------

-> MortGlo = Yes
```

variable	p50	iqr
Hcto	37.2	8.8

```
196 . stcox PCR

         failure _d:  MortGlo == 1
   analysis time _t:  TSup

Iteration 0:   log likelihood = -53.912666
Iteration 1:   log likelihood = -53.813844
Iteration 2:   log likelihood = -53.813538
Iteration 3:   log likelihood = -53.813538
Refining estimates:
Iteration 0:   log likelihood = -53.813538

Cox regression -- no ties

No. of subjects =          224                   Number of obs   =        224
No. of failures =           10
Time at risk    =        19632
                                                 LR chi2(1)      =       0.20
Log likelihood  =   -53.813538                   Prob > chi2     =     0.6561
```

_t	Haz. Ratio	Std. Err.	z	P>|z|	[95% Conf.	Interval]
PCR	1.01323	.0294327	0.45	0.651	.9571541	1.07259

```
197 . estat phtest

      Test of proportional-hazards assumption

      Time:  Time
```

	chi2	df	Prob>chi2
global test	2.80	1	0.0942

```
198 . by MortGlo, sort : tabstat PCR, statistics( median iqr )
```

-> MortGlo = No

variable	p50	iqr
PCR	9.25	15.2

-> MortGlo = Yes

variable	p50	iqr
PCR	6.75	18.9

```
199 . stcox ProADM

         failure _d:  MortGlo == 1
   analysis time _t:  TSup

Iteration 0:   log likelihood = -54.003385
Iteration 1:   log likelihood = -45.363774
Iteration 2:   log likelihood = -45.013066
Iteration 3:   log likelihood = -39.980036
Iteration 4:   log likelihood = -39.766014
Refining estimates:
Iteration 0:   log likelihood = -39.766014

Cox regression -- no ties

No. of subjects =          226                     Number of obs   =       226
No. of failures =           10
Time at risk    =        19812
                                                   LR chi2(1)      =     28.47
Log likelihood  =   -39.766014                     Prob > chi2     =    0.0000
```

_t	Haz. Ratio	Std. Err.	z	P>|z|	[95% Conf.	Interval]
ProADM	2.695595	.5613562	4.76	0.000	1.792225	4.054309

```
200 . estat phtest

      Test of proportional-hazards assumption

      Time:  Time
```

	chi2	df	Prob>chi2
global test	0.34	1	0.5577

```
201 . by MortGlo, sort : tabstat ProADM, statistics( median iqr )

-> MortGlo = No
```

variable	p50	iqr
ProADM	1.06	.785

```
-> MortGlo = Yes
```

variable	p50	iqr
ProADM	3.145	2.47

```
202 . stcox PCT

         failure _d:  MortGlo == 1
   analysis time _t:  TSup

Iteration 0:   log likelihood = -53.912666
Iteration 1:   log likelihood = -53.796861
Iteration 2:   log likelihood = -53.728592
Iteration 3:   log likelihood = -53.726589
Iteration 4:   log likelihood = -53.726587
Refining estimates:
Iteration 0:   log likelihood = -53.726587

Cox regression -- no ties
```

```
No. of subjects =          224                  Number of obs   =        224
No. of failures =           10
Time at risk    =        19632
                                                LR chi2(1)      =       0.37
Log likelihood  =   -53.726587                  Prob > chi2     =     0.5418
```

_t	Haz. Ratio	Std. Err.	z	P>|z|	[95% Conf.	Interval]
PCT	1.056314	.083796	0.69	0.490	.9042077	1.234007

203 . estat phtest

Test of proportional-hazards assumption

Time: **Time**

	chi2	df	Prob>chi2
global test	0.91	1	0.3405

204 . by MortGlo, sort : tabstat PCT, statistics(median iqr)

-> MortGlo = No

variable	p50	iqr
PCT	.125	.43

-> MortGlo = Yes

variable	p50	iqr
PCT	.37	2.36

205 . stcox ProBNP

```
         failure _d:  MortGlo == 1
   analysis time _t:  TSup

Iteration 0:   log likelihood = -53.958128
Iteration 1:   log likelihood = -53.686362
Iteration 2:   log likelihood = -51.798399
Iteration 3:   log likelihood = -51.686822
Iteration 4:   log likelihood =  -51.68506
Refining estimates:
Iteration 0:   log likelihood =  -51.68506

Cox regression -- no ties

No. of subjects =          225                  Number of obs   =        225
No. of failures =           10
Time at risk    =        19722
                                                LR chi2(1)      =       4.55
Log likelihood  =    -51.68506                  Prob > chi2     =     0.0330
```

_t	Haz. Ratio	Std. Err.	z	P>|z|	[95% Conf.	Interval]
ProBNP	1.000104	.0000367	2.85	0.004	1.000033	1.000176

```
206 . estat phtest

      Test of proportional-hazards assumption

      Time:  Time
      ----------------------------------------------------------------
                   |       chi2       df       Prob>chi2
      -------------+--------------------------------------------------
      global test  |       0.11        1         0.7408
      ----------------------------------------------------------------

207 . by MortGlo, sort : tabstat ProBNP, statistics( median iqr )

-------------------------------------------------------------------------------------------------
-> MortGlo = No

    variable |       p50       iqr
-------------+--------------------
      ProBNP |       460      1427
----------------------------------

-------------------------------------------------------------------------------------------------
-> MortGlo = Yes

    variable |       p50       iqr
-------------+--------------------
      ProBNP |      2572      6130
----------------------------------

208 . stcox PSI

         failure _d:  MortGlo == 1
   analysis time _t:  TSup

Iteration 0:   log likelihood = -54.003385
Iteration 1:   log likelihood = -47.807809
Iteration 2:   log likelihood = -46.260656
Iteration 3:   log likelihood = -46.247533
Refining estimates:
Iteration 0:   log likelihood = -46.247533

Cox regression -- no ties

No. of subjects =          226                    Number of obs   =        226
No. of failures =           10
Time at risk    =        19812
                                                  LR chi2(1)      =      15.51
Log likelihood  =   -46.247533                    Prob > chi2     =     0.0001

------------------------------------------------------------------------------
          _t | Haz. Ratio   Std. Err.      z    P>|z|     [95% Conf. Interval]
-------------+----------------------------------------------------------------
         PSI |   1.025817   .0062398     4.19   0.000      1.01366     1.03812
------------------------------------------------------------------------------

209 . estat phtest

      Test of proportional-hazards assumption

      Time:  Time
      ----------------------------------------------------------------
                   |       chi2       df       Prob>chi2
      -------------+--------------------------------------------------
      global test  |       0.07        1         0.7980
      ----------------------------------------------------------------
```

```
210 . by MortGlo, sort : tabstat PSI, statistics( median iqr )
```

-> MortGlo = No

variable	p50	iqr
PSI	82	47.5

-> MortGlo = Yes

variable	p50	iqr
PSI	145.5	45

```
211 . sts, by(PSI3) risktable

         failure _d:  MortGlo == 1
   analysis time _t:  TSup

212 . sts list, by(PSI3)

         failure _d:  MortGlo == 1
   analysis time _t:  TSup
```

Time	Beg. Total	Fail	Net Lost	Survivor Function	Std. Error	[95% Conf.	Int.]
Mild							
90	137	0	137	1.0000	.	.	.
Moderate							
29	58	1	0	0.9828	0.0171	0.8838	0.9976
31	57	1	0	0.9655	0.0240	0.8691	0.9913
45	56	1	0	0.9483	0.0291	0.8482	0.9830
90	55	0	55	0.9483	0.0291	0.8482	0.9830
Severe							
2	31	1	0	0.9677	0.0317	0.7923	0.9954
3	30	1	0	0.9355	0.0441	0.7659	0.9835
5	29	1	0	0.9032	0.0531	0.7293	0.9677
37	28	1	0	0.8710	0.0602	0.6919	0.9495
56	27	1	0	0.8387	0.0661	0.6550	0.9295
76	26	1	0	0.8065	0.0710	0.6191	0.9080
88	25	1	0	0.7742	0.0751	0.5840	0.8854
90	24	0	24	0.7742	0.0751	0.5840	0.8854

```
213 . sts test  PSI3

         failure _d:  MortGlo == 1
   analysis time _t:  TSup
```

Log-rank test for equality of survivor functions

PSI3	Events observed	Events expected
Mild	0	6.19
Moderate	3	2.56
Severe	7	1.26
Total	10	10.00

```
          chi2(2) =      32.46
          Pr>chi2 =     0.0000
```

```
214 . by MortGlo, sort : tabulate PSI3
```

-> MortGlo = No

PSI 3 categorias	Freq.	Percent	Cum.
Mild	137	63.43	63.43
Moderate	55	25.46	88.89
Severe	24	11.11	100.00
Total	216	100.00	

-> MortGlo = Yes

PSI 3 categorias	Freq.	Percent	Cum.
Moderate	3	30.00	30.00
Severe	7	70.00	100.00
Total	10	100.00	

```
215 . sts, by(HCP) risktable

         failure _d:  MortGlo == 1
   analysis time _t:  TSup

216 . sts list, by(HCP)

         failure _d:  MortGlo == 1
   analysis time _t:  TSup
```

Time	Beg. Total	Fail	Net Lost	Survivor Function	Std. Error	[95% Conf.	Int.]
No							
5	169	1	0	0.9941	0.0059	0.9587	0.9992
29	168	1	0	0.9882	0.0083	0.9535	0.9970
31	167	1	0	0.9822	0.0102	0.9460	0.9942
37	166	1	0	0.9763	0.0117	0.9382	0.9911
56	165	1	0	0.9704	0.0130	0.9304	0.9876
76	164	1	0	0.9645	0.0142	0.9227	0.9839
88	163	1	0	0.9586	0.0153	0.9151	0.9800
90	162	0	162	0.9586	0.0153	0.9151	0.9800
Yes							
90	6	0	6	1.0000	.	.	.

```
217 . sts test HCP

         failure _d:  MortGlo == 1
   analysis time _t:  TSup
```

Log-rank test for equality of survivor functions

HCP	Events observed	Events expected
No	7	6.76
Yes	0	0.24
Total	7	7.00

```
            chi2(1) =       0.25
            Pr>chi2 =     0.6149
```

218 . by MortGlo, sort : tabulate HCP

-> MortGlo = No

Bacteremia	Freq.	Percent	Cum.
No	162	96.43	96.43
Yes	6	3.57	100.00
Total	168	100.00	

-> MortGlo = Yes

Bacteremia	Freq.	Percent	Cum.
No	7	100.00	100.00
Total	7	100.00	

219 . sts, by(Destino) risktable

failure _d: **MortGlo == 1**
analysis time _t: **TSup**

220 . sts list, by(Destino)

failure _d: **MortGlo == 1**
analysis time _t: **TSup**

Time	Beg. Total	Fail	Net Lost	Survivor Function	Std. Error	[95% Conf.	Int.]
Discharge							
90	43	0	43	1.0000	.	.	.
Admission							
2	183	1	0	0.9945	0.0054	0.9618	0.9992
3	182	1	0	0.9891	0.0077	0.9570	0.9973
5	181	1	0	0.9836	0.0094	0.9500	0.9947
29	180	1	0	0.9781	0.0108	0.9428	0.9917
31	179	1	0	0.9727	0.0121	0.9356	0.9885
37	178	1	0	0.9672	0.0132	0.9285	0.9851
45	177	1	0	0.9617	0.0142	0.9214	0.9816
56	176	1	0	0.9563	0.0151	0.9145	0.9779
76	175	1	0	0.9508	0.0160	0.9076	0.9741
88	174	1	0	0.9454	0.0168	0.9008	0.9702
90	173	0	173	0.9454	0.0168	0.9008	0.9702

221 . sts test Destino

failure _d: **MortGlo == 1**
analysis time _t: **TSup**

Log-rank test for equality of survivor functions

Destino	Events observed	Events expected
Discharge	0	1.94
Admission	10	8.06
Total	10	10.00

chi2(1) = 2.41
Pr>chi2 = 0.1206

```
222 . by MortGlo, sort : tabulate Destino

-> MortGlo = No

  Treatment |
       site |      Freq.     Percent        Cum.
------------+-----------------------------------
  Discharge |         43       19.91       19.91
  Admission |        173       80.09      100.00
------------+-----------------------------------
      Total |        216      100.00

-> MortGlo = Yes

  Treatment |
       site |      Freq.     Percent        Cum.
------------+-----------------------------------
  Admission |         10      100.00      100.00
------------+-----------------------------------
      Total |         10      100.00

223 .
224 . preserve

225 . summarize ProADM, detail

                        ProADM (nmol/L)
-------------------------------------------------------------
      Percentiles      Smallest
 1%          .35            .33
 5%          .43            .34
10%          .52            .35       Obs                 226
25%          .75            .36       Sum of Wgt.         226

50%        1.075                      Mean           1.369513
                        Largest       Std. Dev.       1.20381
75%         1.55           4.68
90%         2.42           4.75       Variance       1.449158
95%         3.29           5.14       Skewness       5.399021
99%         4.75           13.6       Kurtosis       49.65381

226 . recode ProADM (min/0.75 = 0 ) (0.75/1.075= 1) (1.075/1.55= 2) (1.55/max= 3)
    (ProADM: 226 changes made)

227 . label define dCuartiles 0 "1° cuartil" 1 "2° cuartil" 2 "3° cuartil" 3 "4° cuartil"

228 . label values ProADM dCuartiles

229 . sts, by(ProADM) risktable

         failure _d:  MortGlo == 1
   analysis time _t:  TSup

230 . sts list, by(ProADM)

         failure _d:  MortGlo == 1
   analysis time _t:  TSup
```

Time	Beg. Total	Fail	Net Lost	Survivor Function	Std. Error	[95% Conf. Int.]	
1° cuartil							
90	57	0	57	1.0000	.	.	.
2° cuartil							
90	56	0	56	1.0000	.	.	.
3° cuartil							
29	57	1	0	0.9825	0.0174	0.8819	0.9975
31	56	1	0	0.9649	0.0244	0.8669	0.9911
90	55	0	55	0.9649	0.0244	0.8669	0.9911
4° cuartil							
2	56	1	0	0.9821	0.0177	0.8799	0.9975
3	55	1	0	0.9643	0.0248	0.8647	0.9909
5	54	1	0	0.9464	0.0301	0.8430	0.9824
37	53	1	0	0.9286	0.0344	0.8208	0.9726
45	52	1	0	0.9107	0.0381	0.7987	0.9618
56	51	1	0	0.8929	0.0413	0.7770	0.9504
76	50	1	0	0.8750	0.0442	0.7556	0.9384
88	49	1	0	0.8571	0.0468	0.7346	0.9259
90	48	0	48	0.8571	0.0468	0.7346	0.9259

231 . sts test ProADM

```
         failure _d:  MortGlo == 1
   analysis time _t:  TSup
```

Log-rank test for equality of survivor functions

ProADM	Events observed	Events expected
1° cuartil	0	2.57
2° cuartil	0	2.53
3° cuartil	2	2.52
4° cuartil	8	2.37
Total	10	10.00

chi2(3) = 18.55
Pr>chi2 = 0.0003

232 . restore

233 .
234 . roctab MortGlo ProADM, detail graph

Detailed report of sensitivity and specificity

Cutpoint	Sensitivity	Specificity	Correctly Classified	LR+	LR-
(>= .33)	100.00%	0.00%	4.42%	1.0000	
(>= .34)	100.00%	0.46%	4.87%	1.0047	0.0000
(>= .35)	100.00%	0.93%	5.31%	1.0093	0.0000
(>= .36)	100.00%	1.39%	5.75%	1.0141	0.0000
(>= .37)	100.00%	1.85%	6.19%	1.0189	0.0000
(>= .4)	100.00%	2.31%	6.64%	1.0237	0.0000
(>= .41)	100.00%	3.24%	7.52%	1.0335	0.0000
(>= .42)	100.00%	3.70%	7.96%	1.0385	0.0000
(>= .43)	100.00%	5.09%	9.29%	1.0537	0.0000
(>= .44)	100.00%	6.02%	10.18%	1.0640	0.0000
(>= .45)	100.00%	6.94%	11.06%	1.0746	0.0000
(>= .48)	100.00%	7.87%	11.95%	1.0854	0.0000
(>= .49)	100.00%	8.33%	12.39%	1.0909	0.0000
(>= .51)	100.00%	8.80%	12.83%	1.0964	0.0000
(>= .52)	100.00%	9.72%	13.72%	1.1077	0.0000
(>= .53)	100.00%	10.65%	14.60%	1.1192	0.0000
(>= .55)	100.00%	11.57%	15.49%	1.1309	0.0000
(>= .56)	100.00%	12.50%	16.37%	1.1429	0.0000

(>= .57)	100.00%	13.43%	17.26%	1.1551	0.0000
(>= .58)	100.00%	14.35%	18.14%	1.1676	0.0000
(>= .61)	100.00%	15.28%	19.03%	1.1803	0.0000
(>= .62)	100.00%	16.67%	20.35%	1.2000	0.0000
(>= .63)	100.00%	18.06%	21.68%	1.2203	0.0000
(>= .64)	100.00%	18.98%	22.57%	1.2343	0.0000
(>= .66)	100.00%	19.44%	23.01%	1.2414	0.0000
(>= .67)	100.00%	20.37%	23.89%	1.2558	0.0000
(>= .68)	100.00%	20.83%	24.34%	1.2632	0.0000
(>= .7)	100.00%	22.22%	25.66%	1.2857	0.0000
(>= .71)	100.00%	23.61%	26.99%	1.3091	0.0000
(>= .72)	100.00%	24.54%	27.88%	1.3252	0.0000
(>= .73)	100.00%	25.00%	28.32%	1.3333	0.0000
(>= .74)	100.00%	25.46%	28.76%	1.3416	0.0000
(>= .75)	100.00%	25.93%	29.20%	1.3500	0.0000
(>= .77)	100.00%	26.39%	29.65%	1.3585	0.0000
(>= .78)	100.00%	26.85%	30.09%	1.3671	0.0000
(>= .79)	100.00%	27.31%	30.53%	1.3758	0.0000
(>= .8)	100.00%	28.24%	31.42%	1.3935	0.0000
(>= .82)	100.00%	30.56%	33.63%	1.4400	0.0000
(>= .83)	100.00%	31.48%	34.51%	1.4595	0.0000
(>= .84)	100.00%	31.94%	34.96%	1.4694	0.0000
(>= .85)	100.00%	32.41%	35.40%	1.4795	0.0000
(>= .86)	100.00%	35.65%	38.50%	1.5540	0.0000
(>= .87)	100.00%	36.11%	38.94%	1.5652	0.0000
(>= .88)	100.00%	37.04%	39.82%	1.5882	0.0000
(>= .89)	100.00%	37.50%	40.27%	1.6000	0.0000
(>= .9)	100.00%	37.96%	40.71%	1.6119	0.0000
(>= .91)	100.00%	39.35%	42.04%	1.6489	0.0000
(>= .92)	100.00%	40.28%	42.92%	1.6744	0.0000
(>= .93)	100.00%	40.74%	43.36%	1.6875	0.0000
(>= .96)	100.00%	41.20%	43.81%	1.7008	0.0000
(>= .97)	100.00%	41.67%	44.25%	1.7143	0.0000
(>= .98)	100.00%	42.59%	45.13%	1.7419	0.0000
(>= .99)	100.00%	43.52%	46.02%	1.7705	0.0000
(>= 1.02)	100.00%	44.44%	46.90%	1.8000	0.0000
(>= 1.03)	100.00%	45.83%	48.23%	1.8462	0.0000
(>= 1.04)	100.00%	47.69%	50.00%	1.9115	0.0000
(>= 1.05)	100.00%	48.61%	50.88%	1.9459	0.0000
(>= 1.06)	100.00%	49.54%	51.77%	1.9817	0.0000
(>= 1.07)	100.00%	50.93%	53.10%	2.0377	0.0000
(>= 1.08)	100.00%	52.31%	54.42%	2.0971	0.0000
(>= 1.09)	100.00%	52.78%	54.87%	2.1176	0.0000
(>= 1.1)	100.00%	53.70%	55.75%	2.1600	0.0000
(>= 1.11)	100.00%	54.63%	56.64%	2.2041	0.0000
(>= 1.12)	100.00%	55.56%	57.52%	2.2500	0.0000
(>= 1.14)	100.00%	56.02%	57.96%	2.2737	0.0000
(>= 1.15)	100.00%	56.94%	58.85%	2.3226	0.0000
(>= 1.17)	100.00%	57.41%	59.29%	2.3478	0.0000
(>= 1.18)	100.00%	57.87%	59.73%	2.3736	0.0000
(>= 1.19)	100.00%	58.80%	60.62%	2.4270	0.0000
(>= 1.2)	90.00%	59.26%	60.62%	2.2091	0.1688
(>= 1.21)	90.00%	60.19%	61.50%	2.2605	0.1662
(>= 1.22)	90.00%	60.65%	61.95%	2.2871	0.1649
(>= 1.23)	90.00%	61.11%	62.39%	2.3143	0.1636
(>= 1.26)	90.00%	61.57%	62.83%	2.3422	0.1624
(>= 1.28)	90.00%	62.04%	63.27%	2.3707	0.1612
(>= 1.29)	90.00%	62.96%	64.16%	2.4300	0.1588
(>= 1.3)	90.00%	63.43%	64.60%	2.4608	0.1577
(>= 1.31)	80.00%	65.28%	65.93%	2.3040	0.3064
(>= 1.32)	80.00%	65.74%	66.37%	2.3351	0.3042
(>= 1.33)	80.00%	66.67%	67.26%	2.4000	0.3000
(>= 1.34)	80.00%	67.13%	67.70%	2.4338	0.2979
(>= 1.35)	80.00%	68.06%	68.58%	2.5043	0.2939
(>= 1.36)	80.00%	68.52%	69.03%	2.5412	0.2919
(>= 1.38)	80.00%	68.98%	69.47%	2.5791	0.2899
(>= 1.39)	80.00%	69.91%	70.35%	2.6585	0.2861
(>= 1.42)	80.00%	70.37%	70.80%	2.7000	0.2842
(>= 1.43)	80.00%	70.83%	71.24%	2.7429	0.2824
(>= 1.44)	80.00%	71.76%	72.12%	2.8328	0.2787
(>= 1.45)	80.00%	72.69%	73.01%	2.9288	0.2752
(>= 1.47)	80.00%	73.15%	73.45%	2.9793	0.2734

(>= 1.5)	80.00%	74.07%	74.34%	3.0857	0.2700
(>= 1.51)	80.00%	74.54%	74.78%	3.1418	0.2683
(>= 1.53)	80.00%	75.93%	76.11%	3.3231	0.2634
(>= 1.54)	80.00%	76.39%	76.55%	3.3882	0.2618
(>= 1.55)	80.00%	76.85%	76.99%	3.4560	0.2602
(>= 1.56)	80.00%	77.78%	77.88%	3.6000	0.2571
(>= 1.57)	80.00%	78.70%	78.76%	3.7565	0.2541
(>= 1.59)	80.00%	79.17%	79.20%	3.8400	0.2526
(>= 1.6)	80.00%	80.56%	80.53%	4.1143	0.2483
(>= 1.61)	80.00%	81.48%	81.42%	4.3200	0.2455
(>= 1.65)	80.00%	81.94%	81.86%	4.4308	0.2441
(>= 1.67)	80.00%	82.41%	82.30%	4.5474	0.2427
(>= 1.69)	80.00%	83.33%	83.19%	4.8000	0.2400
(>= 1.7)	80.00%	83.80%	83.63%	4.9371	0.2387
(>= 1.71)	80.00%	84.26%	84.07%	5.0824	0.2374
(>= 1.74)	80.00%	84.72%	84.51%	5.2364	0.2361
(>= 1.8)	80.00%	85.19%	84.96%	5.4000	0.2348
(>= 1.82)	80.00%	85.65%	85.40%	5.5742	0.2335
(>= 1.89)	80.00%	86.57%	86.28%	5.9586	0.2310
(>= 1.97)	70.00%	86.57%	85.84%	5.2138	0.3465
(>= 2.03)	70.00%	87.04%	86.28%	5.4000	0.3447
(>= 2.06)	70.00%	87.50%	86.73%	5.6000	0.3429
(>= 2.12)	70.00%	87.96%	87.17%	5.8154	0.3411
(>= 2.19)	70.00%	88.43%	87.61%	6.0480	0.3393
(>= 2.22)	70.00%	88.89%	88.05%	6.3000	0.3375
(>= 2.25)	70.00%	89.35%	88.50%	6.5739	0.3358
(>= 2.27)	70.00%	89.81%	88.94%	6.8727	0.3340
(>= 2.29)	70.00%	90.28%	89.38%	7.2000	0.3323
(>= 2.31)	60.00%	90.28%	88.94%	6.1714	0.4431
(>= 2.35)	60.00%	90.74%	89.38%	6.4800	0.4408
(>= 2.37)	60.00%	91.20%	89.82%	6.8211	0.4386
(>= 2.39)	60.00%	91.67%	90.27%	7.2000	0.4364
(>= 2.42)	50.00%	91.67%	89.82%	6.0000	0.5455
(>= 2.44)	50.00%	92.59%	90.71%	6.7500	0.5400
(>= 2.5)	50.00%	93.06%	91.15%	7.2000	0.5373
(>= 2.55)	50.00%	93.52%	91.59%	7.7143	0.5347
(>= 2.66)	50.00%	94.44%	92.48%	9.0000	0.5294
(>= 2.72)	50.00%	94.91%	92.92%	9.8182	0.5268
(>= 2.99)	50.00%	95.37%	93.36%	10.8000	0.5243
(>= 3.23)	50.00%	96.30%	94.25%	13.5000	0.5192
(>= 3.29)	50.00%	96.76%	94.69%	15.4286	0.5167
(>= 3.3)	50.00%	97.22%	95.13%	18.0000	0.5143
(>= 3.86)	50.00%	97.69%	95.58%	21.6000	0.5118
(>= 3.9)	50.00%	98.15%	96.02%	27.0000	0.5094
(>= 4.24)	40.00%	98.15%	95.58%	21.6000	0.6113
(>= 4.36)	30.00%	98.15%	95.13%	16.2000	0.7132
(>= 4.5)	20.00%	98.15%	94.69%	10.8000	0.8151
(>= 4.52)	20.00%	98.61%	95.13%	14.4000	0.8113
(>= 4.68)	20.00%	99.07%	95.58%	21.6000	0.8075
(>= 4.75)	10.00%	99.07%	95.13%	10.8000	0.9084
(>= 5.14)	10.00%	99.54%	95.58%	21.6002	0.9042
(>= 13.6)	10.00%	100.00%	96.02%		0.9000
(> 13.6)	0.00%	100.00%	95.58%		1.0000

Obs	ROC Area	Std. Err.	—Asymptotic Normal— [95% Conf.	Interval]
226	0.8854	0.0486	0.79021	0.98062

```
235 . roctab  MortGlo PSI

                      ROC                    —Asymptotic Normal——
           Obs       Area     Std. Err.      [95% Conf. Interval]
         ----------------------------------------------------------
           226     0.8900       0.0357        0.82007     0.96003

236 . roccomp MortGlo PSI ProADM

                              ROC                    —Asymptotic Normal——
                   Obs       Area     Std. Err.      [95% Conf. Interval]
    -------------------------------------------------------------------------
    PSI            226     0.8900       0.0357        0.82007     0.96003
    ProADM         226     0.8854       0.0486        0.79021     0.98062
    -------------------------------------------------------------------------
    Ho: area(PSI) = area(ProADM)
        chi2(1) =     0.01       Prob>chi2 =   0.9034

237 . roctab  MortGlo ProBNP

                      ROC                    —Asymptotic Normal——
           Obs       Area     Std. Err.      [95% Conf. Interval]
         ----------------------------------------------------------
           225     0.7337       0.0821        0.57284     0.89460

238 . roctab  MortGlo PCT

                      ROC                    —Asymptotic Normal——
           Obs       Area     Std. Err.      [95% Conf. Interval]
         ----------------------------------------------------------
           224     0.7329       0.0584        0.61846     0.84743

239 . roctab  MortGlo PCR

                      ROC                    —Asymptotic Normal——
           Obs       Area     Std. Err.      [95% Conf. Interval]
         ----------------------------------------------------------
           224     0.4977       0.1041        0.29356     0.70177

240 .
241 . *Table 2
242 . allsets PSI  ProADM ProBNP, cox

    ALLSETS - Cox regression

    ALL VARIABLES
    Continuous: PSI ProADM ProBNP

        Variable |     Valid    Missing
    -------------+----------------------
             PSI |       226          0
          ProADM |       226          0
          ProBNP |       225          1
    -------------+----------------------
    Valid number of cases (listwise): 225
    Total number of hierarchical submodels estimated: 7
    Total time:  0.1 seconds

    A new dataset has been created with the results
    Execute use "allsets_results.dta" to open the dataset with the results

       stats |       AIC       BIC        R2       _2ll
    ---------+-----------------------------------------
         min |      80.4      83.8     .0236       78.3
         max |       105       109      .255        103
    ---------------------------------------------------
```

```
243 . save "C:\Users\susan_000\Documents\NACURG\NUEVO\NACURGPLOS",replace
    file C:\Users\susan_000\Documents\NACURG\NUEVO\NACURGPLOS.dta saved

244 . use "allsets_results.dta"

245 . list NVar- _2ll, clean noobs

        NVar   Variables              AIC      BIC        R2       _2ll
           1   ProADM                80.4     83.8    0.2550     78.395
           2   PSI ProADM            82.3     89.2    0.2372     78.319
           2   ProADM ProBNP         82.4     89.2    0.2365     78.395
           3   PSI ProADM ProBNP     84.3     94.6    0.2187     78.318
           1   PSI                   94.0     97.4    0.1292     91.973
           2   PSI ProBNP            95.3    102.1    0.1169     91.306
           1   ProBNP               105.4    108.8    0.0236    103.370

246 . use "C:\Users\susan_000\Documents\NACURG\NUEVO\NACURGPLOS.dta"
    (Estudio NACURG)

247 .
248 . stcox PSI ProADM ProBNP

             failure _d:  MortGlo == 1
       analysis time _t:  TSup

    Iteration 0:   log likelihood = -53.958128
    Iteration 1:   log likelihood = -48.525163
    Iteration 2:   log likelihood = -44.883301
    Iteration 3:   log likelihood = -39.489279
    Iteration 4:   log likelihood = -39.165966
    Iteration 5:   log likelihood = -39.158922
    Iteration 6:   log likelihood = -39.158914
    Refining estimates:
    Iteration 0:   log likelihood = -39.158914

    Cox regression -- no ties

    No. of subjects =          225                     Number of obs    =        225
    No. of failures =           10
    Time at risk    =        19722
                                                       LR chi2(3)       =      29.60
    Log likelihood  =   -39.158914                     Prob > chi2      =     0.0000
```

_t	Haz. Ratio	Std. Err.	z	P>|z|	[95% Conf.	Interval]
PSI	.9966081	.0121567	-0.28	0.781	.9730639	1.020722
ProADM	3.137542	1.469895	2.44	0.015	1.252603	7.85897
ProBNP	.9999979	.0000585	-0.04	0.971	.9998833	1.000112

```
249 . estat phtest,detail

          Test of proportional-hazards assumption

          Time:  Time
```

	rho	chi2	df	Prob>chi2
PSI	0.44688	2.25	1	0.1336
ProADM	-0.38731	2.38	1	0.1229
ProBNP	0.05734	0.03	1	0.8541
global test		2.56	3	0.4652

```
250 . linktest

         failure _d:  MortGlo == 1
   analysis time _t:  TSup

Iteration 0:   log likelihood = -53.958128
Iteration 1:   log likelihood = -43.334243
Iteration 2:   log likelihood = -39.816723
Iteration 3:   log likelihood = -39.419999
Iteration 4:   log likelihood = -39.028421
Iteration 5:   log likelihood = -39.028381
Refining estimates:
Iteration 0:   log likelihood = -39.028381

Cox regression -- no ties

No. of subjects =          225                Number of obs   =        225
No. of failures =           10
Time at risk    =        19722
                                              LR chi2(2)      =      29.86
Log likelihood  =   -39.028381                Prob > chi2     =     0.0000
```

_t	Coef.	Std. Err.	z	P>|z|	[95% Conf.	Interval]
_hat	1.197366	.3723702	3.22	0.001	.4675336	1.927198
_hatsq	-.0312328	.0410982	-0.76	0.447	-.1117837	.0493182

```
251 . stcox ProADM

         failure _d:  MortGlo == 1
   analysis time _t:  TSup

Iteration 0:   log likelihood = -54.003385
Iteration 1:   log likelihood = -45.363774
Iteration 2:   log likelihood = -45.013066
Iteration 3:   log likelihood = -39.980036
Iteration 4:   log likelihood = -39.766014
Refining estimates:
Iteration 0:   log likelihood = -39.766014

Cox regression -- no ties

No. of subjects =          226                Number of obs   =        226
No. of failures =           10
Time at risk    =        19812
                                              LR chi2(1)      =      28.47
Log likelihood  =   -39.766014                Prob > chi2     =     0.0000
```

_t	Haz. Ratio	Std. Err.	z	P>|z|	[95% Conf.	Interval]
ProADM	2.695595	.5613562	4.76	0.000	1.792225	4.054309

```
252 . estat phtest,detail

      Test of proportional-hazards assumption

      Time:  Time
```

	rho	chi2	df	Prob>chi2
ProADM	-0.23410	0.34	1	0.5577
global test		0.34	1	0.5577

```
253 . linktest

         failure _d:  MortGlo == 1
   analysis time _t:  TSup

Iteration 0:   log likelihood = -54.003385
Iteration 1:   log likelihood = -43.976253
Iteration 2:   log likelihood = -40.533057
Iteration 3:   log likelihood = -39.614717
Iteration 4:   log likelihood = -39.602065
Iteration 5:   log likelihood = -39.601596
Iteration 6:   log likelihood = -39.601595
Refining estimates:
Iteration 0:   log likelihood = -39.601595

Cox regression -- no ties

No. of subjects =          226                 Number of obs   =        226
No. of failures =           10
Time at risk    =        19812
                                               LR chi2(2)      =      28.80
Log likelihood  =   -39.601595                 Prob > chi2     =     0.0000
```

_t	Coef.	Std. Err.	z	P>|z|	[95% Conf.	Interval]
_hat	1.220755	.3954372	3.09	0.002	.4457127	1.995798
_hatsq	-.0330795	.0424161	-0.78	0.435	-.1162136	.0500546

```
254 . stcox  PSI

         failure _d:  MortGlo == 1
   analysis time _t:  TSup

Iteration 0:   log likelihood = -54.003385
Iteration 1:   log likelihood = -47.807809
Iteration 2:   log likelihood = -46.260656
Iteration 3:   log likelihood = -46.247533
Refining estimates:
Iteration 0:   log likelihood = -46.247533

Cox regression -- no ties

No. of subjects =          226                 Number of obs   =        226
No. of failures =           10
Time at risk    =        19812
                                               LR chi2(1)      =      15.51
Log likelihood  =   -46.247533                 Prob > chi2     =     0.0001
```

_t	Haz. Ratio	Std. Err.	z	P>|z|	[95% Conf.	Interval]
PSI	1.025817	.0062398	4.19	0.000	1.01366	1.03812

```
255 . estat phtest,detail

      Test of proportional-hazards assumption

      Time:  Time
```

	rho	chi2	df	Prob>chi2
PSI	-0.15222	0.07	1	0.7980
global test		0.07	1	0.7980

```
256 . linktest

         failure _d:  MortGlo == 1
   analysis time _t:  TSup

Iteration 0:   log likelihood = -54.003385
Iteration 1:   log likelihood = -47.908267
Iteration 2:   log likelihood = -43.869322
Iteration 3:   log likelihood = -42.703868
Iteration 4:   log likelihood = -42.511934
Iteration 5:   log likelihood = -42.504144
Iteration 6:   log likelihood = -42.504126
Refining estimates:
Iteration 0:   log likelihood = -42.504126

Cox regression -- no ties

No. of subjects =          226                Number of obs    =       226
No. of failures =           10
Time at risk    =        19812
                                               LR chi2(2)       =     23.00
Log likelihood  =   -42.504126                 Prob > chi2      =    0.0000
```

_t	Coef.	Std. Err.	z	P>|z|	[95% Conf. Interval]	
_hat	7.235928	3.207404	2.26	0.024	.949532	13.52232
_hatsq	-.8553006	.4404011	-1.94	0.052	-1.718471	.0078697

```
257 . stcox  PSI ProADM

         failure _d:  MortGlo == 1
   analysis time _t:  TSup

Iteration 0:   log likelihood = -54.003385
Iteration 1:   log likelihood = -45.870849
Iteration 2:   log likelihood = -39.854599
Iteration 3:   log likelihood = -39.742811
Iteration 4:   log likelihood = -39.742696
Refining estimates:
Iteration 0:   log likelihood = -39.742696

Cox regression -- no ties

No. of subjects =          226                Number of obs    =       226
No. of failures =           10
Time at risk    =        19812
                                               LR chi2(2)       =     28.52
Log likelihood  =   -39.742696                 Prob > chi2      =    0.0000
```

_t	Haz. Ratio	Std. Err.	z	P>|z|	[95% Conf. Interval]	
PSI	.9973358	.0123249	-0.22	0.829	.9734696	1.021787
ProADM	2.930431	1.295154	2.43	0.015	1.232335	6.968423

```
258 . estat phtest,detail

      Test of proportional-hazards assumption

      Time:  Time
```

	rho	chi2	df	Prob>chi2
PSI	0.46582	2.34	1	0.1262
ProADM	-0.41166	2.62	1	0.1054
global test		2.67	2	0.2631

```
259 . linktest

         failure _d:  MortGlo == 1
   analysis time _t:  TSup

Iteration 0:   log likelihood = -54.003385
Iteration 1:   log likelihood =  -43.87694
Iteration 2:   log likelihood = -40.364161
Iteration 3:   log likelihood = -39.761304
Iteration 4:   log likelihood = -39.640875
Iteration 5:   log likelihood = -39.622272
Iteration 6:   log likelihood = -39.620903
Iteration 7:   log likelihood = -39.620889
Refining estimates:
Iteration 0:   log likelihood = -39.620889

Cox regression -- no ties

No. of subjects =          226                Number of obs   =        226
No. of failures =           10
Time at risk    =        19812
                                              LR chi2(2)      =      28.76
Log likelihood  =   -39.620889                Prob > chi2     =     0.0000
```

_t	Coef.	Std. Err.	z	P>|z|	[95% Conf.	Interval]
_hat	1.189456	.3849466	3.09	0.002	.4349745	1.943938
_hatsq	-.0305385	.0435704	-0.70	0.483	-.1159349	.054858

```
260 .
261 . logistic MortGlo PSI ProADM

Logistic regression                               Number of obs   =        226
                                                  LR chi2(2)      =      24.36
                                                  Prob > chi2     =     0.0000
Log likelihood = -28.775327                       Pseudo R2       =     0.2974
```

MortGlo	Odds Ratio	Std. Err.	z	P>|z|	[95% Conf.	Interval]
PSI	1.002902	.0133004	0.22	0.827	.9771692	1.029312
ProADM	2.80831	1.250049	2.32	0.020	1.173692	6.719487
_cons	.004331	.0045528	-5.18	0.000	.0005518	.033993

```
262 . predict MortGlo_Pre, p

263 . roctab MortGlo MortGlo_Pre, detail graph

Detailed report of sensitivity and specificity
```

Cutpoint	Sensitivity	Specificity	Correctly Classified	LR+	LR-
(>= .006675)	100.00%	0.00%	4.42%	1.0000	
(>= .0066..)	100.00%	0.46%	4.87%	1.0047	0.0000
(>= .0068..)	100.00%	0.93%	5.31%	1.0093	0.0000
(>= .0069..)	100.00%	1.39%	5.75%	1.0141	0.0000
(>= .0070..)	100.00%	1.85%	6.19%	1.0189	0.0000
(>= .0071..)	100.00%	2.31%	6.64%	1.0237	0.0000
(>= .0071..)	100.00%	2.78%	7.08%	1.0286	0.0000
(>= .00726)	100.00%	3.24%	7.52%	1.0335	0.0000
(>= .0073..)	100.00%	3.70%	7.96%	1.0385	0.0000
(>= .0074..)	100.00%	4.17%	8.41%	1.0435	0.0000
(>= .0075..)	100.00%	4.63%	8.85%	1.0485	0.0000
(>= .0075..)	100.00%	5.09%	9.29%	1.0537	0.0000
(>= .0076..)	100.00%	5.56%	9.73%	1.0588	0.0000
(>= .0077..)	100.00%	6.02%	10.18%	1.0640	0.0000
(>= .0078..)	100.00%	6.48%	10.62%	1.0693	0.0000
(>= .0079..)	100.00%	6.94%	11.06%	1.0746	0.0000

(>= .0080..)	100.00%	7.41%	11.50%	1.0800	0.0000
(>= .0081..)	100.00%	7.87%	11.95%	1.0854	0.0000
(>= .0081..)	100.00%	8.33%	12.39%	1.0909	0.0000
(>= .0082..)	100.00%	8.80%	12.83%	1.0964	0.0000
(>= .0082..)	100.00%	9.26%	13.27%	1.1020	0.0000
(>= .0082..)	100.00%	9.72%	13.72%	1.1077	0.0000
(>= .0082..)	100.00%	10.19%	14.16%	1.1134	0.0000
(>= .0082..)	100.00%	10.65%	14.60%	1.1192	0.0000
(>= .0083..)	100.00%	11.11%	15.04%	1.1250	0.0000
(>= .0083..)	100.00%	11.57%	15.49%	1.1309	0.0000
(>= .0084..)	100.00%	12.04%	15.93%	1.1368	0.0000
(>= .0084..)	100.00%	12.50%	16.37%	1.1429	0.0000
(>= .0084..)	100.00%	12.96%	16.81%	1.1489	0.0000
(>= .0085..)	100.00%	13.43%	17.26%	1.1551	0.0000
(>= .0087..)	100.00%	13.89%	17.70%	1.1613	0.0000
(>= .0089..)	100.00%	14.35%	18.14%	1.1676	0.0000
(>= .0089..)	100.00%	14.81%	18.58%	1.1739	0.0000
(>= .0089..)	100.00%	15.28%	19.03%	1.1803	0.0000
(>= .009261)	100.00%	15.74%	19.47%	1.1868	0.0000
(>= .0093..)	100.00%	16.20%	19.91%	1.1934	0.0000
(>= .0094..)	100.00%	16.67%	20.35%	1.2000	0.0000
(>= .0095..)	100.00%	17.13%	20.80%	1.2067	0.0000
(>= .0095..)	100.00%	17.59%	21.24%	1.2135	0.0000
(>= .0095..)	100.00%	18.06%	21.68%	1.2203	0.0000
(>= .0095..)	100.00%	18.52%	22.12%	1.2273	0.0000
(>= .0096..)	100.00%	18.98%	22.57%	1.2343	0.0000
(>= .0096..)	100.00%	19.44%	23.01%	1.2414	0.0000
(>= .0098..)	100.00%	19.91%	23.45%	1.2486	0.0000
(>= .0098..)	100.00%	20.37%	23.89%	1.2558	0.0000
(>= .0099..)	100.00%	20.83%	24.34%	1.2632	0.0000
(>= .0099..)	100.00%	21.30%	24.78%	1.2706	0.0000
(>= .0102..)	100.00%	21.76%	25.22%	1.2781	0.0000
(>= .0104..)	100.00%	22.22%	25.66%	1.2857	0.0000
(>= .0107..)	100.00%	22.69%	26.11%	1.2934	0.0000
(>= .0108..)	100.00%	23.15%	26.55%	1.3012	0.0000
(>= .0109..)	100.00%	23.61%	26.99%	1.3091	0.0000
(>= .0110..)	100.00%	24.07%	27.43%	1.3171	0.0000
(>= .011064)	100.00%	24.54%	27.88%	1.3252	0.0000
(>= .0111..)	100.00%	25.00%	28.32%	1.3333	0.0000
(>= .0111..)	100.00%	25.46%	28.76%	1.3416	0.0000
(>= .0111..)	100.00%	25.93%	29.20%	1.3500	0.0000
(>= .011212)	100.00%	26.39%	29.65%	1.3585	0.0000
(>= .0113..)	100.00%	26.85%	30.09%	1.3671	0.0000
(>= .0113..)	100.00%	27.31%	30.53%	1.3758	0.0000
(>= .0114..)	100.00%	27.78%	30.97%	1.3846	0.0000
(>= .0114..)	100.00%	28.24%	31.42%	1.3935	0.0000
(>= .0115..)	100.00%	28.70%	31.86%	1.4026	0.0000
(>= .0115..)	100.00%	29.17%	32.30%	1.4118	0.0000
(>= .0117..)	100.00%	29.63%	32.74%	1.4211	0.0000
(>= .0117..)	100.00%	30.09%	33.19%	1.4305	0.0000
(>= .0118..)	100.00%	30.56%	33.63%	1.4400	0.0000
(>= .0118..)	100.00%	31.02%	34.07%	1.4497	0.0000
(>= .0118..)	100.00%	31.48%	34.51%	1.4595	0.0000
(>= .0120..)	100.00%	31.94%	34.96%	1.4694	0.0000
(>= .0120..)	100.00%	32.41%	35.40%	1.4795	0.0000
(>= .0121..)	100.00%	32.87%	35.84%	1.4897	0.0000
(>= .0124..)	100.00%	33.33%	36.28%	1.5000	0.0000
(>= .0126..)	100.00%	33.80%	36.73%	1.5105	0.0000
(>= .0126..)	100.00%	34.26%	37.17%	1.5211	0.0000
(>= .0127..)	100.00%	34.72%	37.61%	1.5319	0.0000
(>= .0127..)	100.00%	35.19%	38.05%	1.5429	0.0000
(>= .0128..)	100.00%	35.65%	38.50%	1.5540	0.0000
(>= .0129..)	100.00%	36.11%	38.94%	1.5652	0.0000
(>= .0133..)	100.00%	36.57%	39.38%	1.5766	0.0000
(>= .0134..)	100.00%	37.04%	39.82%	1.5882	0.0000
(>= .0134..)	100.00%	37.50%	40.27%	1.6000	0.0000
(>= .0137..)	100.00%	37.96%	40.71%	1.6119	0.0000
(>= .0137..)	100.00%	38.43%	41.15%	1.6241	0.0000
(>= .0138..)	100.00%	38.89%	41.59%	1.6364	0.0000
(>= .0138..)	100.00%	39.35%	42.04%	1.6489	0.0000
(>= .0138..)	100.00%	39.81%	42.48%	1.6615	0.0000
(>= .013965)	100.00%	40.28%	42.92%	1.6744	0.0000

(>= .01459)	100.00%	40.74%	43.36%	1.6875	0.0000
(>= .0146..)	100.00%	41.20%	43.81%	1.7008	0.0000
(>= .0146..)	100.00%	41.67%	44.25%	1.7143	0.0000
(>= .0147..)	100.00%	42.13%	44.69%	1.7280	0.0000
(>= .0147..)	100.00%	42.59%	45.13%	1.7419	0.0000
(>= .0149..)	100.00%	43.06%	45.58%	1.7561	0.0000
(>= .0150..)	100.00%	43.52%	46.02%	1.7705	0.0000
(>= .0152..)	100.00%	43.98%	46.46%	1.7851	0.0000
(>= .0153..)	100.00%	44.44%	46.90%	1.8000	0.0000
(>= .0154..)	100.00%	44.91%	47.35%	1.8151	0.0000
(>= .015558)	100.00%	45.37%	47.79%	1.8305	0.0000
(>= .0156..)	100.00%	46.30%	48.67%	1.8621	0.0000
(>= .0157..)	100.00%	46.76%	49.12%	1.8783	0.0000
(>= .0157..)	100.00%	47.22%	49.56%	1.8947	0.0000
(>= .0159..)	100.00%	47.69%	50.00%	1.9115	0.0000
(>= .0159..)	100.00%	48.15%	50.44%	1.9286	0.0000
(>= .0160..)	100.00%	48.61%	50.88%	1.9459	0.0000
(>= .0161..)	100.00%	49.07%	51.33%	1.9636	0.0000
(>= .0161..)	100.00%	49.54%	51.77%	1.9817	0.0000
(>= .0163..)	100.00%	50.00%	52.21%	2.0000	0.0000
(>= .0164..)	100.00%	50.46%	52.65%	2.0187	0.0000
(>= .0169..)	100.00%	50.93%	53.10%	2.0377	0.0000
(>= .0169..)	100.00%	51.39%	53.54%	2.0571	0.0000
(>= .0169..)	100.00%	51.85%	53.98%	2.0769	0.0000
(>= .0170..)	100.00%	52.31%	54.42%	2.0971	0.0000
(>= .0170..)	100.00%	52.78%	54.87%	2.1176	0.0000
(>= .0171..)	100.00%	53.24%	55.31%	2.1386	0.0000
(>= .0172..)	100.00%	53.70%	55.75%	2.1600	0.0000
(>= .0174..)	100.00%	54.17%	56.19%	2.1818	0.0000
(>= .0174..)	100.00%	54.63%	56.64%	2.2041	0.0000
(>= .0175..)	100.00%	55.09%	57.08%	2.2268	0.0000
(>= .0179..)	100.00%	55.56%	57.52%	2.2500	0.0000
(>= .0179..)	100.00%	56.02%	57.96%	2.2737	0.0000
(>= .0180..)	100.00%	56.48%	58.41%	2.2979	0.0000
(>= .0180..)	100.00%	56.94%	58.85%	2.3226	0.0000
(>= .0182..)	100.00%	57.41%	59.29%	2.3478	0.0000
(>= .0184..)	100.00%	57.87%	59.73%	2.3736	0.0000
(>= .0188..)	100.00%	58.33%	60.18%	2.4000	0.0000
(>= .0189..)	100.00%	58.80%	60.62%	2.4270	0.0000
(>= .0192..)	100.00%	59.26%	61.06%	2.4545	0.0000
(>= .0192..)	100.00%	59.72%	61.50%	2.4828	0.0000
(>= .0195..)	100.00%	60.19%	61.95%	2.5116	0.0000
(>= .019523)	100.00%	60.65%	62.39%	2.5412	0.0000
(>= .0196..)	100.00%	61.11%	62.83%	2.5714	0.0000
(>= .0197..)	100.00%	61.57%	63.27%	2.6024	0.0000
(>= .0198..)	90.00%	61.57%	62.83%	2.3422	0.1624
(>= .0200..)	90.00%	62.04%	63.27%	2.3707	0.1612
(>= .020201)	90.00%	62.50%	63.72%	2.4000	0.1600
(>= .0204..)	90.00%	62.96%	64.16%	2.4300	0.1588
(>= .0204..)	90.00%	63.43%	64.60%	2.4608	0.1577
(>= .0207..)	90.00%	63.89%	65.04%	2.4923	0.1565
(>= .0207..)	90.00%	64.35%	65.49%	2.5247	0.1554
(>= .0208..)	90.00%	64.81%	65.93%	2.5579	0.1543
(>= .0208..)	90.00%	65.28%	66.37%	2.5920	0.1532
(>= .0210..)	90.00%	65.74%	66.81%	2.6270	0.1521
(>= .0211..)	90.00%	66.20%	67.26%	2.6630	0.1510
(>= .0215..)	90.00%	66.67%	67.70%	2.7000	0.1500
(>= .0219..)	90.00%	67.13%	68.14%	2.7380	0.1490
(>= .0221..)	90.00%	67.59%	68.58%	2.7771	0.1479
(>= .0222..)	80.00%	67.59%	68.14%	2.4686	0.2959
(>= .0224..)	80.00%	68.06%	68.58%	2.5043	0.2939
(>= .022838)	80.00%	68.52%	69.03%	2.5412	0.2919
(>= .0233..)	80.00%	68.98%	69.47%	2.5791	0.2899
(>= .0236..)	80.00%	69.44%	69.91%	2.6182	0.2880
(>= .0237..)	80.00%	69.91%	70.35%	2.6585	0.2861
(>= .0241..)	80.00%	70.37%	70.80%	2.7000	0.2842
(>= .0251..)	80.00%	70.83%	71.24%	2.7429	0.2824
(>= .0251..)	80.00%	71.30%	71.68%	2.7871	0.2805
(>= .0252..)	80.00%	71.76%	72.12%	2.8328	0.2787
(>= .0253..)	80.00%	72.22%	72.57%	2.8800	0.2769
(>= .0260..)	80.00%	72.69%	73.01%	2.9288	0.2752
(>= .0266..)	80.00%	73.15%	73.45%	2.9793	0.2734

(>= .026703)	80.00%	73.61%	73.89%	3.0316	0.2717
(>= .0267..)	80.00%	74.54%	74.78%	3.1418	0.2683
(>= .0270..)	80.00%	75.00%	75.22%	3.2000	0.2667
(>= .0274..)	80.00%	75.46%	75.66%	3.2604	0.2650
(>= .0275..)	80.00%	75.93%	76.11%	3.3231	0.2634
(>= .0276..)	80.00%	76.39%	76.55%	3.3882	0.2618
(>= .0278..)	80.00%	76.85%	76.99%	3.4560	0.2602
(>= .0280..)	80.00%	77.31%	77.43%	3.5265	0.2587
(>= .028577)	80.00%	77.78%	77.88%	3.6000	0.2571
(>= .0288..)	80.00%	78.24%	78.32%	3.6766	0.2556
(>= .0290..)	80.00%	78.70%	78.76%	3.7565	0.2541
(>= .0290..)	80.00%	79.17%	79.20%	3.8400	0.2526
(>= .0295..)	80.00%	79.63%	79.65%	3.9273	0.2512
(>= .0300..)	80.00%	80.09%	80.09%	4.0186	0.2497
(>= .0301..)	80.00%	80.56%	80.53%	4.1143	0.2483
(>= .030815)	80.00%	81.02%	80.97%	4.2146	0.2469
(>= .0309..)	80.00%	81.48%	81.42%	4.3200	0.2455
(>= .0313..)	80.00%	81.94%	81.86%	4.4308	0.2441
(>= .0316..)	80.00%	82.41%	82.30%	4.5474	0.2427
(>= .0321..)	80.00%	82.87%	82.74%	4.6703	0.2413
(>= .0323..)	80.00%	83.33%	83.19%	4.8000	0.2400
(>= .0323..)	80.00%	83.80%	83.63%	4.9371	0.2387
(>= .033251)	80.00%	84.26%	84.07%	5.0824	0.2374
(>= .0346..)	80.00%	84.72%	84.51%	5.2364	0.2361
(>= .0347..)	80.00%	85.19%	84.96%	5.4000	0.2348
(>= .0353..)	80.00%	85.65%	85.40%	5.5742	0.2335
(>= .0400..)	80.00%	86.11%	85.84%	5.7600	0.2323
(>= .0445..)	80.00%	86.57%	86.28%	5.9586	0.2310
(>= .0469..)	70.00%	86.57%	85.84%	5.2138	0.3465
(>= .0473..)	70.00%	87.04%	86.28%	5.4000	0.3447
(>= .0499..)	70.00%	87.50%	86.73%	5.6000	0.3429
(>= .0502..)	70.00%	87.96%	87.17%	5.8154	0.3411
(>= .0546..)	70.00%	88.43%	87.61%	6.0480	0.3393
(>= .0568..)	70.00%	88.89%	88.05%	6.3000	0.3375
(>= .0573..)	70.00%	89.35%	88.50%	6.5739	0.3358
(>= .0588..)	70.00%	89.81%	88.94%	6.8727	0.3340
(>= .0614..)	70.00%	90.28%	89.38%	7.2000	0.3323
(>= .0616..)	70.00%	90.74%	89.82%	7.5600	0.3306
(>= .0633..)	70.00%	91.20%	90.27%	7.9579	0.3289
(>= .0645..)	60.00%	91.20%	89.82%	6.8211	0.4386
(>= .0653..)	60.00%	91.67%	90.27%	7.2000	0.4364
(>= .0678..)	50.00%	91.67%	89.82%	6.0000	0.5455
(>= .0685..)	50.00%	92.13%	90.27%	6.3529	0.5427
(>= .0729..)	50.00%	92.59%	90.71%	6.7500	0.5400
(>= .0740..)	50.00%	93.06%	91.15%	7.2000	0.5373
(>= .0807..)	50.00%	93.52%	91.59%	7.7143	0.5347
(>= .0811..)	50.00%	93.98%	92.04%	8.3077	0.5320
(>= .0873..)	50.00%	94.44%	92.48%	9.0000	0.5294
(>= .0967..)	50.00%	94.91%	92.92%	9.8182	0.5268
(>= .1140..)	50.00%	95.37%	93.36%	10.8000	0.5243
(>= .1389..)	50.00%	95.83%	93.81%	12.0000	0.5217
(>= .15357)	50.00%	96.30%	94.25%	13.5000	0.5192
(>= .1625..)	50.00%	96.76%	94.69%	15.4286	0.5167
(>= .1750..)	50.00%	97.22%	95.13%	18.0000	0.5143
(>= .2525..)	50.00%	97.69%	95.58%	21.6000	0.5118
(>= .2728..)	50.00%	98.15%	96.02%	27.0000	0.5094
(>= .3490..)	40.00%	98.15%	95.58%	21.6000	0.6113
(>= .3695..)	30.00%	98.15%	95.13%	16.2000	0.7132
(>= .4234..)	20.00%	98.15%	94.69%	10.8000	0.8151
(>= .4356..)	20.00%	98.61%	95.13%	14.4000	0.8113
(>= .468641)	20.00%	99.07%	95.58%	21.6000	0.8075
(>= .5185..)	10.00%	99.07%	95.13%	10.8000	0.9084
(>= .6279..)	10.00%	99.54%	95.58%	21.6002	0.9042
(>= .9998..)	10.00%	100.00%	96.02%		0.9000
(> .9998..)	0.00%	100.00%	95.58%		1.0000

Obs	ROC Area	Std. Err.	—Asymptotic Normal— [95% Conf.	Interval]
226	0.8921	0.0451	0.80371	0.98055

264 . roccomp MortGlo PSI ProADM

	Obs	ROC Area	Std. Err.	—Asymptotic Normal— [95% Conf.	Interval]
**PSI**	226	0.8900	0.0357	0.82007	0.96003
**ProADM**	226	0.8854	0.0486	0.79021	0.98062

Ho: area(**PSI**) = area(**ProADM**)
    chi2(**1**) = 0.01   Prob>chi2 = 0.9034

265 . roccomp MortGlo PSI MortGlo_Pre

	Obs	ROC Area	Std. Err.	—Asymptotic Normal— [95% Conf.	Interval]
**PSI**	226	0.8900	0.0357	0.82007	0.96003
**MortGlo_Pre**	226	0.8921	0.0451	0.80371	0.98055

Ho: area(**PSI**) = area(**MortGlo_Pre**)
    chi2(**1**) = 0.00   Prob>chi2 = 0.9524

266 .
267 . nri3 MortGlo PSI, prvars(PSI ProADM) cut(2.8 8.2 29.3)

NRI	Estimate	Std. Err.	Z	P-value

MortGlo and Established risk factors	Established risk factors + new predictors <2.8%	2.8-8.2%	8.2 - 29.3%	>=29.3%	Total
1					
<2.8%					
2.8-8.2%	2	1			3
8.2 - 29.3%		2	1	3	6
>=29.3%				1	1
Total	2	3	1	4	10
0					
<2.8%	129	12			141
2.8-8.2%	35	14	2		51
8.2 - 29.3%	3	11	5	2	21
>=29.3%			1	2	3
Total	167	37	8	4	216

268 .
end of do-file

269 .
